# Cancer stem cells: advances in knowledge and implications for cancer therapy

**DOI:** 10.1038/s41392-024-01851-y

**Published:** 2024-07-05

**Authors:** Xianjing Chu, Wentao Tian, Jiaoyang Ning, Gang Xiao, Yunqi Zhou, Ziqi Wang, Zhuofan Zhai, Guilong Tanzhu, Jie Yang, Rongrong Zhou

**Affiliations:** 1 Department of Oncology, Xiangya Hospital, Central South University, Changsha, 410008 China; 2 Department of Dermatology, Xiangya Hospital, Central South University, Changsha, 410008 China; 3 Xiangya Lung Cancer Center, Xiangya Hospital, Central South University, Changsha, 410008 China; 4 National Clinical Research Center for Geriatric Disorders, Xiangya Hospital, Central South University, Changsha, Hunan Province 410008 China

**Keywords:** Cancer stem cells, Cancer therapy

## Abstract

Cancer stem cells (CSCs), a small subset of cells in tumors that are characterized by self-renewal and continuous proliferation, lead to tumorigenesis, metastasis, and maintain tumor heterogeneity. Cancer continues to be a significant global disease burden. In the past, surgery, radiotherapy, and chemotherapy were the main cancer treatments. The technology of cancer treatments continues to develop and advance, and the emergence of targeted therapy, and immunotherapy provides more options for patients to a certain extent. However, the limitations of efficacy and treatment resistance are still inevitable. Our review begins with a brief introduction of the historical discoveries, original hypotheses, and pathways that regulate CSCs, such as WNT/β-Catenin, hedgehog, Notch, NF-κB, JAK/STAT, TGF-β, PI3K/AKT, PPAR pathway, and their crosstalk. We focus on the role of CSCs in various therapeutic outcomes and resistance, including how the treatments affect the content of CSCs and the alteration of related molecules, CSCs-mediated therapeutic resistance, and the clinical value of targeting CSCs in patients with refractory, progressed or advanced tumors. In summary, CSCs affect therapeutic efficacy, and the treatment method of targeting CSCs is still difficult to determine. Clarifying regulatory mechanisms and targeting biomarkers of CSCs is currently the mainstream idea.

## Introduction

Due to advances in cancer early detection and cancer treatments, cancer yearly mortality has been decreasing since 1995.^1^ However, cancers still caused more deaths than COVID-19 and ranked as the second cause of death in the United States in 2020 and 2021.^1^ The presence of cancer stem cells (CSCs) can be an essential factor that leads to failure of cancer treatments.

CSCs, first identified in 1990,^2^ are a small group of cancer cells that possess properties of normal stem cells, such as self-renewal and pluripotency.^3^ The CSC model, also known as the hierarchical model, provides a paradigm for people to understand intratumoral heterogeneity, as they can differentiate into various phenotypes of cancer cells and maintain their population.^4^ CSCs are also characterized by enhanced ability to initiate tumor growth, proliferate, invade, migrate, and resist therapeutic effects.^3^ This implies a crucial role of CSCs in cancer development and makes CSCs an evaluable target for anti-cancer treatments. Therapeutic agents, such as monoclonal antibodies, tyrosine kinase inhibitors, chimeric antigen receptors (CAR) T cells, and tumor vaccines, targeting CSCs have been developed and tested in clinical trials.^5^

In recent years, studies have added knowledge in the origin, features, and especially therapeutic aspects of CSCs. Here, we summarize the research history, origins, properties, molecular regulations, mechanisms for therapeutic resistance, and treatment strategies of CSCs.

## The development of the CSCs theory

### The discovery and controversy of the CSCs

**Fig. 1:**
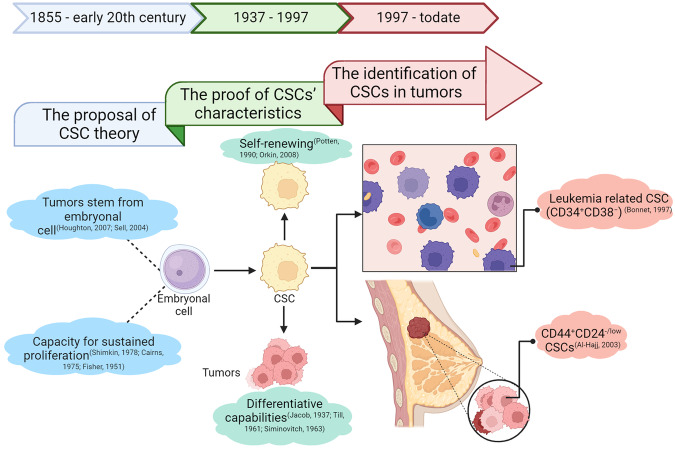
The Development of the CSC Theory. As early as 1855, in the discourse on the origins of tumors, Cohnheim posited that tumors stemmed from embryonic cells. Subsequent decades of genetic research concluded that tumor formation necessitates the accumulation of susceptibility genes, implying that the cells causing tumors must possess self-renewal capabilities. It wasn’t until 1937 that Furth demonstrated the potential of single malignant cells to induce tumors. This revelation spurred researchers to delve into the characteristics of such cells, encompassing self-renewal, aberrant differentiation, interaction with the microenvironment, and heightened plasticity. In 1997, John Dick identified leukemia stem cells. Since then, the theory of CSCs has basically taken shape. And people have begun to continuously isolate and prove CSCs from different tumor types

As early as 1855, the work of pathologist Rudolph Virchow illuminated that tumors stem from existing normal cells, sparking a scientific discourse about the origin of tumors (Fig. 1). Julius Cohnheim disagreed with that and contributed to his “embryonal cell rests” hypothesis in 1867.^6^ This posited that dormant embryonic cells within tissues could awaken into tumors.^7^ Spanning the 19th and 20th centuries, burgeoning research into the genetic underpinnings of cancer has fostered the prevailing notion that cancer arises from the accumulation of mutations in susceptible cells. However, given the terminal differentiation and quiescence of most body cells, their lifespan seldom permits the accrual of the requisite mutations to become cancerous.^8–10^ Hence, cells endowed with the capacity for sustained proliferation are the likely precursors of tumors. This hypothesis gained traction and was bolstered with the discovery of Jacob Furth and Morton Kahn in 1937, which leukemia could be recapitulated in mice from single malignant cells.^11^

The understanding of this field deepened when James Till and Ernest McCulloch, in 1961, observed clone formation in the spleen during hematopoietic regeneration.^12,13^ Moreover, these clones could form additional clones in other mice, laying the groundwork for understanding the self-renewing and differentiative capabilities of stem cells.^14,15^ Schofield introduced the definition of “stem cell niche” in 1978, and further nuanced this, highlighting the role of microenvironment in nourishing and directing stem cells.^16,17^ From the 1960s to the early 1990s, debates about the origin of tumors had oscillated between non-genetic “induction” or “niche destruction” versus proliferative single-cell mutations.^18–20^

The landscape of stem cell research was revolutionized in 1997 when John and Bonnet first identified cells with extensive proliferative potential in acute myeloid leukemia (AML) and isolated CSCs characterized by the CD34^+^CD38^−^ phenotype.^2^ This seminal breakthrough acknowledged the existence of leukemia stem cells and paved the way for the theory of “CSCs” in 2001.^21^ Tumors house a kind of rare cells with self-renewing potential named CSCs that drive tumorigenesis, akin to normal stem cells but with a role in cancer progression.^22^ The concept of CSCs has since expanded to various solid tumors. In 2003, Al-Hajj first isolated CD44^+^CD24^−/low^ CSCs from breast tumors, capable of significant tumorigenicity in mice. Their study had shown that 200 such cells could form transplanted tumors in recipient mice within 12 weeks. In the same culture time, 10,000 non-special breast cancer cells cannot form tumors.^23^ In the same year, Sheila K Singh purificated a CD133^+^ CSC population from diverse brain tumors.^23,24^ The discovery of these potent CSCs across both hematologic and solid malignancies has substantiated the CSC theory, with subsequent findings in prostate, colorectal, pancreatic, nasopharyngeal cancers, and so on.^25–30^

Despite widespread support and experimental evidence for the theory of CSCs, xenotransplantation success rates and actual CSC percentages have fallen short of expectations.^31^ For instance, only a small fraction (<5%) of leukemia transplants in mice, as seen in Jacob Furth’s studies, successfully engrafted.^11^ Hewitt’s research further corroborates the scarcity of successful transplantation, with colony formation in murine spleens documented at a mere 1% to 4%.^32^ Moreover, Park et al. in vitro clonal cultures from myeloma cells extracted from murine ascites exhibited clonal colony formation in only 0.01% to 1%.^33^ Analogous low successful frequencies of tumorigenic cells are reported in vitro cultures of lung, ovarian, and neuroblastoma cancers.^34^ Concurrently, more and more research reveals the diversity and instability of CSCs, with variations in cell origin, proportion, genetic makeup, and even phenotypic and functional traits.^35–37^ Initially, CSCs exhibiting the CD34^+^CD38^−^ phenotype were linked to the etiology of AML. Subsequent findings, however, indicated that CD34^+^CD38^+^ cells also possess tumor-initiating potential in NOD/SCID mice lacking the Interleukin 2 Receptor Subunit Gamma (IL2RG) chain, suggesting that such activity might be independent of CD38 expression.^2,38^ This phenomenon is mirrored in solid tumors, where certain CSC populations within the same tumor display distinct and non-overlapping marker profiles. Ginestier et al.‘s research in breast cancer revealed that cells exhibiting high Aldehyde Dehydrogenase (ALDH) activity not only demonstrated traits of tumorigenicity but also the capacity to self-renew and replicate the heterogeneity of the original tumor.^39^ These cells also displayed minimal overlap with the previously characterized CD44^+^CD24^−/low^ phenotype breast CSCs, constituting less than 1% of the cancer cell population.^39^ Research suggests that CD133 is a marker capable of identifying CSC populations across various solid tumors, including different forms of brain cancer.^40–43^ However, subsequent studies have raised questions regarding the reliability of using CD133 to distinguish and isolate CSCs, indicating a degree of controversy in its application.^44–46^ Firstly, CD133 may serve as a marker for glandular epithelium in certain tissues, complicating the distinction between CSCs and non-stem-like cancer cells. Secondly, research has demonstrated that CD133^+^ cell populations fail to replicate the morphology of the original upon xenotransplantation, suggesting the possibility of expression of CD133 on normal differentiated cells. Lastly, studies have shown that CD133^−/low^ populations have been shown to recapitulate the original tumor architecture, indicating that CD133 may not be the sole marker for identifying CSCs.^47–49^ Moreover, plasma cells expressing the CD138 phenotype were found to only induce multiple myeloma (MM) in SCID-hu mice, failing to generate comparable tumors in NOD/SCID mice.^25,50–52^ Similarly, analyses of samples from AML patients revealed distinct genetic and phenotypic characteristics of CSCs among individuals, highlighting the variability within the CSC population across different models and patient samples.^53,54^ The proportion of CSCs within primary tumors is also highly variable, ranging from 0.2% to 82.5%.^35^ For instance, CSCs with the CD34^+^ phenotype constitute less than 1% of AML cases, yet represent 82.5% in B-cell precursor acute lymphoblastic leukemia (ALL).^2,38,55^ Conversely, the proportion of CSCs expressing the CD133^+^ phenotype in lung cancer ranges from a mere 0.4% to 1.5%, while in brain tumors and colorectal cancer, it can escalate to as high as 20%.^24,56,57^ Furthermore, the frequency of CSCs may increase during tumor progression. Pece’s study found a higher proportion of CSCs in stage III breast cancer compared to stage I about 3 to 4 times on average.^58^ The origins of CSCs remain elusive, with evidence from myeloid leukemia and brain tumors suggesting they may arise from normal stem cells, while findings from MM and ALL suggest alternative origins.^2,24,42,59,60^ The definition of CSCs becomes increasingly nebulous, raising doubts about the model itself. However, the methods used at that time could not account for cellular heterogeneity and proliferative potential within different tumor cell populations.^61,62^ Moreover, there was heterogeneity in the analytical methods used.^63,64^ Thus, debates over the CSCs model will persist until direct empirical evidence is presented. Nonetheless, the validity of the model should not be discounted due to the diversity and complexity that continue to emerge in experimental evidence.

### Advances in key technologies: from sorting to sequencing

The hypothesis of CSCs offers a pivotal theoretical framework for understanding tumor initiation and progression. Initially considered rare and dormant, forming a unidirectional hierarchy within tumors, CSCs were thought to generate all cell types within a tumor, occupying the apex of the tumor cell hierarchy.^65^ However, further research has revealed the model of CSCs to be more complex and dynamic. CSCs exhibit phenotypic plasticity, transforming in response to the microenvironment, leading to genetically heterogeneous tumors.^66–68^ Competitive interactions among various related but distinct subclones within tumors favor subpopulations with enhanced self-renewal capabilities and therapeutic resistance.^35^ Initial research propelled by traditional cell sorting techniques, advancements, especially in sequencing technologies, have continually enriched and evolved our understanding of the CSC model. The evolution of cell sorting technologies has progressed from utilizing physical properties of cells, such as size, density, adhesiveness, and refractivity, to targeting cell surface antigen phenotypes and functional characteristics like dye efflux, calcium ion concentration, and pH.^69^ Techniques include density gradient centrifugation, fluorescence-activated cell sorting (FACS), magnetic-activated cell sorting (MACS), and side population (SP) cell sorting (Table 1).

**Table 1:** Methods of isolating cancer stem cells

Technologies	Principle	Limitations
Density gradient centrifugation	Differences in size and density^70,71^	The toxicity of medium^72^
FACS	Antigen–antibody hybrid^73^	Requiring a lot of cells^75,76^
MACS	Antigen–antibody hybrid^73^	Complex operation^74,75^
SP cells sorting	Hoechst 33342 dye & Microexamination/flow cytometry^77^	Low separation efficiency^84^
RNA-sequencing	Transcription	Overlooking signals from crucial cells^102^
Single-cell sequencing	Transcription	Overlooking spatial differences^103^

While density gradient centrifugation was initially designed for isolating mononuclear peripheral blood cells, its application quickly extended to stem cell separation.^70,71^ Compared with a single-layer density gradient, the method of using a multi-layer discontinuous density gradient can separate CSCs more effectively. Percoll, a colloidal silica coated with polyvinylpyrrolidone (PVP), is preferred, though the toxicity of PVP in percoll limits its clinical safety.^72^

In isolating CSCs, methods based on cell surface markers are prevalent, notably FACS and MACS.^73^ MACS employs antibodies attached to magnetic beads to target cell membrane antigens, using magnetism to retain cells bound to beads within a column while unbound cells are washed away.^74^ Despite its minimal impact on cell viability and suitability for large-scale sorting, MACS is limited by its reliance on single antigens, complex operation, and high costs.^75^ FACS, on the other hand, utilizes fluorescently labeled antibodies to distinguish between CSCs and non-CSCs, offering higher specificity by screening multiple markers simultaneously. FACS can assess intracellular pathways and protein interactions and overcome the specificity challenges of CSC membrane antigens.^76^ However, FACS requires stringent experimental conditions and precise cell pretreatment to maintain cell viability, posing challenges in terms of equipment cost and operational requirements.^75^

The SP cell sorting method identifies CSCs using the Hoechst 33342 dye, capable of penetrating cell membranes.^77^ Since the discovery of Goodell et al. in bone marrow studies in 1996, this technique has proven effective across various tumor cell lines.^78–80^ SP cells, capable of asymmetric division and self-renewal, align with the characteristics of CSCs, suggesting SP sorting as a viable CSC enrichment strategy. Despite its simplicity, requiring only microscopy or flow cytometry to detect unstained cells, challenges include low separation efficiency and dye cytotoxicity. Nonetheless, its utility in sorting drug-resistant CSCs offers valuable insights for novel drug research. Researches indicated that when the activity of ABC transporters, such as ATP-Binding Cassette Transporter G2 (ABCG2), was inhibited, the SP phenotype cells decreased.^81,82^ Conversely, an increase in expression lead to an augmentation of the SP phenotype cells.^83^ Consequently, some researchers suggest that SP cells are not CSCs but rather a subset of cells capable of evading the cytotoxic effects of Hoechst dye.^84^

Beyond the aforementioned methods, alternative approaches for isolating and identifying CSCs exist.^85^ Drug selection separation gradually evolves cells towards drug resistance, isolating those capable of stable growth and passage, believed by some researchers to be CSCs.^86,87^ Western blot analysis serves as a traditional identification method, prized for its simplicity, universality, and cost-effectiveness, though it risks false positives or negatives if improperly executed, typically serving as a technique for validation. In 2021, Han et al. developed a novel label-free, microfluidic technology for CSC sorting based on physical characteristics like size, elasticity, and adhesiveness, enabling stable, rapid, and efficient CSC selection and enrichment, offering a new platform for targeted drug screening and functional identification.^88^

The integration of CSC sorting techniques with sequencing studies offers new insights into tumor complexity and heterogeneity. High-throughput technologies like RNA sequencing facilitate the monitoring of tumor microenvironment interactions and key gene expression dynamics. For instance, Chen discovered that under specific Trimethylation Of Lysine 4 On Histone H3 Protein Subunit (H3K4Me3) epigenetic modifications, the transcription factor MYC upregulates histidine decarboxylase, endowing glioblastoma stem cells (GSCs) with the ability to synthesize and secrete histamine. Histamine secreted by GSCs acts on the Histamine Type I Receptor (H1R) of vascular endothelial cells, activating the H1R/Ca^2+^/Nuclear Factor Kappa-B (NF-κB) signaling pathway to promote angiogenesis and advancing glioblastoma progression.^89^ The combination of advanced imaging, short hairpin RNA (shRNA) technology, and subgroup analysis tools has also highlighted the critical role of tumor-associated antigens in GSC differentiation.^90,91^ The development of multi-channel optical imaging systems has made it feasible to simultaneously monitor cell chemotaxis, proliferation, and NF-κB activity.^92^ In breast cancer, CSCs hyperactivate the Nuclear Respiratory Factors 2 (NRF2) pathway via the epigenetic reader Zinc Finger MYND-Type Containing 8 (ZMYND8), enhancing antioxidative capacity and evasion from oxidative damage and ferroptosis.^93^ Erythropoietin-Producing Hepatocellular Carcinoma Receptor B2 (EPHB2) and Lysine-Specific Histone Demethylase 1 (LSD1) are noted for their roles in promoting CSC generation and drug resistance in hepatocellular carcinoma and thyroid cancer, respectively. The sequencing technology found that they interact with the T Cell Factor 1(TCF1)/EPHB2/β-Catenin signal pathway and Wingless-Type MMTV Integration Site Family (WNT)/β-Catenin, respectively.^94,95^ Whole-genome sequencing of circular RNAs like circSLC4A7 has unveiled their interaction with Heat Shock Proteins 90 (HSP90), activating the Notch1 pathway and influencing gastric CSC progression.^96^ In addition to solid tumors, leukemia stem cells have also been found to undergo specific ribosomal RNA methylation (2’-O-methylation) modifications. This methylation pattern can reshape ribosome function and protein translation, allowing leukemia stem cells to preferentially translate amino acid transporters, which facilitates the cells’ uptake of amino acids in the environment, thus improving the self-renewal and function of leukemia stem cells.^97^ Sequencing studies were typically used to purify CSCs, focusing on molecular markers, which are involved in asymmetrical division, migration, and signaling pathways. Among them, *MYC*, *Octamer-Binding Transcription Factor 4 (OCT4)*, *Sex Determining Region Y 2 (SOX2)*, and *ALDH* are several key genes related to CSCs that are often focused on in research.^98–100^

The complexity of tumors transcends single malignant cells, encompassing a diverse array of cell types such as immune and stromal cells, thereby exhibiting significant intra- and inter-tumoral heterogeneity.^101^ While traditional transcriptomic analyses have provided valuable insights into tumor growth and evolution, they may overlook signals from crucial cell groups or states.^102^ These pivotal cellular states, including CSCs and immune cells relevant to treatment responses, are essential for understanding and treating tumors. To surmount this limitation, scientists are adopting advanced technologies like single-cell RNA analysis (scRNA-seq) and spatial transcriptomics. These methods offer a refined understanding at the cellular and molecular levels, unveiling new dimensions of complex interactions and heterogeneity within tumors, thereby opening new avenues for cancer research and therapeutic strategy development.^103^

Research into malignant gliomas has been at the forefront of single-cell analyses of brain tumor.^104^ Utilizing scRNA-seq, researchers have uncovered a spectrum of stemness and differentiation potential in primary glioblastoma cells, revealing the importance of expression programs like *POU domain, class 3, transcription factor 2 (POU3F2)*, *Nuclear Factor I A (NFIA)*, and *NFIB* in regulating stem-like phenotypes.^105^ Similar analyses of IDH mutant oligodendrogliomas and astrocytomas have disclosed comparable developmental hierarchies and gliogenic differentiation lineages, supporting the CSCs model. The model posits that the majority of cancer cells are well-differentiated, maintaining oligodendrocyte-like or astrocyte-like lineages, with a subset of undifferentiated cells exhibiting stem/progenitor traits.^106,107^ Interestingly, higher tumor grades are associated with an enrichment of proliferative stem-like glioma cells, suggesting a significant role for a minority of cancer cells in the growth and progression of IDH mutant gliomas.^108,109^ However, in primary H3K27M gliomas, lower differentiation correlates with a higher proportion of stem-like cells, indicating greater tumorigenic potential.^110^ Copy number variation (CNV) subclones and expression profiles inferred from scRNA-seq can also be used to study the relationship between genetic subclones and cellular state diversity within tumors.^111,112^ In IDH1 or IDH2 mutant human oligodendrogliomas, different CNV subclones exhibit similar cellular hierarchies, suggesting that cellular status is primarily determined by developmental programs.^106^ In contrast, IDH wild-type glioblastomas are characterized by four plastic and highly malignant cellular states, including neural progenitor cells (NPC-like), oligodendrocyte precursor-like cells (OPC-like), astrocytes like (AC-like) and mesenchymal-like (MES-like), and these states are not strictly determined by the CNV pattern.^113^

Beyond gliomas, CSCs-like subpopulations have been identified in other solid tumor types. In advanced prostate cancer, the growth of CSCs correlates with diminished androgen response and enhanced expression of cell cycle-related genes, promoting androgen-independent plasticity.^114^ In breast cancer, mesenchymal/stem-like tumor cells are present in patients who respond to Epidermal Growth Factor Receptor (EGFR) inhibitors, and an EGFR-high-expressing subpopulation displays enhanced stem-like characteristics, reflecting an EGFR-dependent hierarchy.^115^ Chung et al. found characterized expression features promoting metastatic progression in rare subgroups of primary triple-negative breast cancer via scRNA-seq, uncovering pronounced epithelial-mesenchymal transition (EMT) and stemness traits driving tumor advancement and metastasis.^116^ Similarly, metastatic breast cancer cells display overarching EMT and stemness characteristics, though with distinct marker gene expressions.^117^ The scRNA-seq analysis of hepatocellular carcinoma also reveals heterogeneity in phenotype, function, and transcriptome of CSCs.^118^ Velten et al. combined scRNA-seq with lineage tracing using nuclear and mitochondrial somatic mutations to identify leukemia stem cell gene expression programs in AML, marked by transcriptional dysregulation and co-expression of stem and myeloid priming genes.^119^ Differentiated AML cells express various immunoregulatory genes, inhibiting T-cell activity in vitro.^120^ In chronic myelogenous leukemia (CML), researchers identified unique molecular features of CSCs, revealing heterogeneity. A CSC subgroup in CML, characterized by distinct molecular traits, persists selectively during prolonged tyrosine kinase inhibitors (TKI) treatment, featuring quiescence-related gene expression and dysregulated genes and pathways.^121^ These insights deepen the understanding of cellular and molecular mechanisms underlying CML treatment resistance. Unlike other tumor cells, CSCs in head and neck tumors show extreme genomic instability, including chromosomal gains and losses.^122^ Ren et al. proposed a differentiation trajectory from CSC-like ductal cells to invasive ductal cells in pancreatic cancer, identifying five genes significantly associated with CSC prognosis.^123^ Wu et al. found common mutations in signaling pathway genes in different colorectal cancer cell clones, providing evidence for monoclonal CRC origin and subsequent subclonal evolution.^124,125^ Leung et al. demonstrated, through single-cell sequencing, exome sequencing, and targeted deep sequencing, that colorectal cancer metastasis follows a late dissemination model, with tumor cells evolving and acquiring mutations that enable clonal spread at the primary site.^126^

Research into CSCs is an evolving and deepening field. Despite challenges such as the lack of a clear definition for CSCs and the need for integrating various experimental methodologies, continuous research and technological advancements hold promise for a deeper understanding of cancer’s essence. This progress is anticipated to unveil novel strategies for cancer treatment, navigating through the complexities of tumor biology to illuminate new pathways for intervention.

## Overview of cancer stem cells

### Origin hypothesis of cancer stem cells

#### Differentiated cells

**Fig. 2:**
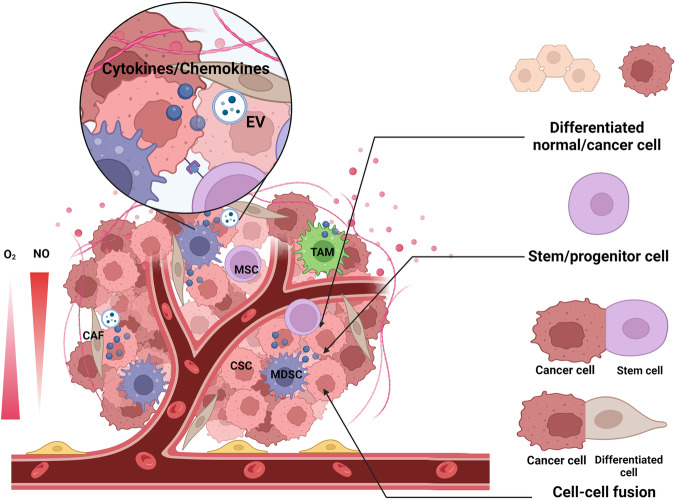
Origin, formation and/or maintenance of CSCs. CSCs originate from differentiated normal/cancer cells, stem/progenitor cells, or cell-cell fusion of cancer cells with stem cells or cancer cells with differentiated cells. The microenvironment of the CSC niche plays an essential role in the formation and maintenance of CSCs. MSCs, TAMs, MDSCs, and CAFs can secrete cytokines and chemokines that induce and/or maintain stem-like properties of cancer cells. Besides, CAFs can also modulate stemness by secreting EVs, and MSCs can regulate stemness through direct contact with CSCs. Finally, hypoxia and high nitric oxide (NO) concentration also support the CSC niche

Dedifferentiation is a reversed process by which differentiated cells return to a less differentiated stage within the same lineage.^127^ Dedifferentiation represents a common biological phenomenon in several physiological processes, such as cardiac regeneration and wound healing.^128^ By dedifferentiation, cells can gain stem-like properties, such as self-renewal and pluripotency, so this process also implies CSC formation and tumorigenesis (Fig. 2).^128^ Taking advantage of scRNA-seq and lineage tracing techniques, a study reveals a trajectory of dedifferentiation that PROM-1^+^ hepatocellular CSC follows, which strongly supports the role of dedifferentiation in CSC formation.^129^

Genetic or post-transcriptional alteration can lead to dedifferentiation of normal cells into CSCs. The combined loss of p16^INK4a^ and p19^ARF^ along with EGFR activation triggers the dedifferentiation of astrocytes and the genesis of glioblastoma.^130^ Besides astrocytes, terminally differentiated neurons can also undergo dedifferentiation to neural stem cell following shNF1-shp53 virus injection and induce gliomas.^131^ In intestinal crypts, ablation of Leucine-Rich Repeat-Containing G-Protein Coupled Receptor 5 (LGR5^+^) stem cells leads to dedifferentiation of daughter crypt cells and replenishment of stem cells, which is dependent on the transcription factor Achaete-Scute Homolog 2 (ASCL2).^132^ Mature pigment-producing melanocytes can also dedifferentiate into tumor progenitors of cutaneous melanoma induced by mutant BRAF.^133^ PGC7 induces promoter demethylation of transcription factors, such as *GLI1* and *MYCN*, and facilitates dedifferentiation of hepatocellular cancer cells.^134^ Downregulation or loss of Bcl3 leads to dedifferentiation of pancreatic cancer cells and expansion of the CSC population.^135^ A study shows that RNA slicing also plays an important part in tumor cell dedifferentiation, as the splicing factor SRSF1 maintains stemness in colorectal cancer.^136^ Also, downregulated miR-613 expression is associated with liver cell dedifferentiation and CSC formation, which is mediated by increased SOX9 expression.^137^

Several signaling pathways are involved in the regulation of dedifferentiation in terms of CSC formation. For instance, activation of the WNT pathway and loss of Sterile Alpha Motif Domain (Smad4) drive differentiated intestinal epithelium to stem cell-like status and initiate colon cancer growth.^138^ The WNT pathway is also associated with the dedifferentiation of breast cancer bone metastases into CSCs.^139^ Activation of NF-κΒ leads to enhancement of the WNT signaling, which further supports dedifferentiation of intestinal villus cells and acquisition of stem cell markers and tumor-initiating capabilities of these cells.^140^ The activation of the Transforming Growth Factor β (TGF-β) signaling pathway can also convert colorectal cancer cells into CSCs, which is dependent on the transcription factor Twist-Related Protein 1 (TWIST1).^141^ The activation of Hypoxia-Inducible Factor 1α (HIF-1α)/Notch pathway leads to dedifferentiation of pancreatic cancer cells and formation of stem-like cells.^142^ Extracellular Signal-Regulated Kinase (ERK) inhibition promotes cancer cell dedifferentiation and expands the CSC population in non-small cell lung cancer (NSCLC).^143^ Fibroblast-released IL-6, activin-A, and Granulocyte Colony-Stimulating Factor (G-CSF) induce Signal Transducer And Activator Of Transcription (STAT3) and Smad activation, which consequently activate the WNT, Notch, and hedgehog pathways and induce dedifferentiation of lung carcinoma cells.^144^

Environmental factors, including hypoxia, cytokines, and NO, also relate to the dedifferentiation in CSC formation. Under hypoxia, glioma, lung cancer, and hepatoma cells express high levels of stemness-associated transcription factors and CSC markers.^145^ In lung adenocarcinoma, CSCs can be formed through dedifferentiation induced by Insulin-Like Growth Factor-II (IGF-II) secreted from cancer-associated fibroblasts, where the transcription factor Forkhead Box M1 (FOXM1) is involved.^146,147^ In nasopharyngeal carcinoma cells, Epstein-Barr virus (EBV) latent protein Latent Membrane Protein 1 (LMP1) induces dedifferentiation to form stem-like cells through transcriptional inhibition of CCAAT Enhancer Binding Protein Alpha (CEBPA).^148^ Exposure to progranulin leads to dedifferentiation of breast cancer cells and expansion of the CSC population.^149^ Exosomes from GSCs can cause dedifferentiation of surrounding non-CSCs by activating the Notch1 pathway.^150^ Finally, by stabilizing OCT4, a crucial transcription factor in CSCs, NO can induce the formation of lung or endometrial CSCs from differentiated cells.^151,152^

#### Non-malignant stem/progenitor cells

CSCs are generally functionally and structurally like normal stem cells, such as the ability of self-renewal and multipotent differentiation and similar transcriptional profiles.^128^ For instance, prostate CSCs share a conserved transcriptional program with normal prostate basal stem cells.^153^ Also, based on results from immunohistochemistry and double-fluorescence immunostaining, hepatocellular cholangiocarcinoma shares a similar set of markers with hepatic progenitor cells.^154,155^ These observations indirectly support that these CSCs can derive from tissue-resident stem/progenitor cells (Fig. 2).

Several studies have succeeded in the transformation from induced pluripotent stem cells (iPSCs) to CSCs. When cultured in a conditioned medium of mouse Lewis lung cancer,^156,157^ pancreatic carcinomas,^158,159^ hepatocellular carcinomas,^160^ or prostate cancer cell lines,^161^ iPSCs obtained CSC features and higher tumorigenicity in vivo. Similar results can be obtained by culturing iPSCs with Lewis cell-derived extracellular vesicles^162^ or recombinant human Fibroblast Growth Factor 2 (FGF2).^163^ Moreover, mouse embryonic stem cells can also get converted into CSCs in conditioned medium from mouse Lewis lung cancer or melanoma cells.^164^ These studies provide evidence that CSCs can be induced from normal stem cells, although iPSCs are not equivalent to normal somatic stem cells. *Ewing Sarcoma Breakpoint Region 1 (EWS)-Friend Leukemia Integration 1 (FLI-1)* fusion gene and miR-145 in human pediatric mesenchymal stem cells drive their reprogramming into CSCs by increasing the expression of SOX2.^165^ Although iPSCs do not fully represent adult stem cells, these studies show a possibility that CSCs can be induced from normal stem cells.

Some studies give more direct evidence that CSCs may originate from non-malignant adult stem cells. For instance, following *Adenomatous Polyposis Coli (Apc)* depletion, LGR5^+^ intestinal stem cells transform into CSCs, fueling the unimpeded growth of adenomas.^166^ Hepatocellular CSCs are found to derive from hepatic progenitor cells when the TGF-β or the WNT pathway is constantly activated in mice.^167,168^ Mouse primary hepatic stem/progenitor cells, when transduced with oncogenic genes, acquire CSC markers, self-renewal ability, and pluripotency.^169,170^ It is also notable that lineage-committed hepatoblasts and differentiated adult hepatocytes also gain stemness after the process.^169^ Finally, following deletion of *Brca1*, mouse mammary epithelial luminal progenitors get the ability to generate basal-like breast tumors.^171^

#### Cell-cell fusion

Cell-cell fusion is commonly involved in several physiological processes, including fertilization, muscle maturation, development of bones and placenta, and immune responses.^172,173^ For instance, a sperm and an egg fuse into a fertilized egg, a set of mononucleated myoblasts form a string of muscle fibers, trophoblasts fuse to form syncytiotrophoblasts, and several macrophages combine to make giant cells.^172,173^ Bone marrow cells can adopt the phenotype of other cells, such as embryonic stem cells, through cell-cell fusion.^174^ Similarly, cancer cells can also fuse with other cancer cells or non-malignant cells, forming tumor hybrid cells.^175,176^ Particularly, fusion of cancer cells with non-malignant cells often gives rise to their malignancy and potentiates tumor heterogeneity.^172^ For instance, melanoma cells can gain phenotypes of fibroblasts and monocytes by cell-cell fusion,^177^ and co-grafting of bone marrow-derived mesenchymal/stromal stem cells (BM-MSCs) and murine prostate cancer cells in vivo leads to enhanced tumor growth by cell-cell fusion.^178^ Clinically, a study suggests that the number of tumor hybrids (fusion of cancer cells and leukocytes) in peripheral blood correlates with cancer stage and patients’ survival.^179^

Given these properties of tumor hybrid cells, several studies support that cell-cell fusion can be one of the origins of CSCs (Fig. 2). MSCs have been recognized as an essential component in the tumor microenvironment and actively participate in tumor progression.^180^ Fusion between BM-MSCs or embryonic stem cells and cell lines of breast cancer, NSCLC, liver cancer, ovarian cancer, or gastric cancer upregulated their stem cell markers and enhanced tumorigenicity abilities in vitro.^181–186^ Similarly, hybrids from human/mice liver cancer, breast cancer, or lung cancer cells and BM-MSCs exhibited mesenchymal features and demonstrated enhanced stemness and metastatic capabilities in vivo.^187–189^ Besides solid tumors, cells of hematological malignancies, such as multiple myeloma, can also fuse with BM-MSC to gain stemness and stronger resistance to treatments.^190^ However, the fusion of BM-MSCs with cancer cells does not always produce CSCs, such as esophageal CSCs,^191^ indicating that cell fusion is not the only mechanism of CSC formation at least in certain tumor types. Plus, human umbilical cord MSCs can also fuse with gastric cancer cells to enhance cancer proliferation, migration, and stemness.^192^

In addition to MSCs, cancer cells can also fuse with other types of non-malignant cells and gain stem-like properties in the process. CD34^+^ liver CSCs can be formed by fusion of hepatobiliary stem/progenitor cells with CD34^+^ hematopoietic precursor-derived cells,^193^ suggesting that tissue-resident stem cells are also able to fuse with other cells and form CSCs. The fusion of prostate cancer cells with muscle cells increased the number of CD133^+^ stem-like cells.^194^ Hybrids from fusions of non-malignant human breast epithelial cells and breast cancer cells exhibit CSC properties,^195^ which is dependent on the transcription factor Zinc Finger E-Box Binding Homeobox 1 (ZEB1).^196^ Hybrids from tumor-associated macrophages and breast cancer cells also exhibit CSC phenotype and promote cancer metastases in a mouse model.^197^ Furthermore, CSCs can also fuse with other cells and obtain higher malignancy. For instance, hybrids of CSCs and monocytes gain highly invasive capacities,^198^ and those of BM-MSCs and SU3-RFP human glioma stem cells (GSCs) exhibited enhanced angiogenic effects compared to the parental cells.^199^

Additionally, a study shows that the fusion of two human lung fibroblast cell lines, E6E7 and RST, results in hybrids with elevated ALDH activity, which is a CSC marker.^200^ This study suggests that hybrids from two non-malignant cells may also lead to CSC formation.

#### Environmental factors in cancer stem cell formation and/or maintenance

It is commonly believed that CSCs reside in niches of tumors, and their microenvironment, which is generally characterized by hypoxia, aberrant angiogenesis, and chronic inflammation, has great impacts on the formation and maintenance of CSCs (Fig. 2).^200^

#### Hypoxia/angiogenesis

Hypoxia and aberrant angiogenesis have been identified as two crucial features of the tumor microenvironment.^201,202^ Tumor angiogenesis can be a consequence of hypoxia since hypoxia serves as a potent stimulus of Vascular Endothelial Growth Factor (VEGF) production, and disorganized vessels in tumors can aggravate hypoxia and vice versa.^203^ Under hypoxia, the HIF system is activated.^204^ And upregulated HIFs can promote the dedifferentiation of pancreatic cancer cells by activating the Notch pathway^142^ or that of melanoma cells by upregulating OCT4 expression.^205^ Also, hypoxia induces upregulation of SOX2, OCT4, KLF-4, Nanog, and Lin-28A, which are transcription factors contributing to dedifferentiation, and formation of stem-like cells in glioma, lung cancer, and hepatoma cells.^145^ Likewise, VEGF, an essential pro-angiogenic molecule, can interact with the VEGFR family or the neuropilin (NRP) family and promote stemness of skin/breast cancer cells and extend the CSC pool in the niche.^206,207^ Hypoxia can also indirectly regulate the stemness of cancer cells by altering functions of surrounding stromal cells, such as cancer-associated fibroblasts (CAFs)^208,209^ and myeloid-derived suppressor cells (MDSCs).^210^

#### Cancer-associated fibroblasts

CAFs are a group of interstitial cells of a mesenchymal lineage that are not epithelial, endothelial, or immune cells found in or adjacent to tumors.^210^ Compared to normal fibroblasts, CAFs are hyperproliferative and have a unique secretion pattern that contributes to tumor angiogenesis and metastases.^211^ Some believe that CAFs can also promote dedifferentiation and CSC formation by activating the WNT or the Notch pathway.^4^ WNT5a from surrounding CAFs induce dedifferentiation of ovarian cancer and gastric cancer cells and maintain the undifferentiated state of ovarian CSCs by activating a noncanonical WNT pathway.^212,213^

Moreover, several studies show that secretomes from CAFs promote the stemness of cancer cells. Head and neck squamous cell carcinoma and scirrhous gastric cancer cells express higher CSC markers when cultured in a CAF-derived conditioned medium compared to that from normal fibroblasts.^214,215^ CAFs secret IL-6, activin-A, G-CSF, and IGF-II that mediate the dedifferentiation of lung cancer cells into CSCs.^144,146^ Periostin from podoplanin-positive CAFs facilitates stem-like properties of gastric cancer cells by activating the Focal Adhesion Kinase (FAK)/Yes-Associated Protein (YAP) signaling.^216^ Leukemia Inhibitory Factor (LIF) and Gremlin 1 from CAFs can promote Nanog and OCT4 expression along with stem cell markers CD24^−^/CD44^+^ in breast cancer cells.^217,218^ CAF-derived HGF and IL-6 enhance the stemness of CD24^+^ liver cancer cells by activating the STAT3 pathway,^219^ and IL-6 from CAFs also induce Chromobox 4 (CBX4) expression, which is a CSC phenotype regulator, in skin squamous cell carcinoma.^220^ Additionally, Matrix Metallopeptidases (MMPs) from activated CAFs induce EMT and enhance the stemness of prostate cancer cells.^221^ However, when liver cancer or pancreatic ductal adenocarcinoma cell lines are cultured in a conditioned medium from CAFs, they have distinct expressions of CSC markers and aggressive phenotypes in a cell-line dependent manner,^222,223^ suggesting that effects of CAFs on stemness of cancer cells may vary depending on types and subtypes of cancers.

Moreover, CAF-derived exosomes also participate in CSC formation and/or maintenance. MiR-146a-5p in CAF-derived exosomes can promote the stemness of bladder cancer cells by activating the Mammalian Target Of Rapamycin (mTOR) pathway.^224^ CircHIF1A in exosomes from hypoxic CAFs can sponge miR-580-5p in breast cancer cells and increase their expression of CD44, which is a CSC marker for breast cancers.^208^ And small extracellular vesicles with low level of miR-7641 are associated with activation of the HIF-1α pathway, which promotes stemness of breast cancer cells.^225^ Likewise, loss of miR-34c-5p in exosomes from CAFs maintains the stemness of laryngeal cancer cells.^226^ In summary, CAFs regulate the stemness of cancer cells through paracrine mechanisms that involve cytokines or extracellular vesicles.

#### Mesenchymal stem cells

During tumor initiation and development, MSCs are believed to be constantly recruited to the tumor, making them an unneglectable group of cells in the tumor microenvironment (TME).^227^ Coculturing BM-MSCs with hypopharyngeal or prostate cancer cells induces expression of stemness markers in these cells,^228,229^ indicating MSCs can be not only the origin of CSCs but also an ally in CSC formation and maintenance. MSCs can induce fatty acid oxidation by upregulating mitofusin 2, a mitochondrial fusion-inducible factor, Carnitine Palmitoyl Transferase 1 (CPT1), and lncRNA MACC1-AS1 in gastric cancer, which finally leads to enhancement of stemness.^230–232^ Plus, the direct contact between MSCs and breast cancer cells upregulates the miR-199a in cancer cells, which subsequently represses the transcriptional regulator forkhead box P2 (FOXP2) and finally leads to higher stemness.^233^

Culturing colon cancer or melanoma cells in an MSC-derived conditioned medium increased the expressions of stemness markers of the cancer cells,^234,235^ indicating the secretomes of MSCs can induce stemness of cancer cells. Platelet-Derived Growth Factor (PDGF) from MSCs gives rise to ALDH^+^ CSCs in a model of ovarian malignant ascites.^236^ MSC-derived IL-8 can induce stem-like properties of gastric cells and blocking Programmed Cell Death-Ligand 1 (PD-L1) undermines this effect by reducing the expression of the transcription factor CTCF.^237^ Conditioned medium or just IL-6 from MSCs increases expression of stemness markers, such as OCT4, Nanog, and SOX2, via NF-κB activation in osteosarcoma cells.^238,239^ Prostaglandin E2 from MSCs also increases the level of ALDH-high CSCs in human colorectal carcinoma cells by activating the β-Catenin pathway.^240^ Exosomes from p53 deficient mouse BM-MSCs can internalize UBR2 into gastric cancer cells and increase their expression of CSC markers via the WNT/β-Catenin pathway.^241^ Adipose- and placenta-derived MSCs increase the proportion of CD133^+^/CD44^+^ colon CSCs via the IL-8/Mitogen-Activated Protein Kinase (MAPK) pathway.^242^

However, MSCs do not always facilitate the stemness of cancer cells. For instance, endometrium‑derived MSCs suppress the stemness of endometrial cancer by inhibiting the WNT/β‑Catenin signaling pathway.^243^ MSC-derived exosomes reduce the proliferation, migration, invasion, angiogenesis-stimulating, and self-renewal abilities of hepatocellular CSCs by inducing ERK phosphorylation^244^ or pancreatic CSCs by inhibiting the β-Catenin signaling.^245^ Altogether, MSCs are an important component in the TME, some of which can promote the stemness of their surrounding cancer cells through their secretomes or exosomes, while some MSCs may reduce the stemness of the surrounding cells.

#### Macrophages

Tumor-associated macrophages (TAMs) represent one of the most abundant groups of immune cells in tumors.^246^ Based on their immune functions, TAMs can be simply classified into the M1 proinflammatory phenotype and the M2 anti-inflammatory phenotype, although this classification neglects the great diversity of TAMs.^246^ ScRNA-seq shows that the maintenance of stemness of hepatocellular cells is mainly based on M2 polarization rather than the recruitment of TAMs.^247^ In the spleen of a murine chronic myeloid leukemia model, red pulp macrophages provide a niche for leukemia stem cells and support their stemness.^248^

Coculturing of TAMs and pancreatic cancer cells or culturing oral squamous cell carcinoma in an M2 macrophage-derived conditioned medium promotes their expression of stemness-related genes.^249,250^ Conditioned medium from TAMs promotes stemness of lung cancer cells by upregulating Ubiquitin-Specific Peptidase 17 (USP17), which subsequently disrupts the TNFR-Associated Factor (TRAF) 2/TRAF3 complex.^251^ These results suggest that the secretomes of TAM are essential in the process. TAM-derived TGF-β1 promotes stem-like properties of esophageal squamous cancer cells,^252^ glioblastoma cells,^253,254^ pancreatic cancer cells,^255^ prostate cancer cells,^256^ hepatocellular cancer cells,^257^ and breast cancer cells.^258^ Additionally, M2-TAMs secretory pleiotrophin enlarges the CSC group in human diffuse large B lymphoma by upregulating the β-Catenin expression.^259^ TAM-derived interleukin-1β, TNF-α, and IL-6 promote stemness of Doublecortin Like Kinase 1 (Dclk^+^) colon tuft cells and initiate tumor growth.^260^ TAM-derived IL-6 also enriches breast CSCs by activating the STAT3 signaling,^261^ and it also activates WNT and promotes stemness of ovarian cancer cells in 3D engineered microenvironments.^262^ M2-TAM-derived IL-8 induces stemness of ovarian cancer cells in vitro by activating the STAT3 pathway.^263^ Macrophages can also secrete IL-10 to promote stemness of NSCLC cells by activating the JAK1/STAT1/NF-κB/Notch1 signaling.^264^ IL-33 can also recruit macrophages into the TME and stimulate the secretion of prostaglandin E2, which subsequently supports stemness of colon cancer cells.^265^ Inhibitor Of Differentiation 1 (ID1) from TAMs can inhibit transcription of two stemness inhibitory factors, SerpinB2 and CCL4, and lead to stemness enhancement.^266^ Besides, LSEC in TAMs can enhance breast cancer stemness by binding to Butyrophilin Subfamily 3 Member A3 (BTN3A3) on breast cancer cells,^267^ suggesting that direct cellular contact of TAMs and cancer cells can also enhance stemness. TAM secretory S100 calcium-binding protein can induce stemness of hepatocellular cancer cells by activating the NF-κB pathway in a calcium-dependent manner.^268^ M2-TAM secretory VEGF or EGF promotes stemness of breast cancer cells by activating the VEGF/NRP-1/GTPase Activating Protein And VPS9 Domains 1 (GAPVD1) axis or the EGFR/STAT3/SOX2 signaling, respectively.^207,269^ M2-TAM-derived IGF-1 and IGF-2 promotes thyroid cancer stemness by activating the PI3K/AKT/mTOR pathway.^270^ Macrophage-derived glycoprotein nonmetastatic B induces the production of IL-33, an IL-1-like cytokine, via CD44 in a mouse lung cancer model, which in turn induces the CSC properties of these cells.^271^ Likewise, in head and neck squamous cell carcinoma, macrophage-derived hyaluronic acid (HA) induces activation of the PI3K/Eukaryotic Translation Initiation Factor 4E-Binding Protein 1 (EIF4EBP1)/SOX2 signaling via CD44 and increases the density of CSCs in vitro.^272^ M2-TAM-derived exosomal miR-27a-3p and the miR-17-92 cluster promote stemness of hepatocellular cancer cells by upregulating Thioredoxin-Interacting Protein (TXNIP) or disturbing the balance of the TGF-β1/Bone Morphogenetic Protein 7 (BMP-7) pathways.^273^

Several chemokines and chemokine ligands are involved in TAM-induced CSC formation or maintenance as well. TAM-derived Chemokine C-C Motif Ligand 2 (CCL2) activates AKT and increases the expression of β-Catenin in triple-negative breast cancer cell lines, which eventually induces their CSC properties.^274^ CCL8 promotes stemness of glioblastoma cells by activating ERK1/2.^275^ CXCL12 and TGF-β from M2 TAMs elevate DNA Topoisomerase II Alpha (TOP2A) expression and enhance stemness of hepatocellular cancer cells via the TOP2A/β-Catenin/YAP1 axis.^276^ Also, CXCL12 from M2 macrophages activate the WNT/β-Catenin pathway to facilitate stemness of colorectal cancer cells.^277^ Macrophage-derived CXCL7 fosters glioma stemness.^278^ Macrophage secretory IL-1β and CCL18 facilitate stemness of head and neck squamous carcinoma.^279,280^

Notably, M1 macrophages can also induce a subgroup of CD44^high^/CD24^−/low^ or ALDH1^+^ breast CSCs in vitro, although prolonged coculture finally endows the macrophages with M2 properties.^281^ A study shows that breast CSCs respond more robustly to monocytes/macrophages than do differentiated non-stem cells through a juxtracrine mechanism, indicating monocytes/macrophages play an essential role in maintaining CSC niches.^282^

#### Myeloid-derived suppressor cells

MDSCs are a heterogeneous group of immune cells from the myeloid lineage that exert immunosuppressive effects.^283^ Coculture of ovarian cancer cells and MDSCs increases the expression of colony-stimulating factor 2 that activates the STAT3 and leads to upregulation of stemness markers.^284^ MDSCs can upregulate the expression of miR101 in ovarian cancer cells and subsequently repress the core-pressor gene *C-terminal Binding Protein-2 (CtBP2)*, which restrains cancer stemness.^285^ Granulocytic MDSCs trigger piRNA-823 expression that promotes DNA methylation and maintains the stemness of multiple myeloma CSCs.^286^ Hypoxia can induce increased secretion of exosomes containing S100 Calcium-Binding Protein A9 (S100A9) from granulocytic MDSCs, which leads to enhanced stemness of colorectal cancer cells.^210^ MDSCs cultured in CAF-derived conditioned medium express a higher level of 5-Lipoxygenase (5-LO) that induces synthesis of leukotriene B4, which finally results in enhanced stemness of intrahepatic cholangiocarcinoma.^287^ MDSCs also endow stemness to breast cancer cells by secretory IL-6 and NO that activate the STAT3 and Notch pathways, respectively.^288^ The Notch pathway can also be activated by granulocytic MDSCs and contributes to stem maintenance in esophageal squamous cell carcinoma,^289^ and the STAT3 pathway is also activated in pancreatic cancer cells in the presence of MDSCs.^290^ MDSC-derived PGE2 also increases the stem cell-like properties in epithelial ovarian cancer.^291^

Besides, NO is frequently upregulated in cancers.^292^ NO disturbs the ubiquitin-mediated prosomal degradation of OCT4 and induces dedifferentiation of human lung cancer cells.^151^ NO also promotes stem-like properties of mouse glioma cells by activating the Notch pathway.^293^ Ionizing radiation is one of the inducers of CSCs’ formation across several cancer types,^294^ which will be discussed in more detail in the CSCs and sensitivity/resistance to radiotherapy section.

### Features of cancer stem cells

#### Self-renewal and pluripotency

Since the first identification of CSCs in 1997,^2^ self-renewal and pluripotency have been considered two essential features of CSCs. This discovery comes from the observation that ALL cells are organized hierarchically, with a subset of cells that can replicate themselves and give rise to other malignant lineages, mimicking normal hematopoietic stem cells.^2^ In solid tumors, CSCs were first identified in breast cancer, in which the CD44^+^ CD24^−/low^ lineage-cells underwent self-renewal and differentiation processes.^23^ These two properties of CSCs are also the basis to explain the formation of intratumoral heterogeneity in the CSC hypothesis or the hierarchical model of tumorigenesis^4^.

#### Cancer stem cells in cancer development

Based on the hierarchical model of carcinogenesis or the classical CSC hypothesis, CSCs, originating from normal stem cells or progenitors, are the cellular origins of cancers that can self-renew and give rise to the cellular hierarchies that explain the intratumoral heterogeneities.^295^ Nevertheless, the observation that some cancer cells can interchange between differentiated states and stem-like states does not favor this hypothesis.^296^ Plus, some cancers do not follow the CSC model.^297^ Therefore, although the terms tumor-initiating cell (TIC) and CSC have been used interchangeably, CSCs are not necessarily the cell origin of cancers based on the cellular plasticity model.^4^ That is, some malignant differentiated cells with oncogenic mutations can undergo dedifferentiation and form stem-like cells that cause intratumoral heterogeneities.^4^ However, this does not eliminate the role of CSCs in cancer initiation supported by many studies. AML cells originate from a subgroup of stem-like cells, as mentioned above.^2^ LGR5^+^ intestinal crypt stem cells, upon oncogenic mutations, serve as cells of origin of intestinal cancer.^166^ Injection of CD133^+^ human brain CSCs into non-obese diabetic, severe combined immunodeficient mice causes tumor formation, while that of CD133^−^ does not.^24^ Conversely, deletion of SOX2, which is essential in maintaining the stemness of CSCs, decreases the formation of skin squamous-cell carcinoma.^298^ Also, decreasing MYC activity that sustains stemness of hepatocellular CSCs attenuates hepatocellular carcinoma initiation.^299^

CSCs are generally characterized by vigorous proliferation.^3^ Cancer proliferation is heavily dependent on the activation of the AKT, mTOR, and MAPK/ERK, which result in upregulated expression of proteins responsible for the cell cycle.^300^ The signaling pathways that involve these molecules are also major signaling pathways,^5^ which we will introduce in detail in the following section. Indeed, the acquisition of stemness is usually accompanied by enhancement of proliferation.^301–304^ Conversely, interventions that inhibit stemness also impair the proliferative potential of the cells.^305,306^

Cancer metastasis involves several biological processes that can be summarized into 5 essential steps, including cell escape, intravasation, survival maintenance, extravasation, and outgrowth.^307^ In epithelial malignancies, the EMT is a crucial event in metastasis.^308^ And the EMT can generate stem-like cells in human mammary epithelial cells,^66^ indicating a strong correlation between the EMT and CSCs. Molecularly, the WNT/β-Catenin, Notch, PI3K/AKT, hedgehog, and NF-κB signaling pathways are involved in the acquisition of mesenchymal properties of cancer cells. The pathways are also crucial in inducing and maintaining the stemness of CSCs.^309^

CSCs are often indicated as a reason for multi-drug resistance. This is partially attributed to their capability to maintain quiescence to avoid the therapeutic effects of anti-cancer treatments.^310^ For instance, CD13^+^ hepatocellular CSCs predominate in the G0 phase of the cell cycle and exhibit resistance to 5-fluorouracil treatment, as the mechanism of 5-fluorouracil primarily involves inhibition of DNA replication.^311^ In addition, CSCs can reduce intracellular accumulation of therapeutic agents by overexpressing ALDH and ATP-Binding Cassette (ABC) transporters.^312^ They also have better DNA repair capabilities and ROS clearance to avoid apoptosis induced by chemotherapy or radiation therapy stress.^313^ Finally, CSC supports an immunosuppressive niche that can exclude therapeutic agents and impair the efficacy of immunotherapy.^4^ Detailed mechanisms for CSC-induced chemoresistance, radioresistance, and resistance to targeted therapy and immunotherapy will be introduced in the following sections.

### Biomarkers of cancer stem cells

One of the most efficient ways to identify CSCs in tumors is to use biomarkers for CSCs. Based on their cellular distribution, CSC markers can be classified into intracellular markers and cell-surface markers. Intracellular markers include transcription factors that function in the nucleus and markers found in the cytoplasm. Tables 2 and 3 summarize frequently used CSC markers in solid tumors and hematopoietic malignancies, respectively. Among them, generally accepted markers are introduced below.

**Table 2:** Frequently used cancer stem cell markers for solid tumors

Biomarker	Full name and alternative name(s)	Expression in cancer types	Function(s)
Cell surface markers			
CXCR4	C-X-C Chemokine Receptor Type 4 Fusin CD184	Pancreatic cancer^1042^ Gastric cancer^1043^ Breast cancer^1044–1048^ Colorectal cancer^1049^ Esophageal cancer^1050^ Lung cancer^1051,1052^ Glioma/glioblastoma^1053,1054^ Renal cell carcinoma^1055^	A chemokine receptor that contributes to HIV infection and triggers activation of several signaling pathways that supports cell proliferation, migration, and survival^355^
LGR5	Leucine Rich Repeat Containing G Protein-Coupled Receptor 5 G-Protein Coupled Receptor 49 (GPR49) G-Protein Coupled Receptor 67 (GPR67)	Gastric cancer^1056,1057^ Glioma/glioblastoma^1058^ Cervical cancer^1059^ Colorectal cancer^132,1060^ Hepatocellular cancer^1061^ Pancreatic cancer^1062^	A member of the WNT signaling pathway
EpCAM	Epithelial Cell Adhesion Molecule CD326	Hepatocellular cancer^1063^ Head and neck squamous cell carcinoma^1064^ Breast cancer^1065,1066^	Homotypic cell adhesion Epithelial mesenchymal transition
ProC-R	Protein C Receptor (PROCR) Endothelial Protein C Receptor (EPCR) Activated Protein C Receptor (APC receptor) CD201	Head and neck squamous cell carcinoma^1067^ Breast cancer^1068,1069^	Enhancing activation of protein C
LINGO2	Leucine-Rich Repeat And Immunoglobulin-Like Domain-Containing Nogo Receptor-Interacting Protein 2	Gastric cancer^1070^	Suppressing EGFR phosphorylation^1071^
CD24	Heat Stable Antigen (HSA)	Hepatocellular cancer^219^ Prostate cancer^1072^ Head and neck squamous cell carcinoma^1073,1074^ Colorectal cancer^1075,1076^ Gastric cancer^1077^ Bladder cancer^1078^	Mediating the WNT/β-Catenin, MAPK, PI3K/AKT/mTOR, Notch, and hedgehog pathways^361^
CD44^+^/CD24^+^		Cervical cancer^1079^ Pancreatic cancer^1080^	
CD44^+^/CD24^−^		Breast cancer^1081,1082^ Prostate cancer^1083^ Head and neck squamous cell carcinoma^1084,1085^ Ovarian cancer^1086^	
CD44	Homing Cell Adhesion Molecule (HCAM) Phagocytic Glycoprotein-1 (Pgp-1)	Cervial cancer^1087^ Pancreatic cancer^271,1088^ Melanoma^1089^ Lung cancer^1090,1091^ Colorectal cancer^1092,1093^ Head and neck squamous cell carcinoma^1094,1095^ Glioma/glioblastoma^1096,1097^ Bladder cancer^1098^ Breast cancer^926,1099^ Prostate cancer^1100^ Ovarian cancer^1101^	Recruiting ezrin/radixin/moesin (ERM) proteins to interact with VEGFR and to actiavte the PI3K/Akt and Src/MAPK pathways Co-receptor of c-Met^362^
CD133	Prominin-1 PROM1	Colorectal cancer^366,1102^ Lung cancer^1103^ Glioma/glioblastoma^1104,1105^ Esophageal cancer^1106^ Hepatocellular cancer^380,1107^ Gastric cancer^1108^ Melanoma^1109^ Cervical cancer^1110^ Breast cancer^1111^ Pancreatic cancer^1112^ Prostate cancer^1113^	A member of pentaspan transmembrane glycoproteinsActivating the PI3K/AKT, Src, and β-Catenin^363^
CD24^+^/CD133^+^		Hepatocellular cancer^1114^	
CD44^+^/CD133^+^		Gallbladder cancer^364^	
CD44^+^/CD133^−^		Colorectal cancer^365^	
CD44^−^/CD133^+^		Colorectal cancer^366^	
CD166	Activated Leukocyte Cell Adhesion Molecule (ALCAM) CD6 Ligand (CD6L)	Colorectal cancer^1115^ Head and neck squamous cell carcinoma^1116^	A cell–cell adhesion molecule
CD87	Urokinase Plasminogen Activator Surface Receptor (uPAR)	Lung cancer^1117^ Glioma^1118^ Medulloblastoma^1119^	A part of the plasminogen activation system
CD90	Thy-1	Pancreatic cancer^1120,1121^ Breast cancer^1122^ Hepatocellular cancer^1123^	Src activation^1124^
CD29	Integrin β1 ITGB1	Endometrial cancer^1125^ Breast cancer^1126^ Lung cancer^1127^ Head and neck squamous cell carcinoma^869,1128^	A member of the integrin family
CD61	Integrin β3 ITGB3	Breast cancer^1129^ Lung cancer^1129^ Pancreatic cancer^1129^ Breast cancer^1130^	A member of the integrin family
CD70		Glioblastoma^1131^ Breast cancer^1132^	A costimulatory molecule
CD49f	Integrin α6	Breast cancer^1133,1134^ Glioma/clioblastoma^1135,1136^ Epidermal squamous cell carcinoma^1137^	A member of the integrin family
Intracellular markers			
SOX2		Breast cancer^1138,1139^ Colorectal cancer^512,1140^ Lung cancer^1141^ Glioma/glioblastoma^1142,1143^ Pancreatic cancer^1144,1145^ Retinoblastoma^1146^ Skin squamous-cell carcinoma^298^ Head and neck squamous cell carcinoma^1147,1148^ Esophageal cancer^1149,1150^ Renal cell carcinoma^1151^ Cervical cancer^1152^ Pleural mesothelioma^1153^ Gastric cancer^1154^ Hepatocellular cancer^1155^ Osteosarcoma^1156,1157^ Ovarian cancer^1158^ Neuroblastoma^1159^ Bladder cancer^1160,1161^ Melanoma^1162^ Sarcoma^165^	A transcription factor that maintains self-renewal and pluripotency of stem cells
OCT4	Octamer-Binding Transcription Factor-4 POU Domain, Class 5, Transcription Factor 1 (POU5F1)	Endometrial cacner^152^ Glioma/glioblastoma^1163^ Breast cacner^1164^ Head and neck squamous cell cancer^1165^ Gastric cancer^1166^	A homeodomain transcription factor of the POU family that maintains self-renewal of stem cells
Nanog	Nanog Homeobox	Breast cancer^1167,1168^ Lung cancer^1169,1170^ Hepatocellular cancer^1171,1172^ Colorectal cancer^1173,1174^ Ovarian cancer^1175,1176^ Esophageal cancer^1177^ Gastric cancer^1178^ Prostate cancer^1179^ Renal cell carcinoma^1180^ Pancreatic cancer^1062^ Head and neck squamous cell carcinoma^1181^ Glioma/glioblastoma^1182^ Cervical cancer^1183^	A transcription factor that maintains pluripotency of stem cells
SALL4	Sal-Like Protein 4	Hepatocellular cancer^1184,1185^ Melanoma^1186^ Choriocarcinoma^1187^ Esophageal cancer^1150^ Breast cancer^1099^ Ovarian cancer^1188^	A transcription factor that maintains pluripotency of stem cells^1189^
ALDH	Aldehyde Dehydrogenase	Colorectal cancer^1190^ Head and neck squamous cell carcinoma^1191^ Lung cancer^1192^ Glioma/glioblastoma^1193^ Endometrial cancer^1194^ Neuroblastoma^1195^ Ovarian cancer^1101^ Renal cell carcinoma^1196^ Adenoid cyst carcinoma^1197^ Breast cancer^1198^ Cholangiocarcinoma^1199^	A polymorphic enzyme that oxidates aldehydes to carboxylic acids
Musashi-1/2	RNA-Binding Protein Musashi Homolog 1/2	Hepatocellular carcinoma^894^ Endometrial cancer^829^ Glioblastoma^1200^ Breast cancer^1201^ Colorectal cancer^1202^ Esophageal cancer^1203^ Lung cancer^1204^	RNA-binding protein involved in post-transcriptional mRNA editing
Letm1	Leucine Zipper-EF-Hand Containing Transmembrane Protein 1	Gastric cancer^339^ Colorectal cancer^338^ Lung cancer^337^ Osteosarcoma^341^	A Ca^2+^/H^+^ antiporter in the inner membrane of mitochondria^336^
AFP	Alpha Fetoprotein	Pancreatic cancer^343^ Cholangiocarcinoma^344^ Hepatocellular cancer^345^	A carrier protein in the fetus liver and the yolk sac
BMI-1	B Lymphoma Mo-Mlv Insertion Region 1 Homolog Polycomb Group RING Finger Protein 4 (PCGF4) RING Finger Protein 51 (RNF51)	Gastric cancer^1205^ Hepatocellular cancer^1206^ Endometrial cancer^1207^ Thyroid cancer^1208^ Lung cancer^1209^ Head and neck squamous cell carcinoma^1210^ Colorectal cancer^1211^ Glioma/glioblastoma^1212^ Pancreatic cancer^1213^ Prostate cancer^1209^	A subunit of PRC1 Negatively regulating p16^INK4a^ and p14^ARF^/p19^ARF^ expression at the transcriptional level Inhibiting E-cadherin expression^1214^
Dcamkl-1	Doublecortin-Like Kinase 1	Colorectal cancer^351^	A microtubule-associated protein that also mediates inflammation response^350^

**Table 3:** Frequently used cancer stem cell markers for hematopoietic malignancies

Biomarker	Other name(s)	Expression in cancer types	Function
Cell surface markers			
IL1RAP	Interleukin 1 Receptor Accessory Protein IL1R3	Chronic myeloid leukemia^369^ Acute myeloid leukemia^368^	A receptor for interleukin-1
CD133	Prominin-1 PROM1	Acute lymphoblastic leukemia^1215^ Acute myeloid leukemia^1216^	Activating the PI3K/AKT, Src, and β-Catenin^363^
CD70/CD27		Acute myeloid leukemia^374^ Chronic myeloid leukemia^375^	CD70: a costimulatory molecule CD27: a costimulatory molecule on T cells
CD34^+^/CD38^−^		Chronic myeloid leukemia^1217^ Acute myeloid leukemia^1218^	CD34: a member of a family of single-pass transmembrane sialomucin proteins and an adhesion molecule CD38: a receptor for CD31 and an enzyme that catalyzes the synthesis of ADP ribose and cyclic ADP-ribose^1219^
CD25	Interleukin-2 Receptor Alpha Chain (IL2RA)	Acute myeloid leukemia^370^ Chronic myeloid leukemia^372^	A receptor for interleukin-2
CD123	Interleukin-3 Receptor	Acute myeloid leukemia^373^	A receptor for interleukin-3
CD26	Dipeptidyl Peptidase-4 (DPP4 or DPPIV) Adenosine Deaminase Complexing Protein 2 (ADCP2)	Chronic myeloid leukemia^1220^	A cell-surface enzyme that cleave a wide range of peptides^1221^
CD36	Platelet Glycoprotein 4 Fatty Acid Translocase (FAT) Scavenger Receptor Class B Member 3 (SCARB3) Glycoproteins 88 (GP88)	Chronic myeloid leukemia^1222^	A member of the class B scavenger receptor family
CD90	Thy-1	Acute lymphoblastic leukemia^377^	Src activation^1124^
CD110	Thrombopoietin Receptor Myeloproliferative Leukemia Protein	Acute lymphoblastic leukemia^377^	A receptor for thrombopoietin
CD371	CLEC12A CLL-1	Acute myeloid leukemia^1223^	A member of the C-type lectin/C-type lectin-like domain (CTL/CTLD) superfamily
TIM-3	Hepatitis A Virus Cellular Receptor 2 (HAVCR2)	Acute myeloid leukemia^1224^	An immune checkpoint on lyphocytes, myeloid cells, and other cells
CD117	KIT Proto-Oncogene c-KIT Tyrosine-Protein Kinase KIT Mast/Stem Cell Growth Factor Receptor (SCFR)	Acute myeloid leukemia^1225^	A receptor tyrosine kinase involved in hematopoiesis and gametogenesis
Intracellular markers			
SOX2	SRY-Box Transcription Factor 2	T-cell leukemia^1226^	A transcription factor that maintains self-renewal and pluripotency of stem cells
ALDH	Aldehyde Dehydrogenase	Chronic myeloid leukemia^1227^ Acute myeloid leukemia^1228^ Multiple myeloma^1229^	A polymorphic enzyme that oxidates aldehydes to carboxylic acids
Nanog	Nanog Homeobox	T-cell leukemia^1226^ Acute myeloid leukemia^1230^	A transcription factor that maintains pluripotency of stem cells
Musashi-2	Musashi RNA Binding Protein-2	Mixed-lineage leukemia^334^ Mantle cell lymphoma^335^ Myeloid leukemia^333^	RNA-binding protein involved in post-transcriptional mRNA editing
OCT3/4	Octamer-Binding Transcription Factor-3/4 POU Domain, Class 5, Transcription Factor 1 (POU5F1)	Acute myeloid leukemia^1231^	A homeodomain transcription factor of the POU family that maintains self-renewal of stem cells
SALL4	Sal-Like Protein 4	Mixed lineage leukemia^1232^ Chronic myelogenous leukemia^1233^	A transcription factor that maintains pluripotency of stem cells^1189^
BMI-1	B Lymphoma Mo-Mlv Insertion Region 1 Homolog Polycomb Group RING Finger Protein 4 (PCGF4) RING Finger Protein 51 (RNF51)	Acute lymphoblastic leukemia^1234^ Chronic myeloid leukemia^1235^ Acute myeloid leukemia^1236^	A subunit of PRC1 Negatively regulating p16INK4a and p14ARF/p19ARF expression at the transcriptional level Inhibiting E-cadherin expression^1214^

OCT4, SOX2, and Nanog are the core transcription factors that regulate the embryonic stem cell state.^314^ OCT4, SOX2, and Nanog are encoded by the *Sex-Determining Region Y (SRY)* gene,^315^ the *POU Domain, Class 5, Transcription Factor 1 (POU5F1)*,^316^ and the *Nanog* gene,^317^ respectively. They collaborate to positively regulate their promoters, activate the expression of genes necessary to maintain the embryonic stem cell state, and repress the expression of lineage-specific transcription factors.^205,314^ Similar stemness-maintaining functions of OCT4, SOX2, and Nanog have also been determined in adult stem cells.^318–321^ Expression of these transcription factors in cancer also endows stem-like properties to the cancer cells, unsurprisingly making them classical markers for CSCs.^322–324^ In addition, *SALL4*, encoded by a member of the Spalt-Like (SALL) gene family, *SALL4*,^325^ is also a transcription factor that regulates embryonic stem cell state by cooperating with Nanog.^326^ SALL4 expression is identified in several solid and hematopoietic malignancy types and correlates with CSC properties.^325^

Several cytoplasmic proteins are also identified as CSC markers. ALDHs refer to a group of enzymes that catalyze the oxidation of aldehydes to carboxylic acids, which can be further classified into 3 classes in mammals.^327^ Physiologically, ALDHs are present in most tissues of humans and have the highest concentration in livers, orchestrating drug metabolism.^328^ This also indicates an important role of ALDH in cancer drug resistance.^329^ ALDH activity has been considered a marker for not only normal stem cells but also CSCs of solid and hematopoietic malignancies.^330^

RNA-binding protein Musashi Homolog 1 and 2 (Musashi-1/2) are encoded by the *MSI1* gene and the *MSI2* gene, respectively.^331^ Both are RNA-binding proteins involved in post-transcriptional regulations of gene expressions and expressed in stem cells and progenitors to maintain their self-renewal.^332^ Musashi-2 also support hematopoiesis, which makes them a CSC marker in hematopoietic malignancies.^333–335^

Leucine Zipper-EF-Hand Containing Transmembrane Protein 1 (Letm1) is encoded by the *Letm1* gene, which is a transmembrane protein located in the inner membrane of mitochondria and functions as a Ca^2+^/H^+^ antiporter.^336^ In gastric, colorectal, and lung cancer, studies reveal a positive correlation between Letm1 and stemness-related signatures.^337–339^ Furthermore, suppressing or elevating the Letm1 expression leads to inhibited or enhanced stemness of colorectal cancer or osteosarcoma cells, respectively.^340,341^

Alpha-Fetoprotein (AFP, α-fetoprotein) is encoded by the *AFP* gene in humans, which is produced by the fetal liver and the yolk sac. The serum level of AFP peaks during embryogenesis and rapidly decreases after birth but re-increases in the presence of hepatocellular cancer or germ cell tumors, making it an evaluable biomarker for these two types of malignancies.^342^ Cells with high AFP levels exhibit stem-like properties in pancreatic cancer, cholangiocarcinoma, and hepatocellular cancer, making it a potential CSC marker for these types of cancer.^343–345^

Polycomb complex protein BMI-1, also known as polycomb group RING Finger Protein 4 (PCGF4) or RING Finger Protein 51 (RNF51), is encoded by the *BMI-1* gene. BMI-1 takes part in the repair of DNA double-strand breaks by homologous recombination^346^ and is essential for self-renewal in stem cells,^347,348^ which makes it also a marker for several types of solid tumor and hematopoietic CSCs.

Doublecortin-Like Kinase 1 (Dcamkl-1) is encoded by the *DCLK1* gene,^349^ which is a microtubule-associated protein that was recently revealed to have a role in regulating inflammation.^350^ Also, a study reports that Dcamkl-1 marks intestinal CSCs but not normal CSCs, making it an ideal marker for colorectal CSCs.^351^

A large variety of cell-surface proteins can be applied as CSC markers for solid tumors. C-X-C Chemokine Receptor Type 4 (CXCR4), also known as CD184, is a CXC chemokine receptor encoded by the *CXCR4* gene.^352^ The ligand for this receptor is CXCL12.^353^ CXCR4 is famous for its role as one of the receptors inducing the human immunodeficiency viruses (HIV) infection of T cells.^354^ CXCR4 is also involved in cancer progression for its role in activating the PI3K/AKT, PLC, hedgehog, ERK1/2, and JAK/STAT pathways.^355^

LGR5, also known as G-Protein Coupled Receptor 49 (GPR49) or G-Protein Coupled Receptor 67 (GPR67), is encoded by the *LGR5* gene.^356^ LGR5 has been identified as a part of the WNT signaling complex to potentiate the WNT/β-Catenin signaling.^357^ Given the crucial role of the WNT signaling in cancer stemness, LGR5 has also been identified as a cell-surface marker for several solid tumor types (Table 2).

Epithelial Cell Adhesion Molecule (EpCAM), also known as CD326, is known for its role in cell-cell adhesion in the epithelia,^358^ but its roles exceed this in cancer. Upon cleavage, the intracellular domain of EpCAM forms a complex with FHL2 and β-Catenin, which, with interaction with Lef1, leads to transcription of oncogenes, such as *c-Myc*.^359^ Besides, EpCAM also facilitates EMT by inhibiting E-cadherin.^359^

CD24, also known as Heat Stable Antigen (HSA), is encoded by the *CD24* gene in humans, which also functions as a cell-cell adhesion molecule.^360^ CD24 also mediates several signaling pathways that could lead to stemness enhancement of tumor cells.^361^ Likewise, CD44, also known as Homing Cell Adhesion Molecule (HCAM) and Phagocytic Glycoprotein-1 (Pgp-1)also induces cell-cell adhesion and interactions.^362^ It also takes part in activations of PI3K/AKT and Src/MAPK pathways and serves as a c-Met co-receptor.^362^ Both molecules can individually or combinedly mark CSCs in several solid tumor types. Moreover, the combination of CD44^+^/CD24^−^ also marks CSCs in breast cancer, prostate cancer, head and neck squamous cell carcinoma, and ovarian cancer (Table 2).

CD133, also known as Prominin-1 (PROM1) and encoded by the *PROM1* gene, belongs to the pentaspan transmembrane glycoproteins family.^363^ CD133 can activate the PI3K/AKT, Src, and β-Catenin signaling intracellularly to participate in cancer progression.^363^ CD133 is expressed in a wide range of human tissues and can serve as a CSC marker for various types of solid tumors and hematopoietic malignancies (Tables 2 and 3). The combined use of CD44 and CD133 as CSC markers has been reported in gallbladder cancer.^364^ Plus, CD44^+^/CD133^−^ and CD44^−^/CD133^+^ cells both can represent CSCs in colorectal cancer.^365,366^

**Fig. 3:**
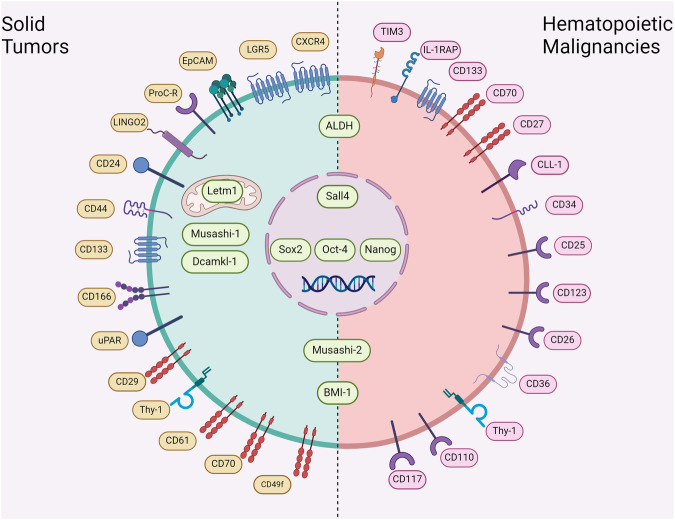
Biomarkers for CSCs in solid tumors and hematopoietic malignancies. Biomarkers for CSCs in solid tumors (left), hematopoietic malignancies (right), or both (center). The biomarkers can be classified into cell-surface markers and intracellular markers. Intracellular markers can be further classified into transcription factors that function in the nucleus and molecules that are found in the cytoplasm. Cell-surface markers make up the main differences between markers of solid tumors and those of hematopoietic malignancies

The differences between CSC markers for solid tumors and those for hematopoietic malignancies mainly lie in the variation of cell surface markers (Table 3) (Fig. 3). Interleukin-1 Receptor Accessory Protein (IL1RAP), encoded by the *IL1RAP* gene, is a receptor for interleukin-1.^367,368^ It has been identified as a CSC marker for myeloid leukemia.^369^ Similarly, CD25, a receptor for interleukin-2, and CD123, a receptor for interleukin-3, are also identified as CSC markers for AML or CML.^370–373^ CD70, expressed on the surface of various cells, and CD27, expressed on the T cell surface, are a pair of costimulatory molecules. The CD70/CD27 signaling is found activated in acute or chronic myeloid leukemia stem cells and contributes to the stemness formation of these cells by activating the WNT pathway.^374–376^ CD34^+^/CD38^−^ is also identified as a marker for myeloid leukemia stem cells and has been widely used.^2^ Compared to myeloid leukemia, CSC markers for lymphoblastic leukemia are hardly reported. A study suggests that CD90 and CD110 correlate with stemness of ALL cells and might be a CSC marker.^377^

Notably, a single CSC marker or a pair of CSC markers might not be sufficient to identify CSCs. For instance, while CD133^+^, CD166^+^CD44^+^, and CD24^+^CD44^+^ phenotypes of human colorectal cells do not correlate with stem cell properties, these 3 sets of markers are reported as CSC-specific in colorectal cancer.^378^ Plus, ALDH1 alone does not correlate with stem cell-like features in hepatocellular cancer cells,^379^ but CD133^+^ALDH^+^ cells are significantly more tumorigenic than their CD133^−^ALDH^+^ or CD133^−^ALDH^−^ counterparts,^380^ suggesting that combined use of CD133 and ALDH can better distinguish hepatocellular CSCs. Conversely, the absence of a CSC marker does not always indicate the absence of stemness. For instance, CD44^−^ head and neck squamous carcinoma cells also have stem-like features, although CD44 is a CSC marker for this type of cancer.^381^ This phenomenon indicates that these CSCs may have distinct origins. Indeed, in glioblastoma, CD133^+^ and CD133^−^ CSC respectively resemble fetal neural stem cells and adult neural stem cells, both of which exhibit stem-like properties.^382^ It is also noteworthy that certain CSC markers do not apply to every type of malignancy, even though it expressed in a wide range of tissues. For example, although ALDHs are present in most human tissues and represent a CSC marker for several cancer types, their activities play no functional role in stem cell-like properties in anaplastic thyroid cancer cells.^383^ Also, the CSC markers, CD133 and CD44, are generally overexpressed in gastrointestinal stromal tumors (GISTs) and cannot be used to distinguish CSCs from non-CSCs.^384^

## Molecular regulations in CSCs

### WNT/β-Catenin pathway

The WNT/β-Catenin signaling pathway, known for its involvement in various physiological processes and diseases, is evolutionarily conserved.^385^ Recent evidence highlights its crucial role in maintaining the stemness of CSCs. Chen et al. demonstrated its significance in converting mouse-iPSCs into CSCs.^386^ This pathway regulates stemness in CSCs across diverse cancer types, including lung, liver, thyroid, colorectal, cervical, and glioblastoma. For instance, in cervical cancer, cells with elevated Leucine-Rich Repeat-Containing G-Protein-Coupled Receptor 6 (LGR6) exhibit enhanced stemness, as LGR6 activates the WNT/β-Catenin pathway, forming a positive feedback loop with Transcription Factor 7-Like 2 (TCF7L2).^387^ Similarly, LSD1 maintains stemness in thyroid cancer by targeting Adenomatous Polyposis Coli 2 (APC2) or indirectly regulating Dickkopf WNT Signaling Pathway Inhibitor 1 (DKK1) via the HIF-1α/miR-146a axis to antagonize the WNT pathway.^94^ In liver cancer, EPHB2 sustains tumor stemness by activating the SRC/β-Catenin cascade. The WNT/β-Catenin pathway, in turn, upregulates EPHB2 expression in a TCF1-dependent manner, forming a positive feedback loop linked to liver CSCs.^95^ Furthermore, non-coding RNAs play a pivotal role in stemness maintenance. For example, Protein Kinase Membrane-Associated Tyrosine/Threonine 1 (PKMYT1) associated lncRNA sponges miR-485-5p to upregulate PKMYT1, inhibiting β-transducin repeat containing protein 1 (β-TrCP1)-mediated β-Catenin degradation and activating WNT signaling in NSCLC stem cells.^388^ Similarly, in liver CSCs, lncRNA Small Nucleolar RNA Host Gene 5 (lncSNHG5) activates the WNT/β-Catenin pathway by inhibiting Upstream Frameshift 1 (UPF1), sustaining stemness.^302^ Additionally, overexpression of LINC00839 in GSCs via Methyltransferase-Like 3 (METTL3)-mediated m6A modification enhances c-Src-driven phosphorylation of β-Catenin, activating WNT signaling and promoting stemness.^389^ Likewise, in colorectal cancer, Sec62, induced by METTL3-mediated m6A modification, enhances β-Catenin nuclear translocation, reducing its ubiquitination degradation and promoting cancer stemness.^390^

The involvement of the WNT/β-Catenin signaling pathway in CSCs contributes to malignant behaviors such as tumorigenesis and differentiation. Kim et al. showed that colorectal cancer cells expressing CD44 and CD133, markers of CSCs, exhibit strong tumor-initiating effects, accompanied by significant activation of the WNT/β-Catenin pathway.^391^ Furthermore, a CD44^+^Cellular Prion Protein (PrPc^+^) LGR4^+^ CSC subpopulation in colorectal cancer demonstrates high metastatic potential, with LGR4 and PrPC activating the WNT/β-Catenin pathway.^392^ Far Upstream Element-Binding Protein 1 (FUBP1) upregulation in colorectal cancer activates the WNT/β-Catenin cascade, enhancing stemness and potentially driving tumorigenesis.^393^ In breast cancer, Calreticulin (CALR) promotes a stem cell phenotype, with upregulation by HIF-1 activating the WNT/β-Catenin pathway to facilitate tumor initiation.^394^ Piwi-Like RNA-Mediated Gene Silencing 2 (Piwil2)-overexpressing cervical cancer cells exhibit strong stemness, partly attributed to the WNT/β-Catenin pathway, inhibition of which induces cell differentiation and suppresses tumorigenicity.^395^

The WNT/β-Catenin signaling pathway in CSCs is involved in the metastasis process. Husain et al. demonstrated that Farnesyl Dimethyl Chromanol (FDMC), an inhibitor of the WNT/β-Catenin pathway, suppresses the stemness and metastatic potential of colorectal CSCs, inducing their apoptosis.^396^ Colorectal cancer exhibits overexpression of Disheveled3 (DVL3), activating the WNT/β-Catenin/c-Myc/SOX2 signaling cascade, thereby enhancing stemness and metastatic potential.^397^ In gastric cancer, ST2^+^ serves as a functional marker of CSCs and activates the WNT signaling pathway, promoting metastasis through interaction with BCL-XL.^398^ Similarly, in pancreatic cancer, upregulated Frizzled-7 (FZD7) promotes CSC phenotype and liver metastasis via the canonical WNT/β-Catenin pathway.^399^ Additionally, polychlorinated biphenyls 2,3,5-trichloro-6-phenyl-[1,4]-benzoquinone (PCB29-pQ) activates the WNT/β-Catenin pathway, enhancing breast cancer stemness and metastasis.^400^

Most studies have consistently shown a positive correlation between the activation of the WNT/β-Catenin pathway and the malignant behavior of CSCs. However, in radioresistant glioblastoma, the expression of N-cadherin correlates positively with the inhibition of the WNT/β-Catenin signaling pathway. N-cadherin binds to β-Catenin in the cytoplasm, inhibiting neuronal differentiation mediated by the WNT signaling pathway and maintaining a stem-like phenotype.^401^ Conversely, in ameloblastoma, β-Catenin expression is negatively correlated with the CSCs’ marker SOX2. Exogenous activation of the WNT/β-Catenin signaling pathway leads to the inhibition of tumor stemness and invasiveness.^402^ These findings suggest that the role of the WNT/β-Catenin signaling pathway in CSCs is complex and may vary across different cancer types and states.

### Hedgehog pathway

The classic hedgehog signaling pathway encompasses several cascades. Initially, Patched (PTCH) binds to the hedgehog ligand, relieving the inhibition of Smoothened (SMO). This event further facilitates the dissociation of the Suppressor of Fused (SuFu) from GLI, allowing GLI activators to regulate target genes.^403^ Yan et al. demonstrated that the interaction between glioma cells and endothelial cells activates the hedgehog pathway, promoting the transformation of glioma cells into a GSC phenotype.^404^ The regulation of the hedgehog pathway in CSCs is intricately linked to their emergence and various malignant biological behaviors.^405^

The hedgehog signaling pathway plays a pivotal role in maintaining the stemness of CSCs. Kelch Domain-Containing 8 A (KLHDC8A) has been identified in GSCs as an upstream factor involved in maintaining stemness by activating the hedgehog signaling pathway through ciliogenesis.^406^ Similarly, Liu et al. revealed the existence of the ISL1/sonic hedgehog (SHH)/GLI1 axis, which promotes GSCs’ stemness.^407^ The elimination of the liver CSCs’ stemness maintainer Ubiquitin-Like With PHD And Ring Finger Domains 1 (UHRF1) results in extensive DNA hypomethylation, ultimately upregulating CEBPA to inhibit the hedgehog pathway.^408^ Additionally, miR-324-5p weakens the function of multiple myeloma stem cells by inhibiting the hedgehog signaling pathway.^409^ Guen et al. demonstrated the connection between the EMT program and stemness, showing that tumor-initiating cells activate the hedgehog pathway through the EMT program to enhance stemness.^410^

Furthermore, the hedgehog signaling pathway is implicated in the tumor-initiating function of CSCs. In liver CSCs, the circIPO11/Topoisomerase 1 (TOP1)/GLI1 axis associated with liver cancer initiation has been identified. TOP1 is recruited to the *GLI1* promoter by circIPO11 to activate the hedgehog pathway, promoting stemness and tumor initiation.^411^ Mok et al. revealed that cholesterol-related pathways are significantly upregulated in liver CSCs compared to normal stem cells. The hedgehog signaling pathway is activated in hepatic CSCs as a downstream factor for cholesterol synthesis mediated by the caspase-3/Sterol-Regulatory Element-Binding Protein 2 (SREBP2) axis, ultimately maintaining stemness and tumorigenicity.^412^ Similarly, TRNA Methyltransferase 6 (TRMT6)/TRMT61A-mediated N1-methyladenosine methylation in liver CSCs promotes cholesterol metabolism and activates the hedgehog pathway to maintain stemness and enhance tumorigenicity.^413^ In breast CSCs, the activated hedgehog signaling pathway is positively associated with stemness maintenance and tumorigenicity. Overexpression of Tetraspanin-8 (TSPAN8) relieves the inhibition of SMO by PTCH1 and phosphorylates SMO by promoting the binding of PTCH1 to SHH1, recruiting Ataxin-3 (ATXN3) to reduce the ubiquitination degradation of the SHH/PTCH1 complex, ultimately promoting GLI1 transcription.^414^ Additionally, Polypeptide N-Acetylgalactosaminyltransferase 1 (GALNT1)-mediated glycosylation of SHH in bladder cancer activates the hedgehog pathway, increasing the stemness and tumorigenicity of CSCs.^415^ Immunity may also play a significant role in influencing the effects of the hedgehog pathway in CSCs. IL-25, an intrinsic hedgehog pathway agonist, promotes CSCs’ function, increasing colitis-related tumorigenesis through the accumulation of GLI1.^416^

It is widely recognized that CSCs participate in the process of metastasis by activating the hedgehog signaling pathway.^417^ Upregulated Ubiquitin-Specific Peptidase 37 (USP37) in breast CSCs binds and stabilizes GLI1 to activate the hedgehog pathway, which further regulates the stemness and metastatic potential of CSCs.^418^ *GLI1* was identified as a key regulatory gene for colorectal cancer stemness, and activation of the Hh/GLI1 signaling cascade was positively correlated with the invasiveness of colorectal CSCs.^419^ Disc Large Homolog 5 (DLG5), an activator of the hedgehog signaling pathway in glioblastoma. DLG5 prevents ubiquitination and degradation of GLI1 to promote the migration and stemness maintenance of GSCs.^420^

### Notch pathway

The Notch pathway comprises several main components: the Notch receptor, Notch ligand, CBF-1, suppressor of hairless, Lag (CSL), DNA binding protein, and downstream target genes. Initially discovered by Drosophila,^421^ the Notch pathway has been shown to play a crucial role in promoting the formation of medulloblastoma stem cells.^422^ It is implicated in maintaining the stemness of CSCs, as evidenced by its upregulation in supratentorial ependymoma and mucoepidermoid carcinoma, where it correlates positively with the expression of CSCs’ markers.^423,424^ Additionally, syndecan-1 in inflammatory breast CSCs acts as a molecular marker maintaining their stem phenotype by activating the Notch pathway.^425^ While most studies support the positive relationship between Notch pathway activation and stemness maintenance, Högström et al. reported that upregulation of the Notch pathway attenuated the stemness of Prospero Homeobox 1 (PROX1^+^) colorectal cancer cells.^426^

Moreover, activation of the Notch pathway in CSCs has been associated with metastasis in various tumors such as breast cancer, glioma, renal cancer, and ovarian cancer. In breast cancer, Bone Morphogenetic Protein 4 (BMP-4) promotes stemness and EMT programs by activating the Notch pathway in a Smad4-dependent manner.^427^ Similarly, Signal Peptide CUB Domain And EGF-Like Domain Containing 2 (SCUBE2) overexpression in breast cancer cells enhances tumorigenicity and metastatic potential by activating the Notch pathway.^428^ Family With Sequence Similarity 129 Member A (FAM129A) prevents ubiquitination and degradation of Notch1, upregulating the Notch pathway to maintain the stemness and metastatic potential of GSCs.^429^ Notably, the upregulated Notch pathway in renal CSCs contributes to multiple malignant biological behaviors, including metastasis, stemness maintenance, and tumorigenesis.^430^ Additionally, glycosyltransferase GnT-III-mediated bisecting glycosylation of Notch1 effectively activates the Notch pathway, supporting stemness maintenance and metastasis.^431^

Activation of the Notch pathway in CSCs is associated with tumorigenesis, differentiation, and immune regulation. Liposarcoma cells with continuous activation of the Notch pathway exhibit overexpression of CSCs’ marker genes, leading to enhanced tumorigenesis compared to cells with normal Notch activity.^432^ Speckle-Type POZ Protein-Like (SPOPL), a stemness maintainer highly expressed in GSCs, activates the Notch pathway, thereby increasing tumorigenicity.^433^ Inhibition of the Notch pathway in GSCs induces significant neuronal differentiation and reduces stemness.^434^ Similarly, lncRNA FOXD2 Adjacent Opposite Strand RNA 1 (FOXD2-AS1) recruits TATA-Box Binding Protein Associated Factor 1 (TAF-1) to the promoter of *Notch1*, initiating the Notch signaling pathway in GSCs. Inhibition of FOXD2-AS1 induces the apoptosis and differentiation of GSCs while attenuating their stemness.^435^ Additionally, the Notch pathway plays a crucial role in immune system regulation. Expression of histone methyltransferase G9a in GSCs positively correlates with stemness characteristics. G9a binds to the Notch suppressor F-Box And WD Repeat Domain Containing 7 (FBXW7), upregulating the Notch pathway and enhancing the expression of PD-L1 in GSCs. This, in turn, weakens the function of T lymphocytes, creating an immunosuppressive microenvironment.^436^

### NF-κB pathway

The NF-κB pathway, consisting of several cascades, is activated when cells encounter various stimuli, leading to the degradation of I-kappa B (IκB) protein by IκB kinase activation. This degradation releases NF-κB dimers, which are further activated through various post-translational modifications and translocated to the nucleus. There, they bind to target genes, promoting the transcription of these genes.^437^ NF-κB activation plays a critical role in the formation of breast CSCs.^438^ Pathway analysis of CSCs isolated from prostate cancer and NSCLC patient revealed the specific activation of the NF-κB pathway, suggesting its potential as an effective therapeutic target.^439,440^ Evaluation of the NF-κB signature in patient-derived GSCs can accurately predict the prognosis of low-grade glioma.^441^

Moreover, the NF-κB pathway is implicated in maintaining stemness. Calcium Calmodulin-Dependent Protein Kinase II γ (CaMKIIγ), identified as a marker of AML stem cells, maintains stemness by activating the 5-LO/NF-κB pathway.^442^ In ovarian CSCs, NF-κB pathway-related proteins are highly expressed, and inhibiting the NF-κB pathway reduces the CSC population.^443^ The lncRNA ASB16 Antisense RNA 1 (ASB16-AS1) cooperates with ATM kinase to phosphorylate Tripartite Motif Containing 37 (TRIM37), activating the NF-κB pathway and promoting gastric cancer cell stemness.^444^ Additionally, the Let-7a/Ras/NF-κB axis acts as a stemness antagonistic pathway in breast CSCs, with Let-7a inactivating the NF-κB pathway in a Ras-dependent manner.^445^ Overexpression of S100 Calcium-Binding Protein A4 (S100A4) activates the Inhibitor Of Kappa B Kinase (IKK)/NF-κB signaling pathway, contributing to the stemness maintenance of bladder CSCs.^446^

The activated NF-κB pathway in CSCs is intimately linked to tumorigenesis and metastasis. The transition from a proneural to mesenchymal phenotype (PMT) characterizes the conversion of less aggressive proneural GSCs into highly aggressive mesenchymal GSCs.^447,448^ Fos-Like Antigen 1 (FOSL1) has been identified as a key regulator of PMT, upregulating Ubiquitin-Conjugating Enzyme (UBC9) to enhance the SUMOylation of Cylindromatosis (CYLD). This process activates the NF-κB pathway, supporting the PMT program of GSCs.^449^ Similarly, Mixed Lineage Kinase 4 (MLK4) binds to phosphorylated IKKa, activating the NF-κB pathway and facilitating the transformation of GSCs into the mesenchymal phenotype.^450^ Upregulated BMI-1 in CD133^+^ liver CSCs enhances NF-κB activation and nuclear translocation, promoting CSC stemness and metastatic potential while inhibiting apoptosis.^451^ The estrogen metabolite 2-methoxy estradiol (2-ME2) disrupts the NF-κB/HIF-1 axis, reversing the EMT program and abolishing the metastatic potential of nasopharyngeal carcinoma stem cells.^452^ Stromal Cell-Derived Factor-1 (SDF-1) overexpression in breast cancer induces stemness and EMT phenotypes by activating the NF-κB pathway.^453^ Additionally, miR-221/222 inhibits Phosphatase And Tensin Homolog (PTEN), leading to NF-κB activation and enhanced stem cell characteristics, tumorigenesis, and metastasis in breast cancer cells.^454^ A positive feedback loop involving DiGeorge Syndrome Critical Region 8 (DGCR8)/circKPNB1/SPI1/DGCR8 promotes stemness in GSCs, with SPI1 upregulating the NF-κB pathway in a TNF-α-dependent manner, thereby promoting tumorigenesis.^455^

### JAK/STAT pathway

The JAK/STAT pathway consists of three main components: tyrosine kinase-related receptors that receive signals, tyrosine kinase JAK that transmits signals, and transcription factors STAT.^456^ Upon binding of various stimulatory factors to the receptor, JAK is phosphorylated and activated, subsequently recruiting and phosphorylating the transcription factor STAT. This phosphorylated STAT then forms dimers and is translocated to the nucleus, where it binds to target genes, regulating downstream gene expression.^457^

Regulation of the JAK/STAT pathway is closely linked to the maintenance of stemness. Misra et al. demonstrated that selective inhibition of STAT3 significantly reduced the expression of stemness-related genes in breast CSCs.^458^ Alpha-casein acts as a STAT pathway antagonist, inhibiting the STAT3/HIF-1α axis and impairing the function of breast CSCs.^459^ Moreover, activation of the lipid metabolism-related STAT3/CPT1B/fatty acid β-oxidation (FAO) axis in breast CSCs correlates positively with stemness maintenance.^460^ Similarly, STAT pathway activation contributes to stemness maintenance in osteosarcoma, liposarcoma, and thyroid cancer.^461–463^ Immunity may play a significant role in JAK/STAT pathway regulation in CSCs. IL-17E/IL-25 secreted by non-CSCs binds to IL-17 Receptor B (IL-17RB) on CSCs, activating the JAK/STAT3 pathways to regulate liver CSCs’ stemness.^464^ Additionally, IL-6 is secreted by regulatory T cells, which upregulates the STAT3 pathway in glioma cells, maintaining the stemness-associated phenotype.^465^

Regulation of the JAK/STAT pathway in CSCs is intricately linked to tumorigenesis, metastasis, and metabolic reprogramming. Kanno et al. demonstrated that Von Hippel-Lindau (VHL) inhibits the JAK2/STAT3 signaling pathway, thereby reducing the tumorigenic ability of GSCs.^466^ In prostate CSCs, IL-6-mediated activation of the JAK/STAT pathway is a crucial event in tumorigenesis, and its inhibition eliminates tumor initiation.^467^ LIM Domain Only 2 (LMO2) acts as an endogenous agonist of the JAK/STAT pathway by forming a complex with LIM Domain-Binding 1 (LDB1) that phosphorylates STAT3, promoting the expression of ID1 and thereby upregulating the stemness and metastatic potential of GSCs.^468^ Leptin, an adipocyte-derived hormone, activates the JAK/STAT pathway in gastric cancer cells, maintaining their stemness and metastatic potential.^469^ Interferon-Induced Transmembrane Protein 3 (IFITM3), derived from GSCs, activates the JAK/STAT3 pathway to upregulate Basic Fibroblast Growth Factor (bFGF) expression, promoting angiogenesis in glioblastoma, a critical step in metastasis.^470^ Contrary to the STAT3/CPT1B/FAO axis, which is activated to maintain the stemness of breast CSCs, viperin overexpression in CSCs partially inhibits FAO through the JAK/STAT pathway, thereby reprogramming metabolism to promote tumor progression.^460,471^

### TGF-β pathway

The TGF-β family ligands form a complex with receptors on the membrane, and the activated receptor kinase recruits and activates downstream Smad proteins, thereby inducing nuclear transfer of Smad proteins and exerting transcriptional regulation.^472^ The TGF-β pathway plays a pivotal role in embryonic development, immune surveillance, and maintenance of homeostasis.^473^ Dysregulation of the TGF-β pathway in CSCs is closely associated with the occurrence and progression of tumors.^474^ Nakano et al. demonstrated that stimulation of the TGF-β pathway triggers the conversion of CD44^−^ non-colorectal CSCs into CD44^+^ colorectal CSCs. Moreover, sustained activation of the TGF-β pathway is crucial for colorectal CSCs to maintain an undifferentiated state.^141^ Similarly, activation of the TGF-β signaling pathway has been observed during breast CSC generation.^475^

Activation of the TGF-β pathway in CSCs is closely linked to tumorigenesis and stemness maintenance. The U2 Auxiliary Factor 65 (U2AF65)/circNCAPG/Ras-Responsive Element-Binding Protein 1 (RREB1) positive feedback loop was identified in GSCs, where U2AF65 binds to and stabilizes circNCAPG, thereby stabilizing RREB1 and promoting its nuclear translocation. Accumulated RREB1 activates the TGF-β1 pathway to maintain the stemness of GSCs and promote tumorigenesis.^476^ Similarly, Heat Shock Protein 47 (HSP47) induces the stemness and tumorigenesis of GSCs by activating the TGF-β pathway.^477^ Lymphoid Enhancer-Binding Factor 1 (LEF1) directly binds to and upregulates the expression of ID1, triggering the TGF-β pathway, which in turn promotes the stemness-associated phenotype and tumorigenicity of esophageal squamous cell carcinoma.^478^ Wang et al. discovered that CD51, a marker of colorectal CSCs, activates the TGF-β/Smad signaling pathway to support tumorigenesis.^479^ The Hematological And Neurological Expressed 1-Like (HN1L) overexpression triggers the TGF-β pathway by upregulating FOXP2, ultimately maintaining stemness and promoting tumorigenesis of prostate cancer.^480^

Activation of the TGF-β pathway in CSCs plays a pivotal role in tumor metastasis. Wen et al. demonstrated that targeted inhibition of the TGF-β/Smad pathway effectively eliminated the EMT program and metastatic potential of ovarian CSCs.^481^ FZD7 activates the TGF-β1/Smad3 pathway to confer stemness to pancreatic cancer cells. Further evidence suggests that upregulation of FZD7/TGF-β1/Smad3 promotes the EMT program to support pancreatic cancer liver metastasis.^399^ Similarly, Epithelial Membrane Protein 3 (EMP3) in lung CSCs interacts with TGF-β Receptor Type 2 (TGFBR2) to activate the TGF-β/Smad pathway, subsequently upregulating stemness and promoting the EMT program.^482^ Activation of the SIX Homeobox 1 (Six1)/Eyes Absent (EYA)/TGF-β pathway mediates CSC characteristics and EMT programs in breast cancer.^483^ Additionally, the interaction between miRNAs and the TGF-β pathway is a critical factor affecting tumor metastasis. MiR-495, identified as a stemness suppressor in oral squamous cell carcinoma, inhibits Homeobox C6 (HOXC6), thereby inhibiting the TGF-β pathway to prevent stemness characteristics and the EMT program of CSCs and induce their apoptosis.^484^ MiR-106b attenuates the expression of the inhibitory Smad protein Smad7 to trigger the TGF-β pathway and promote the EMT program.^485^ Angiogenesis is a crucial aspect of the metastatic cascade.^486^ Chen et al. identified Paired-Related Homeobox 1 (Prrx1) as a non-GSC stemness-promoting factor and a GSC stemness-maintaining factor in glioma. Prrx1 directly binds to the *TGF-β1* promoter region to activate the TGF-β/Smad pathway, which in turn upregulates stemness and promotes vascularization in the tumor microenvironment.^487^

### PI3K/AKT pathway

As a pivotal factor in the PI3K/AKT pathway, AKT undergoes structural changes and activation by PI3K, which subsequently modulates a cascade of downstream substrates to regulate various cellular behaviors.^488^ The mTOR is a classic downstream target of the PI3K/AKT pathway, while PTEN acts as a negative regulator by dephosphorylating AKT to suppress downstream signaling.^489^ The involvement of the PI3K/AKT pathway in driving the differentiation of normal stem cells into CSCs has been confirmed.^490,491^ Moreover, this pathway is closely associated with the maintenance of stemness in CSCs. Madsen et al. demonstrated a positive correlation between PI3K/AKT/mTOR pathway activation and breast cancer stemness score.^492^ Additionally, activation of the insulin/insulin-like growth factor signaling (IIS) pathway in breast CSCs further potentiates the PI3K/AKT pathway to sustain MYC expression, thereby enhancing the stemness traits of breast CSCs.^493^ Furthermore, PD-L1 contributes to the establishment of a suppressive immune microenvironment.^494^ Almozyan et al. revealed that the continuously activated PI3K/AKT pathway by PD-L1 is pivotal in maintaining the stemness of breast CSCs.^495^

Activation of the PI3K/AKT pathway in CSCs is intricately linked to tumorigenicity. Activation of the PI3K/AKT pathway and the MAPK/ERK pathway respectively promote and inhibit the stemness signatures and tumorigenic potential of lung cancer.^496^ The liver cancer tumor suppressor Connexin 32 (Cx32) attenuates the activity of the PI3K/AKT pathway, thereby suppressing stemness and tumorigenicity.^497^ The tumor suppressor miR-30a binds to and inhibits 5’-Nucleotidase Ecto (NT5E), thus downregulating the activity of the NT5E-mediated PI3K/AKT pathway, thereby impeding the stemness and tumorigenicity of GSCs.^498^ Non-coding RNAs also play a regulatory role in the PI3K/AKT pathway. Tumor suppressors miR-873 and miR-30a bind and inhibit patterns of Pleckstrin-2 (PLEK2) and NT5E respectively, leading to downregulation of the PLEK2 or NT5E-mediated PI3K/AKT pathway, thus hindering stemness and tumorigenicity of pancreatic CSCs and GSCs.^498,499^ Similarly, miR-3187-3p, which can be sponged by circ_0000745, inhibits Erb-B2 Receptor Tyrosine Kinase 4 (ERBB4), thereby attenuating the activity of the PI3K/AKT pathway, exerting a suppressive effect on the tumorigenicity and stemness of ovarian cancer.^500^

The activation of the PI3K/AKT pathway in CSCs is intricately linked to metastasis. AKT-mediated phosphorylation of Testis-Specific Y-Like Protein 5 (TSPYL5), a factor involved in stemness maintenance, impedes its ubiquitination and degradation. Phosphorylated TSPYL5 further inhibits negative regulators of the PI3K/AKT pathway, forming an AKT/TSPYL5/PTEN positive feedback loop that sustains the expression of stemness-related genes and promotes EMT programs.^501^ In head and neck squamous cell carcinoma, activation of the PI3K/AKT/mTOR pathway upregulates SOX2, promoting the maintenance of the stemness phenotype and the E-cadherin-mediated EMT program.^502^ Stress-Induced Phosphoprotein 1 (STIP1) in osteosarcoma enhances MMP-2 and MMP-9 by activating the PI3K/AKT and ERK1/2 pathways, ultimately promoting osteosarcoma CSC metastasis.^503^ In breast CSCs, Transmembrane And Coiled-Coil Domain Family 3 (TMCC3) binds AKT to activate the PI3K/AKT pathway, thereby supporting tumorigenesis and metastasis.^504^ CAFs are pivotal in supporting metastasis. CAFs upregulate TNF Receptor Superfamily Member 19 (TNFRSF19/TROY), a marker of liver CSCs, which activates the PI3K/AKT/T-Box Transcription Factor 3 (TBX3) pathway by promoting polyubiquitination of the PI3K inhibitory subunit p85α. Accumulated TBX3 maintains stemness and promotes metastasis.^505^ Similarly, CAFs-secreted periostin induces the phosphorylation of FAK to activate AKT, enriching CSCs in the gastric cancer cell population.^216^ Moreover, Liang et al. demonstrated that inhibition of the PI3K/AKT pathway attenuates the stemness characteristics and angiogenesis of endometrial cancer, which are closely associated with distant metastasis.^506,507^

### PPAR pathway

PPARs, belonging to the nuclear hormone receptor family, are ligand-activated receptors that regulate various metabolic processes like fat and glucose metabolism. There are three main subtypes: PPARα, PPARδ/β (PPARD), and PPARγ (PPARG).^508^

The activation of the PPAR pathway plays a pivotal role in maintaining the stemness of CSCs. In liver CSCs, activation of the PPARα pathway and the enrichment of its downstream factor, Stearoyl-CoA Desaturase 1 (SCD1), contribute to the stemness characteristics.^509^ Similarly, increased PPARγ activity has been observed in melanoma stem cells.^510^ Co-culturing MSCs with gastric cancer cells leads to the enrichment of lncRNA Histocompatibility Leukocyte Antigen Complex P5 (HCP5) in MSC-stimulated gastric cancer cells, which sponges miR-3619-5p to promote the expression of PPARG Coactivator 1 Alpha (PPARGC1A). PPARGC1A accumulation triggers the PPAR Coactivator-1α (PGC1α)/CCAAT Enhancer Binding Protein Beta (CEBPB)/CPT1 axis, inducing FAO and stemness characteristics.^230^

Conversely, inhibition of the PPAR pathway has also been associated with maintaining CSC stemness. Activation of PPARγ induced by inhibiting TRAF2- and NCK-Interacting Protein Kinase (TNIK) correlates with the reduction of osteosarcoma cell stemness and their differentiation into adipocytes.^511^ Downregulation of PPARD in the acidic microenvironment of colorectal cancer inhibits Vitamin D Receptor (VDR) expression, promoting the emergence of a CSC phenotype.^512^ Similarly, PPARγ activation effectively inhibits the stem cell phenotype of bladder cancer.^513^ Moreover, besides fat metabolism, the PPAR pathway also regulates CSC characteristics through glucose metabolism. Low expression of PPARα in AML CSCs inversely correlates with their stemness characteristics. PPARα binds to HIF1α, inhibiting the expression of its downstream *Phosphoglycerate Kinase 1 (PGK1)* gene, ultimately weakening glucose metabolism activity and inhibiting stemness.^514^

Activation of the PPAR pathway in CSCs is closely associated with tumorigenicity, differentiation, and metastasis. GSCs exhibit overexpression of PPARα compared to normal neural stem cells. Knockdown of *PPARα* significantly reduces the expression of stemness-related genes and fat metabolism-related genes in GSCs, leading to a decrease in tumorigenicity.^515^ In hepatic CSCs, fatty acid 4-phenylbutyric acid (4-PBA) upregulates the expression of PPARα, preventing its degradation, thereby promoting the initiation and tumorigenicity of hepatic CSCs.^516^ N1-methyladenosine methylation-driven expression of PPARδ in hepatic CSCs activates the PPAR pathway, regulating cholesterol metabolism to maintain stemness and enhance tumorigenicity.^413^ Activation of PPARδ has been implicated in colorectal cancer liver metastasis induced by a high-fat diet, where it increases Nanog transcription.^517^ Moreover, PPARα activation is positively correlated with the invasive and stemness phenotypes of GSCs^518^. Conversely, stimulation of PPARγ may inhibit the migration ability of GSCs.^519^ Activation of PPARγ has also been shown to downregulate the stemness of brain CSCs and induce the expression of differentiation-related genes such as *Collagen Type II Alpha 1 (COL2A1)* and *Motor Neuron And Pancreas Homeobox 1 (HLXB9)*.^520^ Similarly, PPARγ activation reduces the activity of *SOX2* and *YAP1* genes, inhibiting the stemness of osteosarcoma stem cells and promoting their differentiation.^521^

### Molecular crosstalks

The formation and maintenance of CSCs involve complex interactions between multiple signaling pathways. For instance, SCD1 has been identified as a target for colorectal CSCs, inhibiting both WNT and Notch signaling pathways simultaneously to maintain the stemness-associated phenotype.^522^ Similarly, NK6 Homeobox 1 (NKX6-1) in leiomyosarcoma upregulates stemness by activating Notch and SHH pathways.^523^ Tumors with high expression of Notch and hedgehog signaling pathways exhibit stronger stemness, often associated with a hypoxic microenvironment and activation of regulatory T cells.^524^ Protein kinase CK2 activates AKT, NF-κB, and STAT3 pathways to maintain the stemness of AML cells.^525^ Breast CSCs overexpressing Cyclooxygenase-2 (COX-2) activate PI3K/AKT, Notch, and WNT pathways via E-type Prostaglandin Receptor 4 (EP4), contributing to breast cancer metastasis.^526^ Moreover, Frizzled10 (FZD10) activation in liver CSCs through N6-methyladenosine methylation mediated by METTL3 stimulates the WNT and Hippo pathways, critical for hepatic CSC self-renewal.^527^

**Fig. 4:**
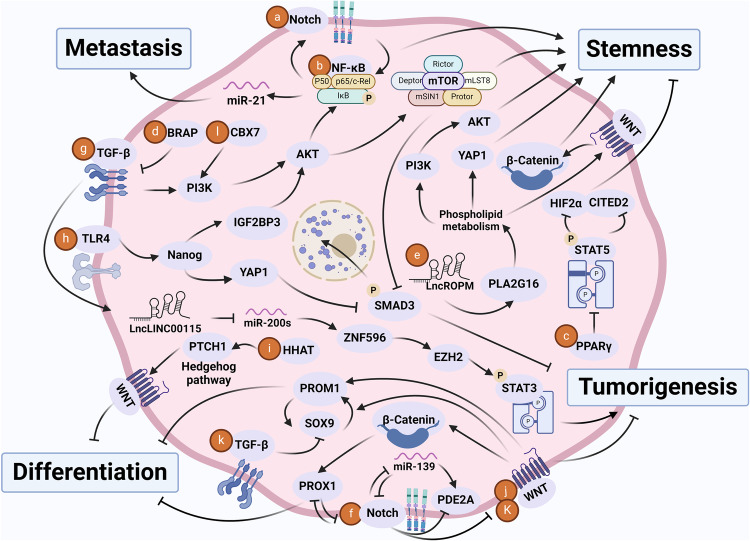
Crosstalk of signaling pathways in CSCs. **a**, **b** The Notch pathway can be activated by the NF-κB pathway while activating the NF-κB pathway. **c** PPARγ inhibits the STAT5 pathway to downregulate the expression of HIF2α and CITED2, ultimately attenuating the stemness characteristics of CSCs. **d** BRAP inhibits the TGF-β/PI3K/AKT/mTOR axis to reduce the stemness of CSCs. **e** LncROPM upregulates PLA2G16 expression to facilitate phospholipid metabolism, which subsequently activates the PI3K/AKT, WNT/β-Catenin, and Hippo/YAP pathways to maintain the stemness of CSCs. **f** Amplified miR-139 through the miR-139/PDE2A/Notch1 loop, inhibits the WNT pathway to attenuate the tumorigenicity of CSCs. **g** LINC00115, upregulated by the TGF-β pathway, sponges miR-200s to activate the ZNF596/EZH2/STAT3 axis to promote the stemness and tumorigenesis of CSCs. **h** Activation of the TLR4/NANOG axis subsequently upregulates the YAP1/SMAD3 and IGF2BP3/AKT/mTOR/SMAD3 pathways to inhibit the nuclear transfer and phosphorylation of SMAD3, ultimately attenuating the tumorigenicity of CSCs. **i** WNT/β-Catenin pathway downstream effector PROX1 inhibits each other with Notch1, thereby elevating the stemness of CSCs and hindering their differentiation. **j** PROX1, which can be activated by the WNT/β-catenin pathway, inhibits each other with Notch1, thereby enhancing the stemness of tumor cells and hindering their differentiation. **k** SOX9/PROM1 positive feedback loop in inhibits differentiation by activating the CSC program, which positively correlates with WNT pathway and negatively correlates with TGF-β pathway. **l** The PI3K/AKT pathway, activated by CBX7, further stimulating the NF-κB/miR-21 axis, and ultimately promoting the stemness characteristics and metastasis of tumor cells

The formation and the stemness of CSCs are supported by crosstalk between multiple pathways. IL-6 and NO secreted by MDSCs activate STAT3 and Notch signaling in breast cancer cells, collectively inducing CSC formation.^288^ Notch signaling can drive NF-κB pathway-related gene expression in skin CSCs (Fig. 4a).^528^ Notably, NF-κB pathway upregulation also activates the Notch pathway to support breast CSC expansion (Fig. 4b).^529^ Additionally, activated PPARγ inhibits the STAT5 pathway, downregulating HIF2α and Cbp/P300 Interacting transactivator with Glu/Asp-Rich Carboxy-Terminal Domain 2 (CITED2) expression, which are protectors of CML CSCs (Fig. 4c).^530^ Lastly, Breast Cancer Susceptibility Gene 1-Associated Protein (BRAP) inhibits the TGF-β/PI3K/AKT/mTOR pathway, weakening the stem cell properties of GSCs (Fig. 4d).^531^ LncROPM exerts a direct binding effect on Phospholipase A And Acyltransferase 3 (PLA2G16), thereby augmenting its expression and facilitating phospholipid metabolism. This process subsequently activates the PI3K/AKT, WNT/β-Catenin, and Hippo/YAP pathways to maintain the characteristics of breast CSCs (Fig. 4e).^532^

The tumorigenicity, differentiation, and metastasis capabilities of CSCs are regulated by the intricate crosstalk among multiple signaling pathways. In GSCs, the miR-139/Phosphodiesterase 2 (PDE2A)/Notch1 loop inhibits stemness and tumorigenicity by suppressing WNT signaling (Fig. 4f).^533^ Chronic hypoxia-induced HIF-2α overexpression activates WNT and Notch pathways, leading to enhanced stemness-associated phenotype and tumorigenesis in breast CSCs.^534^ Additionally, LINC00115, activated by the TGF-β pathway, upregulates ZEB1 and Zinc Finger Protein 596 (ZNF596) expression to activate ZNF596/Enhancer Of Zeste Homolog 2 (EZH2)/STAT3, promoting the stemness and tumorigenesis of GSCs (Fig. 4g).^535^ Activation of the AKT and YAP pathways in CSCs may inversely correlate with tumorigenesis. In liver cancer-initiating stem cells, the Toll-like receptor 4 (TLR4)/Nanog/YAP1/insulin-like growth factor 2 mRNA-binding protein 3 (IGF2BP3) axis inhibits TGF-β pathway activity and tumor-initiating ability, which can be counteracted by TGF-β pathway activation (Fig. 4h).^536^ The interaction between the WNT pathway and hedgehog, Notch, and TGF-β pathways influences the differentiation of colorectal CSCs. Hedgehog signaling negatively regulates WNT signaling, while PTCH1-dependent non-canonical hedgehog signaling positively regulates WNT signaling, contributing to CSCs’ differentiation (Fig. 4i).^537^ *PROX1*, a downstream effector gene of the WNT/β-Catenin signaling pathway, can inhibit each other with Notch1, thereby enhancing the stemness of colorectal cancer cells and hindering their differentiation (Fig. 4j).^426^ Moreover, the SOX9/PROM1 positive feedback loop in colorectal cancer inhibits differentiation by activating the stem cell program, positively correlating with WNT pathway activation and negatively correlating with TGF-β pathway activation (Fig. 4k).^538^ In lung CSCs, the HIF-1ɑ/miR-1275 axis co-activates WNT/β-Catenin and Notch pathways, enhancing stemness and metastatic potential.^539^ In gastric cancer, Chromobox Protein Homolog 7 (CBX7) upregulates the PI3K/AKT pathway, activating the NF-κB pathway to promote miR-21 expression, enhancing the CSC-associated phenotype and metastasis (Fig. 4l).^540^

## Clinical predictive values of CSCs

Using CSC markers, investigators have revealed a negative correlation between the presence of CSCs and patients’ survival in various types of cancers.^541^ Moreover, given the close correlation between CSCs and multi-drug resistance, it is also reasonable to use CSC as a parameter to predict patients’ prognosis after a specific type of anti-cancer treatment.

CSC markers can be used to predict response and survival after chemotherapy. In 47 patients with esophageal squamous cell carcinoma who receive neoadjuvant chemotherapy followed by radical esophagectomy, those with a high pre-chemotherapy expression of CD133 have significantly shorter survival compared to those with low CD133 expression, while the difference in survival is not significant between patients with high CD44 expression and low CD44 expression.^542^ This study also reveals that CD44^high^/CD133^high^ expression is associated with significantly poorer survival compared to those with CD44^low^ or CD44^high^/CD133^low^, suggesting that the combined use of CSC markers can provide better predictive values.^542^ In 112 patients with advanced NSCLC treated with platinum-based chemotherapy, high Nanog levels were independently associated with shorter PFS (hazard ratio (HR) = 3.09, 95% confidence interval (CI) 2.01−4.76) and OS (HR = 3.00, 95% CI 1.98−4.54).^543^ Likewise, overexpression of CXCR4 correlates with poorer PFS and OS in 124 patients with epithelial ovarian cancer receiving cisplatin-based chemotherapy.^544^ Some studies also investigated the predictive value of single-nucleotide polymorphisms (SNPs) of CSC markers, which can influence the transcription, translation, and splicing of these proteins.^545^ For instance, LGR5 rs17109924 is associated with prolonged time to recurrence (TTR) (HR 0.38, 95%CI 0.19−0.79; *P* = 0.006) based on data from 391patients with colon cancer treated with adjuvant 5-fluorouracil-based chemotherapy.^546^ However, this correlation does not occur in patients treated with surgery alone, indicating that the correlation is mainly attributed to the impact of LGR5 rs17109924 on adjuvant chemotherapy.

CSC markers or stemness-related gene signatures also correlate with response to radiation and can be used to predict patients’ prognosis after radiotherapy. A study suggests that the CSC marker CD44 expression can be used to predict local recurrence of larynx cancer based on data from 19 patients.^547,548^ Patients with rectal cancer and high expressions of CSC markers, CD133, OCT4, and SOX2, are prone to develop distant recurrence compared to those with low expressions of the genes.^549^ A systemic review identifies a series of CSC markers, including CD133, CD44, ALDH1, LGR5, and G9a, as indicators for the prognosis of patients with rectal cancer receiving radiotherapy.^550^ Using a machine learning method and data from the TCGA database, a model based on five tumor stemness and immune-related signatures, including Carbamoyl Phosphate Synthetase I (CPS1), CCR2, NT5E, Anillin (ANLN), and ATP-Binding Cassette Sub-Family C Member 2 (ABCC2), demonstrates favorable predictive values predicting radiotherapy responses.^551^ A study suggests that P16^INK4A^ expression is negatively associated with CSC markers and predicts poor survival of patients with cervical cancer after radiotherapy.^552^ GSC markers, CD133 and O6-methylguanine-DNA methyltransferase, are also associated with patients’ responses to radiotherapy.^553^

Some studies attempt to predict patients’ response to or survival after targeted therapy and immunotherapy using CSC markers or stemness-related gene signatures. For instance, a study shows that head and neck squamous cell carcinoma patients with low CD44, a CSC marker, have a significantly better HR for OS than those with high CD44 expression when comparing nimotuzumab plus cisplatin-radiation (NCRT) with cisplatin-radiation (CRT), suggesting that CD44 might be a favorable reference for whether to use nimotuzumab.^554^ Using a five tumor stemness and immune‐specific‐gene (*CPS1*, *CCR2*, *NT5E*, *ANLN*, and *ABCC2*) signature, a study constructs a machine-learning model that can predict therapeutic responses in melanoma patients receiving adoptive T cell therapy (area under ROC curve (AUC) = 0.717) and immune checkpoint blockades (AUC = 0.703).^551^ The AUCs of this signature were higher than those of PD-1 (0.687 for adoptive T cell therapy and 0.505 for immune checkpoint blockades).^551^

## Cancer stem cells and tumor chemotherapy resistance

### The clinical significance of chemotherapy resistance

Chemotherapy remains the cornerstone of current clinical oncology, offering significant reductions in tumor burden and enhanced patient survival, thereby securing its widespread clinical application.^555^ However, patients initially responsive to chemotherapy inevitably evolve into drug resistance. This phenomenon, termed acquired resistance, poses a significant challenge to both clinicians and researchers. Research has progressively revealed that acquired resistance is intricately linked to intratumoral heterogeneity.^300^ Chemotherapy selectively eliminates sensitive subpopulations, allowing resistant cells to prevail and drive disease progression. The contribution of CSCs to intratumoral heterogeneity and acquired resistance has been a focal point of research for decades. This chapter explores the mechanisms through which CSCs mediate resistance and their ramifications for chemotherapy strategies.

### Mechanism of resistance of CSCs to chemotherapy

**Fig. 5:**
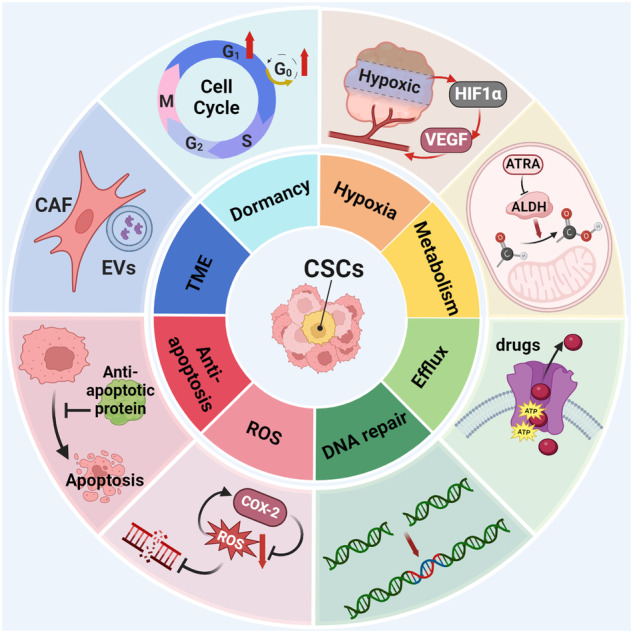
Mechanism of resistance of CSCs to chemotherapy. CSCs possess the ability to maintain a quiescent state and reduce metabolic activity, thereby exhibiting resistance to chemotherapy. Furthermore, CSCs are capable of metabolic reprogramming, utilization of ABC transport proteins, and activation of DNA repair pathways, which allows them to evade chemotherapy. Additionally, the microenvironment plays a crucial role in supporting CSC survival. The balance between ROS and anti-apoptotic versus pro-apoptotic signals, along with exosomes secreted by tumor-associated fibroblasts, dynamically regulates CSCs

In cancer biology, CSCs exhibit dynamic states of proliferation and quiescence. During dormancy, CSCs reduce their metabolic activity, enabling prolonged survival in a quiescent state. Upon extracellular stimulation, however, CSCs may re-enter the cell cycle, regaining proliferative capacity.^556,557^ This duality poses significant challenges for chemotherapy, as quiescent CSCs exhibit resistance to such treatments, and often develop more resistant phenotypes.^558–560^ This resistance is largely attributed to the mechanism of conventional chemotherapy, which targets rapidly dividing cells and acts in a cell cycle-specific manner (Fig. 5).^561^ However, CSCs, with their slow division rates, often residing in the G1 or S phase, exhibit resistance to a variety of chemotherapeutic agents including cisplatin, taxol, and doxorubicin.^562,563^ For instance, the overexpression of Zinc Finger E-Box-Binding Homeobox 2 (ZEB2) increases the proportion of colorectal CSCs in G0/G1 phase, leading to platinum resistance.^564^ Distinguishing quiescent CSCs from proliferative CSCs remains a challenge due to the lack of specific surface markers and common genotypic and phenotypic characteristics.^565^ CD13 has been proposed as a marker for quiescent hepatic CSCs, which have been proven capable of neutralizing chemotherapy-induced ROS and DNA damage.^311^ Moreover, epigenetic modifications also play crucial roles in regulating the quiescent state of CSCs. For example, SET Domain-Containing Protein 4 (SETD4) promotes breast CSC dormancy through the trimethylation of histone H4 lysine 20, facilitating heterochromatin formation.^566^ Elevated levels of miR-135a reduce the methylation at the CG5 site of the *Nanog* promoter by directly targeting DNA Methyltransferases 1 (DNMT1). Then, the combination of SET And MYND Domain Containing 4 (SMYD4) and unmethylated *Nanog* promoter will activate the expression of Nanog in those Nanog-negative tumor cells, thus promoting the switch of CSCs.^567^ Endothelial cells, by expressing miR-126, can induce dormancy in CML stem cells. Concretely, by targeting the PI3K/AKT/mTOR signaling pathway, miR-126 blocks the cell cycle progression of CSCs.^568,569^ Soluble growth factor/receptor pathways, such as CXCL1/CXCL12, Bone Morphogenetic Protein-4 (BMP4), and LIF, have also been shown to regulate the quiescent state of activated CSCs. For instance, CXCL1 induces liver CSC quiescence via mTORC1 kinase activation, while knocking out *CXCL12* downregulates quiescence-associated genes, such as *TGF-β* and *STAT3*, facilitating the exit of leukemia stem cells from dormancy.^570,571^ BMP4 directly regulates the quiescent state of CML leukemia stem cells through a JAK/STAT3 pathway dependent on BMPR1B kinase activity, and the LIF Receptor (LIFR) correlates with the expression of quiescence-associated genes in CSCs, such as *TGFβ2* and *Notch1*. Knockout of *LIFR* promotes the proliferation of breast CSCs and enhances their capacity for bone destruction.^572,573^ More and more evidence suggest the involvement of extracellular vesicles (EVs) in CSC quiescence regulation. EVs from CAFs of hormone therapy-resistant breast cancer patients promote estrogen receptor-independent oxidative phosphorylation and hormone therapy resistance.^574^ Furthermore, CAFs create a resistant niche through close interactions with CSCs, secreting factors like IL-6 and IL-8 that support CSC survival.^575^ In colorectal cancer, CAF-derived EVs trigger resistance to 5-fluorouracil in CSCs, which is the standard of care.^576^ Endothelial cells can promote resistance in GSCs through the secretion of NO, enhancing Notch signaling, or by releasing CD44 ligands.

The HIF pathway emerges as one of the most pivotal regulators of the quiescent state in CSCs.^577^ With the identification of quiescent CSCs in MM, the expression levels of TRIM44 were elevated. This E3 ubiquitin ligase facilitates the deubiquitination and stabilization of HIF-1α under both normoxic and hypoxic conditions, underscoring the intricacy of oxygen sensing in tumorigenesis.^578,579^ Furthermore, the significance of HIF2α in the stability and transformation of CSCs in glioblastoma also highlights the critical role of oxygen levels in CSC biology.^580^ The markers of CSCs such as OCT4, Nanog, SOX2, Krüppel-Like Factor 4 (KLF4), c-Myc, and miR-302 are induced in hypoxic environments further supporting the adaptive responses of CSCs to oxygen deprivation.^581^ Notably, hypoxia not only modulates cell plasticity but also stimulates the proliferation and expansion of pre-existing CSC pools, suggesting a dynamic interplay between CSC quiescence and activation.^582–584^ In conclusion, CSCs exhibit long-term stability and a quiescent phenotype under hypoxia, characterized by low metabolism and reduced oxidative phosphorylation. Conversely, the presence of oxygen triggers the activation of tricarboxylic acid (TCA) cycle enzymes and oxidative phosphorylation, transitioning CSCs into a proliferative state.^585,586^

Metabolic reprogramming stands as one of the hallmarks of the bioenergetics of CSCs.^587^ ALDH enzymes serve as potential inducers of metabolic reprogramming, thereby promoting chemotherapy resistance. For example, ALDH enzymes mitigate aldehyde accumulation by converting them into less toxic carboxylic acids, which play a crucial role in detoxification within CSCs.^588^ Moreover, ALDH enzymes maintain a low level of ROS by consuming these reactive aldehydes induced by ROS.^589^ The increased activity of ALDH1A1 and ALDH3A1 subtypes enables CSCs to metabolize cyclophosphamide and its analogs, such as 4-hydroperoxycyclophosphamide, ifosfamide, and etoposide, and detoxify their intermediate products aldophosphamide into carboxyphosphoramide.^590,591^ ALDH also contributes to the synthesis of retinoic acid and neurotransmitter γ-aminobutyric acid (GABA), essential for the homeostasis and differentiation of CSCs.^330,592^ Inhibition of ALDH activity using all-trans retinoic acid (ATRA) significantly improves prognosis in leukemia, highlighting the enzyme’s involvement in cell differentiation and survival pathways including Notch, mTOR, and PI3K/AKT.^593–595^ The mediating role of ALDH activity in therapy resistance has been established across various cancers, including breast, pancreatic, lung, Ewing’s sarcoma, stomach, glioblastoma, head and neck, ovarian, and colorectal cancers in recent years. Types of chemotherapy agents covered include doxorubicin, paclitaxel, gemcitabine, gefitinib, temozolomide, doxorubicin, and platinum, implicating it as a key marker of CSC drug resistance.^596–604^ Among the 19 ALDH family members, ALDH1 is considered most closely associated with CSCs.^605,606^ However, the complex role of ALDH in CSC biology and therapy resistance warrants further investigation.

The ABC transporter superfamily, encoded within the human genome, represents the largest group of transmembrane proteins. These transporters are categorized into seven subfamilies, ABC-A to ABC-G, based on the similarity or disparity of their domain structures.^607^ Recent studies have revealed a significant upregulation of ABC transporters in CSCs, highlighting their pivotal role in mediating chemotherapeutic resistance by extruding harmful toxins and xenobiotic compounds from cells, thereby reducing intracellular drug concentrations.^83,608^ ABCB1, also known as Multidrug Resistance Protein 1 (MDR1) or P-glycoprotein, was the first member of the ABC transporter family identified in humans.^609^ Wright et al. demonstrated that ABCB1 expression serves as a crucial marker for doxorubicin resistance in breast CSCs.^610^ Various members of the ABC transporter family, especially ABCB1, ABCC1, and ABCG2, are recognized for their heightened expression in CSCs and their involvement in MDR mechanisms.^611–613^ Certain cancers, such as melanoma, might exhibit specific ABC transporter profiles, with ABCB5 playing a significant role.^614,615^ From the structure and properties of ABC transporter, ABCB1, characterized by two ATP-binding sites, exhibits enhanced drug transport capabilities.^616^ Distinct ABC transporters have been implicated in various chemotherapeutic resistances. For example, ABCC1 is primarily associated with resistance to anthracycline drugs, whereas ABCG2 exhibits the broadest spectrum of drug resistance.^617–619^ New roles for ABC transporters in CSCs have been uncovered. ABCB5 possesses the ability to regulate IL-8-dependent CSC maintenance in melanoma and promote the invasion of tumor cells in colorectal cancer. ABCG2 also plays a role in the enhancement of CSC tumorigenic potential.^83,620^ It should be noted that the ABC transporter family is intimately linked with signaling pathways. The *ABCB1* gene promoter contains multiple targets for the β-Catenin complex, suggesting a reciprocal relationship where the WNT/β-Catenin signaling pathway targets ABCB1 activity.^621^ Activation of the WNT/β-Catenin pathway can induce ABCB1 expression, facilitating chemotherapy resistance in CSCs.^622,623^ ABCG2 is also involved in the WNT/β-Catenin signaling cascade.^613,624^ And its expression can be regulated by the Notch pathway as well.^625^ Furthermore, the Hippo pathway effector YAP1 promotes the drug resistance of CSCs through ABCG2.^626,627^ The PI3K/AKT pathway regulates ABCG2 in GSCs at the plasma membrane, instead of the mTOR pathway.^628^ Inhibition of the PI3K/AKT pathway results in the downregulation of ABCG2 in CML cells.^629^ Despite these insights, clinical successes with specific ABC transporter inhibitors remain scarce, underscoring the ongoing need for mechanistic exploration.

Several studies have elucidated that the mechanisms of DNA-damaging chemotherapeutic agents underlying tumor cells, which include DNA crosslinkers (cisplatin, carboplatin, oxaliplatin), DNA synthesis inhibitors (methotrexate), and topoisomerase inhibitors (doxorubicin, daunorubicin).^630^ These agents predominantly target the S phase of tumor cells, where DNA replication occurs, exploiting the diminished DNA repair capacity in tumor cells, which culminates in genomic instability and subsequent apoptosis. Notably, in CSCs, DNA damage checkpoints are activated, facilitating repair mechanisms that enhance cell survival. Sequencing data reveal an upregulation in the majority of DNA damage response and repair genes within CSCs, indicating a superior DNA repair efficiency.^23,631^ The p53 signaling pathway and apoptosis are pivotal to DNA damage repair. Upon detrimental DNA damage, the ATM and Ataxia Telangiectasia And Rad3-Related (ATR) kinase complex with Poly ADP-Ribose Polymerase 1 (PARP-1) and BRCA1, phosphorylate CHK1 and CHK2, thereby activating p53, which leads to cell cycle arrest, DNA repair, or the execution of apoptosis.^632^ Remarkably, genes like *p53*, which induce cell death, often harbor mutations or are dysregulated in CSCs, and inhibiting *p53* aggregation can restore sensitivity to platinum-based treatments.^633^ DNA repair proteins directly or indirectly linked to CSCs’ drug resistance include CHK1, CHK2, ATR, MSI1, RAD50, and RAD51, with RAD51 playing a significant role in resistance to PARP inhibitors.^634–637^

Furthermore, CSCs can prevent DNA damage through effective ROS clearance.^638^ A highly compatible ROS scavenging system has evolved in the CSCs of some tumors to maintain low ROS concentrations. Antioxidant enzymes like superoxide dismutase, glutathione peroxidase, and catalase are markedly active in CSCs.^639–641^ NRF2, a transcription factor, mediates CSC drug resistance by not only regulating the expression of genes involved in the cellular antioxidant response, but also by stimulating drug efflux through raising ATP Binding Cassette Subfamily F Member 2 (ABCF2) expression among other functions.^642,643^ ROS overload or elevated ROS levels induced by chemotherapy have been implicated in the activation of HIFs, which can trigger the activation of pro-survival and developmental pathways such as Notch, WNT, and hedgehog. These pathways in turn contribute to the sustenance of CSCs’ survival.^644^ Additionally, studies have identified a negative feedback loop between ROS and COX-2 within CSCs, where elevated ROS levels induce COX-2 expression, which in turn mitigates ROS accumulation, fostering CSC enrichment and metastasis.^645,646^ Autophagy, a critical biological process for cellular homeostasis, has been recognized as a pivotal resistance mechanism in metastatic prostate CSCs, significantly contributing to ROS scavenging.^647^ To sum up, CSCs exhibit heightened sensitivity to any alteration in the oxidant/antioxidant balance, acquiring resistance under both low and elevated ROS levels.

One of the primary approaches of chemotherapeutic agents is the induction of apoptosis.^648^ The balance between pro-apoptotic (BCL2-Associated X Protein (BAX), BCL2 Antagonist/Killer (BAK), BCL2 Asociated Death Promoter (BAD)) and anti-apoptotic (BCL2, BCL-XL, MCL1) proteins constitutes a focal point of cellular response to apoptosis.^649^ In CSCs with chemotherapy resistance, the balance tips towards anti-apoptotic proteins. It has been shown that compared to tumor cells, CSCs exhibit higher levels of anti-apoptotic gene expression (such as *BCL2*, *BCL-XL*).^650,651^ Knockdown of the biomarkers of CSCs, such as *CD44*, increases apoptosis, evidenced by elevated expression of pro-apoptotic proteins BAX and caspases-3, -8, and -9, while the levels of anti-apoptotic proteins BCL2 and BCL-XL decrease.^652^ Further analysis by Konopleva et al. demonstrated that the overexpression of anti-apoptotic genes *BCL-XL* and *BCL2* could also induce a quiescent state in CSCs.^653^ Moreover, the upregulation of specific cell surface receptors (such as EGFR, Fibroblast Growth Factor Receptor (FGFR), HER2R) in CSCs can inhibit apoptosis by downregulating the pro-apoptotic protein BAD.^654^ Additionally, CSCs can evade apoptosis by prolonging the G2/M phase in the cell cycle through upregulation of G2/M checkpoint proteins CHK1 and CHK2.^650^ Some CSCs overcome apoptosis by upregulating the expression of Inhibitors Of Apoptosis Proteins (IAPs).^655^ The expression of Cellular FLICE-Like Inhibitory Protein (C-FLIP) also affects the apoptosis receptor initiation pathway, inhibiting caspase activation and thereby hindering the apoptotic process. Studies have shown that different splice variants of c-FLIP are associated with resistance to chemotherapeutic drugs.^656,657^ CSCs employ various indirect or direct mechanisms to evade apoptosis, such as endoplasmic reticulum stress. It has been discovered that several components directly involved in endoplasmic reticulum protein processing are dysregulated in CSCs.^658^ In vitro CSC models observed the inactivation of IRE1 (XBP-1 splicing) and the activation of the PERK (elF2α phosphorylation) pathway, both key conduits of the endoplasmic reticulum stress response.^659^ Mitochondrial integrity is crucial for the survival and maintenance of CSCs, with its dysregulation having profound effects on autophagy and apoptosis.^660^ Studies indicate mitochondrial alterations in CSCs of CML compared to normal stem cells. Resistant CSC subpopulations can be identified by higher mitochondrial mass and increased endopeptidase activity.^661^

In conclusion, the chemoresistance mechanisms of CSCs constitute an interactive network (Fig. 5). Targeting individual components may not eliminate the resistance posed by CSCs. To devise accurate CSC-targeted treatments that enhance sensitivity, further exploration in the domain of resistance is warranted.

### Clinical trials targeting CSCs combined with chemotherapy

With a profound understanding of the pivotal role CSCs play in chemotherapy resistance, researchers have initiated a series of targeted clinical trials aimed at exploring potential therapeutic strategies for CSCs (Table 4). These trials broadly fall into three categories. The first category, guided by the ChemoID assay, identifies subsequent treatment regimens using patient biopsy samples before treatment to enrich stem cells and test their response to chemotherapy drugs. Results from a phase III clinical trial, exemplified by NCT03632135, demonstrated a significant reduction in patient mortality risk in the ChemoID assay-guided group, suggesting that the ChemoID assay could become a routine diagnostic and treatment method akin to genetic testing in the future. The second category involves the development of specific inhibitors targeting mechanisms by which CSCs contribute to chemotherapy resistance. This category encompasses most clinical trials, such as those using vismodegib or PF-04449913 to inhibit the hedgehog pathway, LGK974 targeting the WNT pathway, and OMP-52M51 against DLL4 in the Notch pathway. Although γ-secretase inhibitors are the largest group of drugs targeting the Notch pathway, trial outcomes have not been disclosed yet. Additionally, the development of the drugs RO4929097 and PF-03084014 has been halted for various reasons. NCT04137627 evaluated melatonin as an antioxidant in combination with neoadjuvant chemotherapy for changes in tumor stemness expression in oral squamous cell carcinoma, but results showed no statistical difference despite a reduction in miR-210 and CD44 expression, implying the tumor microenvironment might play a role in CSC resistance mechanisms but may not be the dominant factor. In theory, PARP inhibitors involved in DNA repair could also be effective against CSCs, but current clinical trials have not measured changes in CSCs or biomarkers before and after treatment, necessitating further exploration of their effect on CSCs. The third category targets markers specific to CSCs for treatment, such as CD44v6. Bivatuzumab mertansine, an antibody-drug conjugate (ADC) targeting CD44v6, enhances the specificity of chemotherapy and has shown promising results in various cancer treatments. However, its effectiveness against CSCs, as indicated by NCT02254005, remains inconclusive. These results indicate that we cannot yet conclusively determine whether targeting CSCs can reverse chemotherapy resistance. We look forward to the anticipated outcomes of these clinical trials in the coming years.

**Table 4:** Clinical studies on combination of chemotherapy and CSC-targeting therapies

Target	NCT Number	Tumor type	Phases	Conditions	Enrollment	Interventions	Study Results	Study Status
Antioxidant	NCT04137627	Oral squamous cell carcinoma	III	Locally advanced oral squamous cell carcinoma	50	Melatonin plus neoadjuvant chemotherapy	Melatonin to neoadjuvant chemotherapy reduced the expression of miR-210 and CD44 and the percentage of tumor residue	Completed
CD44v6	NCT02254005	Breast cancer	I	CD44v6 positive metastatic breast cancer	24	Bivatuzumab mertansine	NA	Completed
DLL4	NCT01189968	Lung cancer	I	Non-squamous non-small cell lung cancer	50	Demcizumab	NA	Completed
DLL4	NCT01778439	Solid tumors	I	Relapsed or refractory solid tumors	48	OMP-52M51	2 had unconfirmed partial response and 4 subjects had prolonged (≥6 months) disease stabilization	Completed
Hedgehog pathway	NCT01195415	Pancreatic cancer	II	Metastatic or recurrent disease following surgical therapy	25	Vismodegib plus gemcitabine hydrochloride	No significant changes were detected in CSCs pre- and postbiopsy. The median PFS and OS for all treated patients were 2.8 and 5.3 months. The response and disease control rate was 21.7% and 65.2%.	Completed
Hedgehog pathway	NCT00607724	Solid tumors	I	Locally advanced or metastatic solid tumors	68	Vismodegib	Of the 33 patients, 18 had an objective response to GDC-0449	Completed
Hedgehog pathway	NCT01204073	Solid tumors	I	Advanced nonhematologic malignancies	34	TAK-441	NA	Completed
Hedgehog pathway	NCT01286467	Solid tumors	I	Advanced/metastatic solid tumor	23	PF-04449913	Eight patients (34.8%) achieved stable disease; none had complete or partial response. Three patients with disease progression at enrollment had prolonged disease stabilization (≥6 months).	Completed
Hedgehog pathway	NCT01255800	Head and neck cancer	I	Recurrent head and neck cancer	9	IPI-926 and cetuximab	NA	Completed
NA	NCT03949283	Ovarian cancer	III	Platinum-resistant recurrent ovarian cancer	150	Standard chemotherapy	Physician choice treatment: PFS 12.0 mos; OS 15.0 mos ChemoID-guided treatment: PFS: 3.5 mos; OS: 6.0 mos	Completed
NA	NCT03632798	Ovarian cancer	III	Recurrent ovarian cancer	300	Bevacizumab plus standard chemotherapy	NA	Suspended
NA	NCT02423811	Esophageal squamous cell carcinoma	II	Newly diagnosed stage II and III esophageal squamous cell carcinoma	20	Fursultiamine plus concurrent chemoradiotherapy	NA	Completed
NA	NCT01777919	Glioblastoma	II	Newly diagnosed glioblastoma multiform	32	Temozolomide plus disulfiram/copper	NA	Unknown
NA	NCT03632135	Glioblastoma	III	Recurrent glioblastoma	78	Standard chemotherapy	ChemoID assay-guided group, median survival is 12.5 months compared with 9 months in the physician-choice group as per interim efficacy analysis. ChemoID assay-guided group has a significantly lower risk of death	Completed
VEGF/DLL4	NCT03030287	Ovarian, peritoneal or fallopian tube cancer	I	Platinum resistant ovarian, primary peritoneal or fallopian tube cancer	44	OMP-305B83 plus paclitaxel	ORR: 43.2%; 33.3% in patients previously treated with bevacizumab, 64.3% in bevacizumab-naive patients, and 62% in biomarker-positive patients. The mDOR was 6 months	Completed
WNT pathway	NCT01351103	Solid tumors	I	Tumors of any histological origin with genetic alterations upstream in the WNT signaling pathway	185	LGK974	16% of patients had stable disease	Active, not recruiting
WNT pathway	NCT01398462	Acute myeloid leukemia	I	Acute myeloid leukemia	69	CWP232291	NA	Completed
Notch Pathway	NCT00645333	Breast cancer	I/II	Advanced or metastatic breast cancer	30	MK-0752 plus docetaxel plus pegfilgrastim	NA	Completed
Notch Pathway	NCT00878189	Leukemia	I	T-cell acute lymphoblastic leukemia and T-cell lymphoblastic lymphoma	72	PF-03084014	NA	Completed
Notch Pathway	NCT01149356	Breast cancer	I	Advanced or metastatic breast cancer	15	RO4929097 plus exemestane + goserelin acetate vs exemestane + goserelin acetate	NA	Terminated
Notch Pathway	NCT01876251	Breast cancer	I	Advanced breast cancer	30	PF-03084014 plus Docetaxel	NA	Terminated
Notch Pathway	NCT01154452	Sarcoma	I/II	Sarcoma	78	RO4929097	NA	Completed
Notch pathway	NCT01192763	Pancreatic cancer	I	Pancreatic cancer	30	Neoadjuvant RO4929097	NA	Terminated

## Cancer stem cells and tumor immunotherapy resistance

### CSCs and immune evasion

Immune cells within the tumor microenvironment play a pivotal role throughout the oncogenesis and progression of tumors. Unlike their counterparts in normal tissues, these immune cells often exhibit attenuated inflammatory responses or enhanced suppressive functions, thereby facilitating tumor immune evasion. This section aims to provide an overview of the principal roles played by TAMs, MDSCs, NK cells, T cells, and B cells in mediating immune escape of CSCs. Their complex interplay and the mechanisms through which they contribute to the immunological cloak that shields CSCs from the host’s immune defense are critical to understanding and developing novel therapeutic strategies.

Research across various tumor types has revealed that TAMs often constitute up to 50% of the immune cell population, positioning them at one of the forefront research fields of immune cells within the microenvironment.^662^ The heterogeneity of TAMs emerges as a critical mechanism behind immunotherapy resistance. Engagement of damage-associated molecular patterns (DAMPs) with specific pattern recognition receptors on macrophages, such as TLR4, triggers pro-inflammatory signaling and polarization towards the M1 phenotype.^663^ M1-TAMs, characterized as classically activated macrophages, exhibit enhanced pathogen phagocytosis capabilities, thereby exerting anti-tumoral properties. However, the tumor microenvironment promotes polarization towards the M2-TAMs, which possess pro-tumoral potential, supporting and sustaining CSCs and therapy resistance through the secretion of chemokines and activation of stemness pathways, such as sonic hedgehog ligands. For instance, GSCs can recruit M2-TAMs by secreting periostin.^664^ CSCs may also elevate M2–TAM levels through chemokines like CCL2 and macrophage colony-stimulating factor 1 (CSF1).^665^ Drug-resistant lung CSCs activate the Interferon Regulatory Factor 5 (IRF5)/M-CSF pathway to promote the production of M2-TAMs from CD14^+^ monocytes.^666^ In turn, M2-TAMs secrete factors like milk fat globule-EGF factor 8 (MFG-E8), activating STAT3 and sonic hedgehog signaling in CSCs, thereby enhancing treatment resistance.^667^ Moreover, TAMs secrete substantial amounts of TGF-β1, maintaining CSC characteristics and promoting EMT.^257,668^ Lu et al. demonstrated that CSCs undergoing EMT upregulate CD90/Thy1 and EphA4, crucial proteins mediating physical interactions between CSCs and TAMs. EphA4 receptor activation secretes Src and NF-κB, inducing CSCs to secrete various cytokines maintaining stem cell status.^282^ ScRNA-seq has clarified the bidirectional feedback mechanisms between CSCs and TAMs. CSCs secrete S100A11 protein to promote TAM polarization towards the M2 phenotype, which in turn enhances CSC self-renewal and metastatic capabilities.^247^ The crosstalk between CSCs and macrophages is intricate, wherein CSCs not only polarize macrophages towards a tumorigenic state but also employ protective mechanisms to avoid macrophage phagocytosis. Elevated expression of CD47, observed in CSCs from both hematological malignancies like AML and solid tumors like pancreatic, liver, and lung cancers, interacts with Signal Regulatory Protein α (SIRPα) on TAMs, broadcasting a “don’t eat me” signal to protect them from macrophage engulfment.^669–674^ Recent studies have also identified TAMs as “iron donors” within the tumor microenvironment, fulfilling the high iron demand of CSCs and playing a crucial role in influencing iron homeostasis.^675^ Although the mediators involved in this crosstalk may vary with tumor pathology, such interactions may one day become potential therapeutic targets against CSCs.

Tumor-infiltrating myeloid cells represent a heterogeneous lineage that includes TAMs, MDSCs, and so on, the latter being a focal point of research due to their impact on limiting the efficacy of immunotherapy.^676^ MDSCs, immature myeloid cells derived from the bone marrow, are categorized into polymorphonuclear MDSCs (PMN-MDSCs) and monocytic MDSCs (M-MDSCs), expressing CD15 and CD14, respectively.^677,678^ These cells exert immunosuppressive effects through distinct mechanisms, with the ratio of PMN-MDSCs to M-MDSCs in peripheral blood being crucial.^679^ In murine models of melanoma, prostate, and cervical cancers, CSCs promote PMN-MDSC infiltration by overexpressing G-CSF, CXCL5, and TGFβ.^680–682^ In turn, PMN-MDSCs increase STAT3 phosphorylation, CD133 and CD44 expression, and sphere formation of colorectal CSCs in vitro by secreting S100A9 protein.^210^ PMN-MDSCs also enhance the ratio of CSCs, spheroid-forming ability, and expression of stemness-related genes in myeloma cells by inducing piRNA-823 expression.^286^ Moreover, studies have identified M-MDSCs as primary drivers of the CSC phenotype in pancreatic and breast cancers.^288,290^ In breast tumor models, M-MDSCs comprise the majority of tumor-infiltrating MDSCs. Mechanistic analysis has shown that NO produced by M-MDSCs promotes the CSC phenotype through activation of Notch signaling and sustained STAT3 phosphorylation in cancer.^288,677^ The relationship between CSCs and MDSCs is bidirectional, as CSCs also recruit MDSCs to limit T cell activity, creating a favorable environment for tumor growth. MDSCs in peripheral lymphoid organs are predominantly PMN-MDSCs, which exhibit relatively mild immunosuppressive activity compared to M-MDSCs. PMN-MDSCs primarily produce high levels of ROS to exhibit immunosuppressive activity, which are unstable and transiently, requiring antigen-specific interactions with T cells to ultimately induce tumor-specific T cell tolerance.^683^ In contrast, M-MDSCs produce substantial amounts of NO, arginase 1, and immunosuppressive cytokines with longer half-lives, effectively inhibiting nonspecific T cell responses without the need for direct contact between MDSCs and T cells.^679^ It is noteworthy, however, that despite functional annotation and transcriptomic profiles widely recognizing PMN-MDSCs as distinct from inflammatory neutrophils, tumor-associated neutrophils, and PMN-MDSCs share overlaps in markers and suppressive functions, suggesting a close phenotypic and functional relationship.^684^

T cells are the most pivotal immune effector cells in the anti-tumor response, executing cytotoxic effects on tumor cells through classical pathways such as perforin/granzyme release, death receptor engagement, and induction of apoptosis.^685^ Studies have revealed that CSCs evade T cell-mediated immune rejection by downregulating key components of the antigen processing and presentation machinery and suppressing T cell anti-tumor functionality.^686^ Tumor antigens are broadly classified into two categories: (1) tumor-specific antigens (TSAs), encoded by mutated or rearranged genes, and (2) tumor-associated antigens (TAAs), encoded by genes specific to the normal cellular lineage.^687^ CSCs may selectively avoid expressing differentiation-related TAAs, thus resisting T cell-mediated rejection.^688,689^ Another mechanism of immune evasion involves the downregulation or loss of MHC-I by CSCs.^690^ As MHC-I play a crucial role in immune recognition, their absence or reduced expression can limit T cell-mediated lysis of CSCs.^689,691^ The induction of T cell tolerance by CSCs is another key strategy in escaping immune surveillance. A primary mechanism of tolerance induction involves the clonal deletion of antigen-reactive T cells through apoptosis or death, with the Factor-Related Apoptosis (Fas)/Fas-L pathway serving as a significant mediator.^692,693^ CSCs may actively destroy T cells through the expression of Fas-L, moreover, the autocrine secretion of soluble Fas-L protects CSCs from cytotoxic T cell-mediated Fas killing.^693–695^ Furthermore, CSCs can evade immune attack by downregulating Fas.^693^ The tumor-expressed ligand Receptor-Binding Cancer Antigen Expressed On SiSo Cells (RCAS1) has also been found to induce apoptosis in T, B, and NK cells expressing its receptor.^696^ Immunogenic tolerance can be achieved through non-deletional processes, such as the inactivation of antigen-reactive cells.^697^ The secretion of TGF-β underpins the inhibition of T cell proliferation mediated by MSCs.^698^ CSCs produce TGF-β and IL-10, directly suppressing T cells to avoid immune-mediated destruction.^699–701^ The TGF-β signaling pathway is also specifically activated in CSCs, with secreted morphogens of the TGF-β superfamily and their receptors preferentially expressed by CSCs.^702–704^ T cell activation in the immune response requires two signals.^705^ The first signal comes from the T Cell Receptor (TCR) recognizing the MHC/antigen peptide complex, conveying an antigen-specific recognition signal.^706^ The second signal is provided by co-stimulatory molecules of antigen-presenting cells (APCs), offering a non-specific synergistic co-stimulation signal.^707^ CSCs may reduce T cell responsiveness to tumor antigens by actively modulating the activation state of APCs and may express negative co-stimulatory molecules to disrupt anti-tumor immune responses.^708,709^ PD-1/PD-L1-mediated negative co-stimulatory signal transduction is the most common way of inhibiting lymphocyte activation.^710,711^ Other mechanisms include the induction or active recruitment of regulatory T (Treg) cells, which can effectively suppress the activation, proliferation, and cytokine production of other T cells, crucial for maintaining immune self-tolerance and homeostasis.^712–715^ In summary, CSCs employ a multitude of processes to drive tumor escape from immune-mediated rejection responses.

In the era of immune checkpoint inhibitors (ICIs) and adoptive T cell therapies, the pivotal role of T cells in anti-tumor immunity has become indisputable. However, these advancements have also exposed numerous limitations of T cells, underscoring the urgent need to unravel immunological mechanisms. With the advancement of scRNA-seq technology, the subpopulations and states of B cells within the tumor microenvironment are increasingly scrutinized. For instance, in melanoma, genes associated with early B cell stages are extensively expressed.^716^ In breast cancer, B cells predominantly exist as naive B cells, memory B cells, with fewer plasma cells and germinal center cell clusters observed. Notably, compared to peripheral blood B cells, tumor-associated B cells exhibit higher levels of somatic mutations and greater clonal expansion.^717,718^ Another distinctive function of B cells was observed in ovarian cancer, where B cells preferentially express IgA, while in breast cancer, B cells mainly express IgM and IgG. This IgA can target antigens and be internalized by tumor cells in an antigen-independent manner through Polymeric Immunoglobulin Receptor (PIGR), sensitizing tumors to T cell.^719^ Current research indicates that the states of tumor tissue-associated B cells vary across different types of tumors, but largely remain in a pre-antibody class-switched state. Some studies suggest that in the presence of ongoing tumors, exhausted or dysfunctional CD8^+^ and CD4^+^ T cells seek the aid of B cells in the microenvironment, through the expression of CXCL13, to form tertiary lymphoid structures (TLS).^720–722^ Research across multiple cancers demonstrates that the presence of TLS and B cells in tumor tissues correlates with better prognoses, and the anti-tumor efficacy of T cells is enhanced in the presence of B cells.^723–726^ Interestingly, TLS can also be exploited by tumor cells under certain conditions to promote lymphatic infiltration of tumor cells, leading to lymphatic metastasis.^727^ However, few studies have revealed a direct significant correlation between the CSC phenotype and both TLS and B cells.^728^ Tumors with low TLS infiltration may present higher CSC characteristics, with increased proliferation and metastatic potential.^729^ In summary, the presence of TLS is considered a crucial component of anti-tumor immunity. With the development of scRNA-seq and spatial transcriptomics, research into the functions of TLS within tumors and their relationship with CSCs is expected to mature and refine further.

NK cells represent a crucial component of the innate immune system, constituting the third major lymphocyte type, following T cells and B cells. They play a complementary role to T cells by eliminating reduced or absent MHC class I expression tumor cells which evade CD8^+^ T cell detection, and can also recruit dendritic cells to indirectly enhance T cell-mediated responses.^730^ Emerging evidence suggests that CSCs may be particularly susceptible to NK cell-mediated targeting. In colorectal cancer models, CSCs exhibit increased vulnerability to NK cell cytotoxicity, associated with the upregulation of natural cytotoxicity receptors, especially NKp30 and NKp44.^731^ Intriguingly, GSCs demonstrate resistance to unstimulated NK cells but exhibit heightened sensitivity in co-culture models following pre-treatment with IL-2 and IL-15.^732^ This preferential susceptibility might be mediated by increased expression of Natural-Killer Group 2 Member D (NKG2D) ligands UL16 Binding Protein 1 (ULBP1), ULBP2, and MHC Class I Chain-Related Protein A (MICA) on CSCs.^733^ Beyond their capacity to directly eliminate CSCs, NK cells can also induce their differentiation. In the presence of CSCs and IL-2, the cytotoxicity of NK cells is suppressed, and cytokine production is enhanced, a state referred to as “split energy”.^734^ These split anergic NK cells secrete high levels of Interferon-γ (IFN-γ), which induces the expression of MHC-I, differentiation receptors, and PD-L1 while reducing CD44 levels on CSCs. This induction of CSC differentiation subsequently leads to slowed tumor growth and decreased metastatic spread.^735^ Therefore, NK cells appear to counter tumor progression through a dual-step mechanism: initially eliminating a portion of CSCs and then, following a phase of split energy inducing cellular differentiation within the remaining CSC population.^736^ However, the local tumor microenvironment can directly inhibit NK cell effector mechanisms. Tregs suppress NK cell functions in a TGF-β-dependent manner, while CAFs inhibit NK cell functions through cell-cell communication and the release of PGE2.^737–739^ CSCs can also impede NK immune responses through various inhibitory mechanisms. In metastatic melanoma, the expression of Indoleamine-2,3-Dioxygenase (IDO) and/or production of PGE2 can modulate the expression of NKp30, NKp44, and NKG2D.^740^ In neuroblastoma, TGF-β suppresses NK cell functions by regulating the expression of activation receptors and chemokine receptor repertoires, chiefly interfering with their migration and accumulation within tumor nests.^741,742^ In ovarian tumors, the expression of Macrophage Migration Inhibitory Factor (MIF) and the glycoprotein MUC-16 can downregulate NKG2D and disrupt the formation of synapses between tumor cells and NK cells.^743,744^ Additionally, CSCs evade immune surveillance and reduce NK cell-mediated killing by actively shedding MICA and MICB and recruiting Tregs.^745,746^ Kryczek et al. observed that IL-22 promotes the CSC phenotype in preclinical and patient-derived models, with IL-22 being produced by NK and T cells.^747^

In summary, immune cells’ fight against tumors mainly goes through three stages: immune elimination, immune equilibrium, and immune evasion. In the initial phase, T cells and NK cells identify and eradicate proliferating CSCs before they develop into full-blown cancer. Consequently, CSCs with high immunogenicity are gradually eliminated by the immune system, such as high MHC-I or NKG2D, leaving behind those with low immunogenicity or those in a quiescent state to survive into the second phase. Ultimately, these selected CSCs expand uncontrollably with the help of immunosuppressive effectors, and the immune system becomes incapable of suppressing them.

### Immunotherapy targets cancer stem cells

Tumor immunotherapy represents a therapeutic approach that harnesses the immune system to generate tumor-specific immune responses, aimed at suppressing and eliminating tumor cells. Based on the different mechanisms of the immune response against tumors, tumor immunotherapy can be categorized into “active immunotherapy” and “passive immunotherapy”.^748^

The core of passive immunotherapy hinges on administering immune effectors with antitumor activity, such as tumor-specific T cells and antibodies. This method offers rapid action but fails to elicit a lasting immune response.^748^ Tumor-specific monoclonal antibodies (mAbs) represent the most well-known form of immunotherapy, widely utilized in clinical practice.^749^ The mechanisms for mAbs primarily encompass (1) specific recognition of molecules expressed on tumor cell surfaces, leading to tumor cell death via phagocytosis, complement system activation, and antibody-dependent cell-mediated cytotoxicity (ADCC); (2) disruption of signaling pathways essential for tumor cell progression and survival, or inducing death signals by binding to surface receptors; and (3) conjugation with cytotoxic drugs or radioactive isotopes for specific delivery to tumors.^750^ Adoptive cell immunotherapy (ACI or AIT) involves infusing immune cells with anticancer activity back into the patient. This includes chimeric antigen receptor T-cell (CAR-T) therapy, tumor-infiltrating lymphocytes (TILs) therapy, NK cell therapy, and cytokine-induced killer (CIK) cell therapy.^751,752^ The principle behind these therapies is the isolation of immune cells with cytotoxic potential from the patient. CAR-T therapy involves genetically engineering isolated T cells to bind tumor cell antigens;^753,754^ CIK cells, expressing both CD3 and CD56 membrane proteins, are a novel type of immune cell known as NK-like T lymphocytes with potent anticancer activity.^755,756^ In contrast to passive immunotherapy, active immunotherapy only exerts anticancer effects after activating the host’s immune system. Initial attempts to enhance antitumor immunity relied on non-specific immune stimulation, such as the local administration of inflammatory molecules (such as pathogen-associated molecular patterns (PAMPs) and DAMPs) and immunostimulatory cytokines (such as G-CSF, GM-CSF, TNF-α, IFN-α, IL-2).^757^ Unlike non-specific immunostimulants, antitumor vaccine inoculation offers high tumor specificity.^758^ Notably, immunomodulatory mAbs, such as ICIs, fall under active immunotherapy. These drugs activate new or restore pre-existing host immune responses by blocking the interactions between tumor cells expressing immune checkpoints and immune cells.

**Fig. 6:**
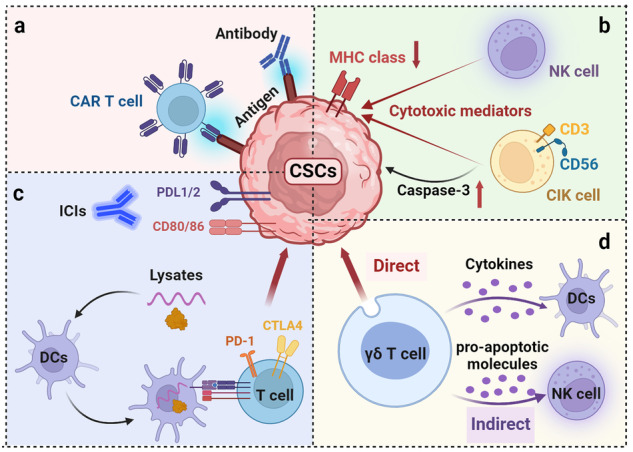
Immunotherapy targets CSCs. **a** Targeted therapy using antigens of CSCs, such as CAR-T and monoclonal antibodies, etc. **b** Leverage the innate immune cells’ natural cytotoxic activity to circumvent antigen presentation and nonspecifically target CSCs, such as NK cells or CIK cells. **c** Active immunization strategies involve the use of DC vaccines loaded with CSC lysates, or the reinvigoration of T cells through targeting immune checkpoints. **d** γδ T cells exhibit the dual capacity to directly attack CSCs and indirectly stimulate NK cells or DCs to target CSCs

Over the past two decades, mAbs have emerged as effective therapeutic agents for cancers, significantly enhancing the survival rates and quality of life.^759^ CSCs can be identified by combinations of positive and negative expression of surface markers, with novel mAbs becoming increasingly potent and specific drugs targeting CSCs (Fig. 6a). Among these, CD44 has been recognized as one of the well-known CSC markers, playing a crucial role in EMT as well as in the initiation, progression, and metastasis of tumors.^760,761^ RG7356, an anti-CD44 mAb, has shown promise in preclinical models, demonstrating activation of macrophages and good tolerance in both solid and hematological malignancies.^762–764^ However, the ADC targeting CD44v6, bivatuzumab mertansine, was prematurely discontinued due to life-threatening off-target skin toxicity.^765^ Other CSC markers that have entered clinical trials include CD24, CD47, CD123, EpCAM, CD9 and so on.^759,766–769^ However, only CD105-targeting crituximaband EpCAM-targeting edrecolomab have entered phase III clinical trials.^770,771^ Beyond ADCs, bispecific antibodies, which target two different CSC antigens simultaneously, have shown superior efficacy in preclinical models compared to agents targeting a single antigen.^772^ However, the clinical trial success of these bispecific antibodies remains to be further observed.

Another promising therapeutic strategy targeting CSCs is CAR-T cell therapy (Fig. 6a).^773^ This approach holds a distinct advantage over TILs therapy or ex vivo activation of autologous unmodified T cells, as CSCs often exhibit reduced antigen presentation capabilities due to the downregulation of MHC and/or antigen-processing machinery (APM) molecules. While, tumor cell recognition by CAR-T cells without relying on the MHC complex.^774,775^ Various CAR-T cell therapies have been developed for GSCs. For instance, research by Zhu et al. demonstrated that CAR-T cells targeting CD133 effectively kill CD133^+^ CSCs in glioma patients, both in vitro and in vivo.^776^ However, such therapies have not succeeded in completely eradicating tumors, possibly due to tumor cell-induced terminal differentiation or senescence of CAR-T cells. When CD57^+^ glioma cells interact with CAR-T cells, an increase in the expression of the T cell senescence marker CD57 on CAR-T cells is observed.^776^ Similarly, CAR-T cells targeting the epidermal growth factor receptor variant III (EGFRvIII) have been effective in killing target cells. EGFRvIII has been identified as a tumor-specific antigen for GSCs.^777^ Yet, in a phase I clinical trial for glioblastoma patients, EGFRvIII-targeted CAR-T cells induced downregulation of tumor antigens and significant upregulation of inhibitory molecules.^778^ These findings underscore the need for further efforts to enhance the efficacy of CAR-T cell therapy. In recent years, numerous CAR-T therapies targeting antigens associated with CSCs, including CD22, CD123, and ALDH, etc., have been developed. CAR-T therapy remains a leading trend in future research endeavors.

As previously mentioned, most normal cells expressing MHC-I molecules are not targeted by NK cells. However, tumor cells and CSCs that downregulate MHC-I molecules while upregulating activating ligands become primary targets for NK cell-mediated cytotoxicity.^779,780^ The imbalance in the expression of MHC-I and NK activating ligands on CSCs leads to increased sensitivity to NK cell killing.^781^ This pattern of NK ligand expression and sensitivity to its cytotoxic effects has been reported across multiple tumor types, including gliomas, colorectal cancer, melanoma, pancreatic cancer, oral squamous cell carcinoma, breast cancer, and Ewing’s sarcoma.^732–734,782–784^ Nonetheless, multiple studies have also shown that the targeting capability of NK cells can be influenced by the tumor microenvironment, underscoring the need for further research to identify the appropriate subtypes of NK cells as carriers and to effectively target and kill tumors (Fig. 6b).^740,785,786^

In recent years, γδ T cells, a subset of non-conventional T cells characterized by their expression of heterodimeric T-cell receptors (comprising γ and δ chains) and their non-restrictive antigen recognition, have garnered significant interest within immunotherapy.^787^ Present in the immune infiltration of human cancers, γδ T lymphocytes have been shown to play a role in antitumor immune responses.^788^ Specifically, Vγ9Vδ2 T cells have demonstrated the capability to kill various tumor cells in vitro and in vivo, independent of the tumor cells’ MHC molecule expression levels.^789^ The antitumor activity of Vγ9Vδ2 T cells is exerted through two main mechanisms: direct induction of cytotoxic mechanisms akin to those of CD8^+^ T cells and indirect stimulation of other immune cells such as NK cells and cytotoxic T lymphocytes (CTLs).^790,791^ Notably, the activation of Vγ9Vδ2 T cells can be induced by bisphosphonates, drugs associated with bone metastasis.^792^ Treatment of CSCs with zoledronic acid stimulates Vγ9Vδ2 T cells to secrete cytokines like IFN-γ, express pro-apoptotic molecules such as TNF-Related Apoptosis-Inducing Ligand (TRAIL), and release cytotoxic granules, ultimately inducing CSC death through a TCR-dependent mechanism (Fig. 6d).^793–795^ Moreover, chemotherapy drugs like doxorubicin and 5-fluorouracil can induce the expression of TRAIL and NKG2D activating ligands on CSCs, rendering them sensitive to Vγ9Vδ2 T cell-mediated killing.^796^ Vγ9Vδ2 T cells can also enhance the chemosensitivity of ovarian CSCs by reducing the expression of multidrug resistance components ABCG2, topoisomerase 2a, and 2b.^797^ Clinical trials involving Vγ9Vδ2 T cells have been conducted in various tumors, including breast cancer, prostate cancer, lung cancer, and head and neck cancer.^788^ Although these therapies can reduce tumor burden, only modest improvements in long-term survival rates have been observed, highlighting the importance of further research into mechanisms regulating CSC sensitivity to γδ T cells and considering these mechanisms in the design of new clinical trials.

Additionally, CIK cells have demonstrated the ability to kill CSCs in preclinical models of melanoma, sarcoma, and liver cancer (Fig. 6b).^798,799^ In liver CSCs, CIK cells induce caspase-3-dependent apoptosis and G2/M arrest.^800^ In melanoma and sarcoma, CIK cells exert direct cytotoxic effects.^798,799^ CIK cells emerge as promising candidates for targeting CSCs in immunotherapy for two main reasons: their cost-effectiveness compared to other immune cell populations and their sensitivity to CSCs resistant to chemotherapy and targeted therapies, with easy derivation from patients who have undergone these treatments.^801^ Therefore, combining CIK therapy with chemotherapy or molecular-targeted therapies may represent a future direction for immunotherapy.

Dendritic cells (DCs)-based antitumor vaccines, a widely applied immunotherapeutic strategy targeting CSCs, primarily operate by loading DCs with proteins or mRNA from tumor lysates, thereby activating specific tumor immune responses (Fig. 6c).^802^ The nature of the antigen (such as peptides, whole proteins, or mRNA) impacts the resultant immune response, with whole proteins capable of activating both CD8^+^ and CD4^+^ T cells, whereas mRNA encoding antigens induces only CD8^+^ T cell responses.^803^ Pellegatta et al. pioneered the construction of DC vaccines using lysates from GSCs, demonstrating that CSC-based DC vaccines exhibit higher efficacy compared to non-CSC-based DC vaccines (utilizing glioma cells).^804^ Moreover, therapeutic tumor vaccines, as adjunct therapy post-radiotherapy or surgical resection, show more potential benefits than prophylactic vaccination. Qiao et al.‘s series of studies confirmed that adjuvant therapy with DC vaccines based on ALDH^+^ cells significantly reduces local tumor recurrence, inhibits spontaneous lung metastasis, and prolongs host survival in lung cancer or melanoma patients, outcomes not achieved with DCs loaded with non-CSCs or an unselected cancer cell population.^805,806^ This advantage likely stems from the CSC-specific humoral and cellular immune responses generated by DCs loaded with CSCs.^805,806^ These encouraging preclinical results have propelled CSC-loaded DC vaccines into the clinical application phase. The first clinical trial of a CSC-loaded DC vaccine in glioblastoma patients, although limited to seven patients, reported extended PFS compared to historical controls.^807^ Additional clinical trials in lung and pancreatic cancer patients have not shown significant adverse side effects, confirming the safety of CSC-targeted DC vaccines.^808,809^ However, it is noteworthy that these clinical studies did not compare outcomes with DC vaccines loaded with non-CSCs or unsorted cells, leaving the replicability of preclinical success in humans in question. Furthermore, while CSC-targeted vaccines offer a cost-effective advantage over other immunotherapies, they may increase economic burdens.

Immune checkpoints such as PD-L1 play a crucial role in the AKT signaling pathway, impacting the expression of embryonic stem cell transcription factors OCT4A, Nanog, and the stem cell factor BMI1.^495^ Concurrently, the downregulation of PD-L1 impairs the self-renewal capabilities of breast CSCs. Interaction between PD-L1 and PD-1 enhances the proliferative capacity of gastric cancer stem-like cells.^810^ Similarly, CTLA-4 exhibits analogous functions. ALDH^+^ melanoma stem cells express CTLA-4, indicating its ability to support cellular proliferation and inhibit apoptosis in vitro. Blocking CTLA-4 can suppress both in vitro and in vivo self-renewal and tumorigenic capabilities by depleting ALDH^+^ cells.^811^ Consequently, in CSC-targeted therapy, the application of ICIs holds particular appeal. Preclinical studies have demonstrated that anti-CSC vaccines combined with anti-PD-L1 therapy, as adjuvant treatment following surgical resection of squamous cell carcinoma, significantly inhibit tumor recurrence and prolong survival compared to monotherapy.^806^ Moreover, a triple regimen combining anti-PD-L1 with anti-CTLA-4 and an anti-CSC vaccine is more effective in promoting tumor regression in melanoma-bearing mice than the anti-CSC vaccine alone.^812^ These antitumor effects are attributed to the significant depletion of ALDH^+^ CSCs following combination therapy, associated with T cell expansion, suppression of TGF-β secretion, increased IFN-γ secretion, and notably enhanced host-specific CD8^+^ T cell responses against CSCs.^813^ Researchers conclude that combining anti-CSC vaccines with PD-1 blockade can enhance the functionality of tumor-specific CTLs and protect mice from secondary challenges by CSCs.^813^

### Clinical trials of targeting CSCs combined with immunotherapy

In recent years, vaccination against CSCs has garnered increasing attention in the clinical research domain (Table 5). These trials encompass various cancer types, including pancreatic cancer (NCT02074046), nasopharyngeal carcinoma (NCT02115958), breast cancer (NCT02063893), hepatocellular carcinoma (NCT02089919), lung cancer (NCT02084823), colorectal cancer (NCT02176746), and ovarian cancer (NCT02178670). Although these trials are listed as completed on ClinicalTrials.gov, the research outcomes have yet to be reported. On another front, with the advancement of CAR-T cell technology, an increasing number of clinical trials are exploring CAR-T cell therapies using CSC biomarkers. These cells can bypass the antigen presentation process and directly target CSCs, exerting anti-tumor effects. NCT02541370, a single-arm phase II trial, demonstrated promising anti-tumor activity and manageable safety for CD133-targeted CAR-T cells in advanced hepatocellular carcinoma. Additionally, Catumaxomab, a bispecific antibody targeting EpCAM and CD3, has been proven effective in eliminating malignant ascites in several clinical trials. However, due to its high cost and potential adverse reactions from targeting CD3, the drug was withdrawn from the market in 2017. While therapies like ICIs and NK cells have shown some efficacy in combating tumors, they struggle to effectively distinguish between tumor cells and CSCs. Hence, despite the promising strategy of employing immunotherapy to target CSCs, further research is crucial for its broader clinical application.

**Table 5:** Clinical studies on combination of immunotherapy and CSC-targeting therapies

Target	NCT Number	Tumor type	Phases	Conditions	Enrollment	Interventions	Study Results	Study Status
CD123	NCT04272125	Acute myeloid leukemia	I/II	Refractory or relapsed acute myeloid leukemia	40	CD123 CAR-T	NA	Recruiting
CD123	NCT04265963	Acute myeloid leukemia	I/II	Refractory or relapsed acute myeloid leukemia	45	CD123 CAR-T	NA	Recruiting
CD123	NCT03672851	Acute myeloid leukemia	I	Refractory or relapsed acute myeloid leukemia	2	Anti-CD123 CAR-T	NA	Terminated
CD123	NCT04014881	Acute myeloid leukemia	I	Refractory/relapsed CD123+ acute myeloid Leukemia	50	Anti-CD123 CAR-T	NA	Recruiting
CD123	NCT02937103	Myeloid malignancies	I/II	Refractory or relapsed myeloid leukemia	45	Anti-CD123 CAR-T	NA	Recruiting
CD123×CLL1	NCT03631576	Acute myeloid leukemia	II/III	Refractory or relapsed acute myeloid leukemia	20	CD123/CLL1 CAR-T	NA	Recruiting
CD133	NCT02541370	Malignancies	I/II	Relapsed/refractory advanced malignancies	20	Anti-CD133-CAR vector-transduced T cells	Median OS was 12 months and the median PFS was 6.8 months. Of 6 progressed after T-cell infusion.	Completed
CD33×CLL1	NCT05248685	Acute myeloid leukemia	I	Refractory or relapsed acute myeloid leukemia	20	Dual CD33/CLL1 CAR T	No results	Recruiting
CD33×CLL2	NCT05467254	Acute myeloid leukemia	I	Refractory or relapsed acute myeloid leukemia	20	CLL1 + CD33 CAR-T	NA	Recruiting
CD38×CLL1	NCT06110208	Acute myeloid leukemia	I	Refractory or relapsed acute myeloid leukemia	18	CLL1 and CD38 dual-target CAR-T	NA	Recruiting
CD44v6	NCT04097301	Acute myeloid leukemia and multiple myeloma	I/II	Relapse or refractory acute myeloid leukemia and multiple myeloma	8	MLM-CAR44.1 T-cells	NA	Terminated
CLL1	NCT05252572	Hematological malignancies	I	Hematological malignancies	36	CLL1 CAR T-cells	NA	Recruiting
CLL1	NCT05467202	Acute myeloid leukemia	I	Refractory or relapsed acute myeloid leukemia	20	CLL1 CAR-T	NA	Not yet recruiting
CLL1	NCT04923919	Acute myeloid leukemia	I	Refractory or relapsed acute myeloid leukemia	100	Anti-CLL1 CART	NA	Recruiting
CLL1	NCT06128044	Acute myeloid leukemia	I	Refractory or relapsed acute myeloid leukemia	70	CB-012	NA	Recruiting
CLL1	NCT04219163	Acute myeloid leukemia	I	Refractory or relapsed acute myeloid leukemia	18	CLL-1 CAR T	NA	Recruiting
EpCAM	NCT02725125	Stomach cancer	II	Advanced stomach cancer	19	EPCAM-targeted CAR-T	NA	Recruiting
EpCAM×CD3	NCT00822809	Epithelial cancers	III	Epithelial cancers with malignant ascites	230	catumaxomab plus prednisolone	Median OS was longer in the catumaxomab plus prednisolone arm than in the catumaxomab arm (124 vs. 86 days)	Completed
EpCAM×CD3	NCT00189345	Ovarian cancer	II	Platinum refractory epithelial ovarian cancer	44	Anti-EpCAM x anti-CD3 (removab)	No responders in the low-dose versus one patient (5%) in the high-dose group with a PR. In the low-dose group, two patients (9%) had stable disease compared with five patients (23%) in the high-dose group. Catumaxomab was well tolerated	Completed
Immunogenic proteins	NCT02157051	Breast cancer	I	HER2-negative stage III-IV breast cancer	42	CD105/Yb-1/SOX2/CDH3/MDM2-polyepitope Plasmid DNA Vaccine	NA	Active, not recruiting
NA	NCT01334047	Ovarian cancer	I/II	Recurrent platinum sensitive ovarian cancer	5	DC-006 vaccine	NA	Terminated
Prostate stem cell antigen	NCT06193486	Prostate cancer	I	Metastatic castration resistant prostate cancer	30	MSGV1-PSCA-8T28Z plus chemotherapy	NA	Recruiting
Prostate stem cell antigen	NCT05805371	Prostate cancer	I	PSCA^+^ metastatic castration-resistant prostate cancer	21	Autologous Anti-PSCA-CAR-4-1BB/TCRzeta-CD19t-expressing T-lymphocytes; PSCA^+^ CAR-T plus radiotherapy	NA	Recruiting
Specific antigen	NCT00846456	Glioblastoma	I/II	Accessible tumor tissue for vaccine production; Glioma grade IV, and a candidate for combined radiation therapy and chemotherapy	20	Dendritic cell vaccine with mRNA from CSCs	NA	Completed
Specific antigen	NCT04888611	Glioblastoma	II	Recurrent brain glioma (WHO grade 4); Relapse with tumor progression	40	GSC-DCV; Camrelizumab	NA	Recruiting
Specific antigen	NCT05341947	Glioblastoma	I	Recurrent glioblastoma; Complete resection of tumor	10	Activated T cells	NA	Not yet recruiting
Specific antigen	NCT03548571	Glioblastoma	II/III	Glioblastoma IDH wild-type, with unmethylated MGMT-gene promotor	60	Drug: Dendritic cell immunization; adjuvant temozolomide	NA	Active, not recruiting
Specific antigen	NCT02074046	Pancreatic cancer	I/II	Pancreatic cancer	40	CSCs vaccine	NA	Completed
Specific antigen	NCT02115958	Nasopharyngeal carcinoma	I/II	Nasopharyngeal carcinoma	40	CSCs vaccine	NA	Completed
Specific antigen	NCT02063893	Breast cancer	I/II	Breast cancer	40	CSCs vaccine	NA	Completed
Specific antigen	NCT02089919	Hepatocellular carcinoma	I/II	Hepatocellular carcinoma	40	CSCs vaccine	NA	Completed
Specific antigen	NCT02084823	Lung cancer	I/II	Lung cancer	40	CSCs vaccine	NA	Completed
Specific antigen	NCT02176746	Colorectal cancer	I/II	Colorectal cancer	40	CSCs vaccine	NA	Completed
Specific antigen	NCT02178670	Ovarian cancer	I/II	Ovarian cancer	40	CSCs vaccine	NA	Completed

## Cancer stem cells and sensitivity/resistance to radiotherapy

### CSCs and radiotherapy sensitivity/resistance

**Fig. 7:**
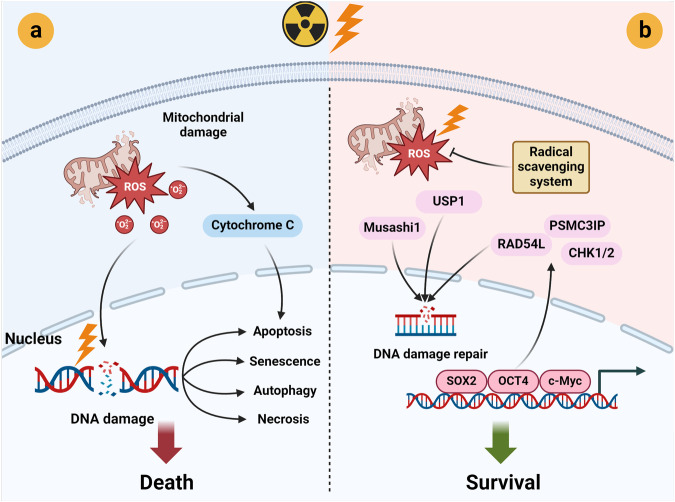
Radioresistance induced by CSCs. **a** As for radiosensitive cancer cells, radiation can induce the production of ROS, which subsequently leads to the accumulation of cytochrome C and apoptosis, and DNA damage that causes various types of cell death. **b** CSCs can be radioresistant due to their high expression of DNA damage repair-associated molecules and powerful radical scavenging system

Radiotherapy, or radiation therapy, is one of the most common and important therapeutic strategies in terms of solid tumor treatment. Radiotherapy exerts its cell-killing effects mainly by inducing DNA damage that is beyond repair, which consequently leads to cell cycle arrest, apoptosis, autophagy, or senescence of target cells.^294^ In this process, ROS is considered a critical mediator (Fig. 7).^814^

Research indicates that radiation can induce the formation of CSCs and that CSCs are less radiosensitive than other cancer cells.^294,815–822^ For instance, radiation-induced radioresistant NSCLC cell line has increased expressions of CSC markers, including SOX2, CD133, and ALDH compared to radiosensitive cells, and upregulating SOX2, a DNA repair regulator, results in more robust radioresistance of these cells.^823^ FOXM1 can also induce SOX2 expression in glioblastoma and induce radioresistance.^824^ In response to radiation, CD133^+^ GSCs exhibit radioresistance by preferentially activating DNA damage checkpoints and repairing DNA damage more effectively.^825^ CD24^−/low^/CD44^+^ breast CSC-enriched mammospheres are also more radioresistant than monolayers breast cancer cells,^826^ and the CD24^−/low^/CD44^+^ breast CSCs contain less ROS levels compared to non-tumorigenic cells.^641^ Likewise, CD133^+^ hepatocellular CSCs are more resistant to radiation than CD133^−^ cells, and suppression of CD133 sensitizes these cells to radiation by breaking cell-cycle arrest and inducing apoptosis.^827^ On the contrary, knocking down CSC markers can sensitize the cells to radiation.^828–830^ This suggests that the acquisition of stem-like properties and radioresistance can be two sides of the same coin. Indeed, studies show that radioresistance is companied by enlarged CSC population in glioma/glioblastoma,^824,831–836^ breast cancer,^828,837–841^ colorectal cancer,^842–845^ lung cancer,^846^ salivary adenoid cystic carcinoma,^847^ oral squamous carcinoma,^848^ head and neck squamous cell carcinoma,^849^ neuroblastoma,^850^ cervical cancer,^841,851^ esophageal squamous cell carcinoma,^852^ ovarian cancer,^853^ and gastric cancer.^854^

The induction mechanism of CSC properties by radiation has not been fully revealed, and some researchers believe CSC properties are acquired through radiation-inducible EMT.^294^ Also, some studies provide insights into the relationship between radiation and CSC formation. Following DNA damage, senescence-associated secretory phenotype (SASP) is released and promotes the emergence of CSCs in MM.^855^ Similarly, High Mobility Group Box 1(HMGB1), a DAMP, is released after radiation and subsequently activates the HIF-1α signaling in pancreatic cancer cells which leads to the acquisition of CSC properties.^856^ Another study regarding glioblastoma shows that the radiation-inducible activation of the K-RAS/ERK/CD44 axis facilitates the stemness of the cells.^857^ And the miR-603 in extracellular vesicles of glioblastoma after radiation targets IGF1 and IGF1R that promote CSC state.^835^ Additionally, following radiation, non-CSCs of breast cancer are converted into CSCs, which can be prevented by Notch inhibition, suggesting a crucial role of the Notch pathway in this transition.^858^

The radioresistance of CSCs depends on their enhanced abilities to repair DNA damage and maintain ROS levels (Fig. 7). MYCN-amplified neuroblastoma cells exhibit increased c-Myc expression, dysregulated DNA repair pathway, stable ROS level after radiation, and CSC properties.^850^ c-Myc plays an important role in radioresistance of nasopharyngeal carcinoma CSCs by upregulating DNA damage checkpoint Checkpoint Kinase 1 (CHK1) and CHK2.^859^ OCT4, a CSC marker, endows radioresistance to head and neck squamous cell carcinoma cells by regulating the homologous recombination factors PSMC3IP and RAD54L, and either upregulation or downregulation of OCT4 diminishes radioresistance of the cells.^860^ THOC2 and THOC5 play an important role in the radioresistance of triple-negative breast cancer cells by upregulating SOX2.^837^ SOX2 can lead to radioresistance by inducing cell cycle arrest to avoid DNA damage checkpoints.^861^ Ubiquitination-Specific Protease 1 (USP1), which is upregulated in GSCs, stabilizes DNA damage response regulators and induces radioresistance of these cells.^862^ Musashi1, a CSC marker, regulates the expression of a DNA-protein kinase catalytic subunit to induce enhanced DNA repair response, which finally endows radioresistance to GSCs.^863^

Several signaling pathways are involved in the acquisition of both stemness and radioresistance. The activation of the JAK2/STAT3 pathway promotes colorectal cancer stemness characterized by increased expression of cyclin D2, which also maintains low levels of DNA damage accumulation.^842^ The TGF-β pathway activation or the WNT/β-Catenin pathway also enhances not only stemness but also radioresistance of breast cancer, salivary adenoid cystic carcinoma, colon cancer, cervical cancer, or gastric cancer.^839,844,847,851,854,864^ MiR-19b can downregulate FBXW7 expression and consequently activate the WNT/β-Catenin pathway, which eventually leads to stemness enhancement and radioresistance.^843^ SFRP2 is downregulated in glioma patients treated with radiotherapy, and a study shows that SFRP2 diminishes stemness and radioresistance of glioma cells by inhibiting the WNT/β-Catenin signaling.^833^ Besides, the Forkhead Box Q1 (FOXQ1)/Sirtuin 1 (SIRT1)/β-Catenin axis and the Ecotropic Virus Integration Site 1 (EVI1)/β-Catenin can also mediate stemness and radioresistance of colorectal cancer.^845,865^ The activation of the PI3K/AKT/mTOR pathway decreases apoptosis thus inducing radioresistance of prostate CSCs.^866^ Also, Tribble 2 activates the mTOR pathway and induces stemness and radioresistance in esophageal squamous cell carcinoma.^852^ In glioblastoma, the cyclin-like protein Spy1 endows the cancer cells with self-renewal abilities and downregulates CAP-Gly Domain-Containing Linker Protein 3 (CLIP3) whose expression leads to the glycolytic flux that induces radioresistance.^832,867^ The Proliferating Cell Nuclear Antigen (PCNA)-Associated Factor (PAF) supports GSC maintenance and promotes radioresistance by inducing translesion DNA synthesis.^868^ Activation of NRP1 not only improves stemness but also potentiates radioresistance of breast cancer cells by reducing radiation-mediated apoptosis.^840^ Integrin β1 increases stemness of oral squamous carcinoma cells and induces radioresistance by suppressing radiation-induced apoptosis.^869^

### Preclinical studies on improving radiosensitivity by targeting CSCs

Efforts have been made to restore radiosensitivity by inhibiting CSCs in preclinical studies. DNA-Dependent Protein Kinase (DNA-PK) stabilizes SOX2 and maintains the stemness of GSCs, and NU7441, a DNA-PK inhibitor, can effectively reduce the stem cell sphere formation and sensitize the tumor to radiotherapy in vivo.^831^ Combining radiotherapy with glimepiride, an agent to treat type 2 diabetes, can disturb GSC maintenance and sensitize the tumor to radiation by reducing glycolysis.^832^ MiR-7-5p can reduce stemness of colorectal CSCs and sensitize these cells to radiation by downregulating the stemness-associated transcription factor, KLF4.^870^ Delivery of miR-145 that targets multiple stemness-related transcriptional factors reduces stemness and reverse radioresistance of colorectal CSCs.^871^ The lncRNA Transmembrane Phosphatase With Tensin Homology Pseudogene 1 (TPTEP1) interacts with miR-106a-5p and thus activates the P38/MAPK pathway that suppresses stemness and radioresistance of glioma cells.^834^ An Oncostatin M Receptor (OSMR) promotes mitochondrial respiration in GSCs, and suppression of this receptor sensitizes the cells to ionizing radiation.^872^ Apigenin can attenuate stemness of glioblastoma by downregulating HIF-1α and NF-κB and sensitizing the cells to radiotherapy due to reduced glycolysis.^873^ MiR-146b-5p can target the Hu antigen R and increase lncR-p21 which leads to inhibition of β-Catenin.^874^ This process attenuates stemness and increases apoptosis and radiosensitivity of the cells.^874^ Silencing Human Telomerase Reverse Transcriptase (hTERT) abolishes telomerase activity, reduces stemness, and reverses the radioresistance of a radioresistant nasopharyngeal carcinoma cell line.^875^ Given that miR-210 induces hypoxia adaption and maintains stemness of GSCs, knockdown of miR-210 abolishes CSC markers and endows radiosensitivity to these cells.^876^ Inhibition of integrin a6 leads to reduced DNA damage response and normalizes cell cycle pathways, which eventually helps overcome radioresistance and diminish stemness of the GSCs.^836^ Methyltransferase-like 14 and miR-99a-5p can downregulate Tribble 2, and the Tribble 2-induced activation of the mTOR pathway can be inhibited by an H-Istone Deacetylase 2 (HDAC2) inhibitor and restore radiosensitivity of the esophageal squamous CSCs.^852^ Restoration of E3 ubiquitin ligase C Terminus Of HSC70-Interacting Protein (CHIP) not only reduces expression of stemness of NSCLC cells but also sensitizes the cells to radiotherapy by improving apoptosis via inhibition of the PBK/ERK axis.^877^ BEZ235, a dual PI3K/mTOR inhibitor, can effectively sensitize prostate CSCs to radiotherapy by reducing the stemness of the cells.^866^

### Clinical trials targeting CSCs combined with radiotherapy

Despite the efforts made to increase radiosensitivity by targeting CSCs in preclinical trials, few clinical trials that combine radiotherapy and CSC-targeting therapies are carried out (Table 6). A study tried to set the periventricular stem cell niche as additional target volumes in newly diagnosed high-grade glioma to eliminate the potential CSC pool. However, all 4 enrolled patients had adverse events and did not complete the study (NCT02039778). Another phase I study (NCT01068327) evaluated the safety and efficacy of nelfinavir, an Akt inhibitor, plus stereotactic body radiotherapy in treating locally advanced borderline or unresectable pancreatic adenocarcinoma. Among the 46 patients enrolled, sixteen patients experienced grade ≥2 adverse events, and grade 3−4 adverse events only occurred in 1 patient. The median overall survival of all the patients was 14.4 months. This trial concludes that concurrent stereotactic body radiation therapy (SBRT) (40 Gy) plus nelfinavir (1250 mg BID) was tolerable and safe for patients with locally advanced pancreatic cancer, but the efficacy of this combination still required investigations.^878^

**Table 6:** Clinical studies on combination of radiotherapy and CSC-targeting therapies

Target	NCT Number	Tumor type	Phases	Conditions	Enrollment	Interventions	Study Results	Study Status
OCT4, SOX2, Nanog, ABCB1, ABCG2	NCT02423811	Esophageal cancer	II	Newly diagnosed stage II and III esophageal squamous cell carcinoma	20	Fursultiamine + Concurrent Chemoradiotherapy	NA	Completed
AKT/ERK	NCT04854044	Glioblastoma	I	Recurrent glioblastoma	0	Presurgery ONC201 + Radiotherapy	NA	Withdrawn
ALDH	NCT01777919	Glioblastoma	II	Newly diagnosed glioblastoma	32	Disulfiram/copper before chemo-radiotherapy	NA	Unknown
Periventricular stem cell niche	NCT02039778	Glioma	NA	Newly diagnosed high grade glioma after surgery	4	Radiotherapy + Temozolomide	All 4 patients had adverse events and did not complete the study	Completed
Notch	NCT01119599	Glioma	I	Newly diagnosed glioma	22	RO4929097 + Radiotherapy + Temozolomide	NA	Completed
AKT	NCT05172245	Head and neck squamous cell carcinoma	I	Locally advanced head and neck squamous cell carcinoma	46	Ipatasertib + Chemo-radiotherapy	NA	Recruiting
Notch	NCT01217411	Brain metastases	I	Newly diagnosed metastatic disease to the brain	5	RO4929097 + Radiotherapy	Study terminated early due to low accrual and discontinuation of investigational study drug.	Terminated
AKT	NCT00694837	Glioblastoma	I	Newly diagnosed glioblastoma	6	Nelfinavir + Radiotherapy + Temozolomide	NA	Completed
AKT	NCT01068327	Pancreatic cancer	I	Locally advanced pancreatic cancer	46	Presurgery Nelfinavir + Radiotherapy	Median OS: 14.4 months	Completed
AKT	NCT03256916	Cervial cancer	III	Untreated locally advanced cervical cancer	348	Nelfinavir + Chemo-radiotherpay	NA	Recruiting
AKT	NCT01485731	Cervical cancer	I	Locally advanced cervical cancer	8	Nelfinavir + Chemo-radiotherpay	NA	Completed
PI3Kα	NCT02537223	Head and neck squamous cell carcinoma	I	Locally advanced head and neck squamous cell carcinoma	9	BYL719 + Chemo-radiotherapy	NA	Completed
PI3K	NCT02128724	Non-small cell lung cancer	I	Any-stage non-small cell lung cancer	21	BKM120 + Radiotherpay	3 of 21 patients had serious adverse events	Completed

## Cancer stem cells and targeted therapy

Targeted therapy for tumors, a pivotal component of precision medicine, entails identifying specific carcinogenic sites at the molecular level and employing drugs to selectively target these areas, thereby achieving therapeutic objectives.^879^ Due to its notable advantages in prolonging patient survival, targeted therapy has garnered increasing attention, with a considerable number of treatments earning Food and Drug Administration (FDA) approval for tumor management.^880^ However, resistance to targeted therapy remains a significant consideration during treatment, emphasizing the critical role of CSCs.^881^

### Thoracic tumors

**Fig. 8:**
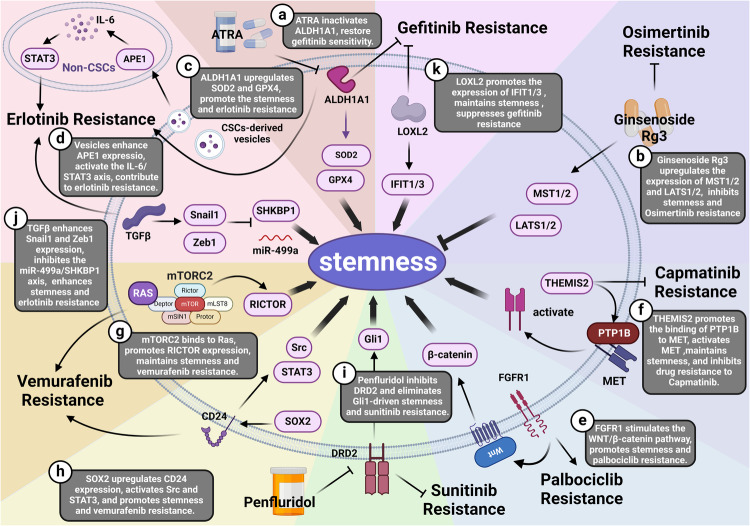
Targeted drug resistance of CSCs (except liver cancer). (**a, k)** gefitinib resistance (**b**) osimertinib resistance (**c**, **d**, **j**) erlotinib resistance (**e**) palbociclib resistance (**f**) capmatinib resistance (**g**, **h**) vemurafenib resistance (**i**) sunitinib resistance

Resistance of thoracic tumors to targeted drugs such as gefitinib, osimertinib, erlotinib, afatinib, palbociclib, and lapatinib can be partially attributed to the presence of a rare subset of CSCs. NSCLC stem cells with elevated expression levels of ALDH1A1 and CD44 demonstrate heightened resistance to gefitinib. Notably, ALDH1A1 activity can be neutralized by ATRA, restoring sensitivity (Fig. 8a).^882^ Osimertinib-resistant lung cancer cells exhibit increased stemness traits. Ginsenoside Rg3 has been identified as a sensitizing factor for osimertinib by activating the Hippo pathway (Fig. 8b).^883^ Furthermore, NSCLC cells resistant to erlotinib and afatinib demonstrate enhanced CSCs-related characteristics.^884,885^ The CSCs’ marker *ALDH1A1* has been identified as a critical gene for erlotinib resistance in lung cancer cells. In ALDH1A1-positive cells, the anti-ROS system is activated, leading to significant upregulation of its associated enzymes Superoxide Dismutase 2 (SOD2) and Glutathione Peroxidase 4 (GPX4) during ALDH1A1-induced erlotinib resistance (Fig. 8c).^886^ Additionally, besides their intrinsic resistance to targeted therapy, CSCs confer drug resistance to non-CSCs by secreting vesicles. Vesicles originating from lung CSCs augment Apurinic Endonuclease 1 (APE1) expression in NSCLC, subsequently activating the IL-6/STAT3 axis, thus contributing to erlotinib resistance (Fig. 8d).^887^ Further, Fibroblast Growth Factor Receptor 1 (FGFR1), which promotes breast cancer stemness through the WNT/β-Catenin pathway, was identified as a key factor in palbociclib resistance, a CDK4/6-related targeted drug (Fig. 8e).^888^ While the majority of studies suggest that heightened stemness in thoracic tumors fosters resistance to targeted drugs, Huang et al. reported a contrasting finding. Specifically, they demonstrated that overexpression of Thymocyte Expressed Molecule Involved In Selection 2 (THEMIS2) in breast cancer promotes the binding of Protein-Tyrosine Phosphatases 1B (PTP1B) to MET, leading to MET activation, and ultimately sustaining stemness characteristics. Interestingly, THEMIS2 expression was positively associated with lapatinib sensitivity and inversely correlated with chemotherapy sensitivity (Fig. 8f).^889^

### Liver cancer

**Fig. 9:**
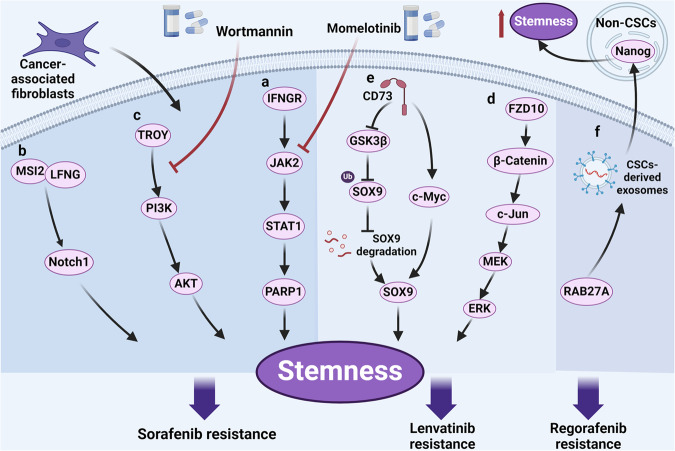
Targeted drug resistance of liver CSCs. **a** IFNGR stimulation of the JAK2/STAT1/PARP1 pathway is responsible for stemness maintenance and sorafenib resistance, and can be reversed by the JAK2 inhibitor momelotinib. **b** MSI2 binds LFNG to stimulate the Notch1 pathway to upregulate tumor cell’ stemness and sorafenib resistance. **c** Wortmannin inactivates the TROY/PI3K/AKT axis triggered by CAFs to inhibit the stemness of tumor cells and restore their sensitivity to sorafenib. **d** FZD10 contributes to stemness maintenance and lenvatinib resistance by activating the β-Catenin/c-Jun/MEK/ERK axis. **e** CD73 upregulates the c-Myc/SOX9 axis and inhibits GSK3β to hinder the ubiquitination and degradation of SOX9, ultimately maintaining the stemness characteristics and lenvatinib resistance of tumor cells. **f** CSCs release exosomes to upregulate Nanog expression in a RAB27A-dependent manner, promoting stemness characteristics and regorafenib resistance of tumor cells

CSCs play a pivotal role in the resistance of liver cancer to various targeted drugs, including sorafenib, trametinib, lenvatinib, and regorafenib. Chang et al. uncovered a negative correlation between the expression of YAP1, a promoter of stemness-related genes *SOX2* and *OCT4*, and the sensitivity of liver cancer cells to sorafenib.^890^ Viral infection-associated hepatocellular carcinoma cells (vHCC) exhibit resistance to sorafenib.^891^ The activated Interferon-Gamma Receptor (IFNGR)/JAK2/STAT1/Poly(ADP-Ribose) Polymerase 1 (PARP1) pathway in vHCC maintains stemness, leading to resistance to sorafenib. Conversely, the JAK2 inhibitor momelotinib reverses vHCC drug resistance (Fig. 9a).^892^ Integration of the hepatitis B virus gene *HBx-ΔC* contributes to liver cancer stemness and resistance to sorafenib and 5-fluorouracil.^893^

Activation of Notch and PI3K/AKT pathways in CSCs is pivotal in developing resistance to targeted drugs. CD44v6 serves as a marker of liver CSCs positively associated with sorafenib resistance. Musashi2 (MSI2) overexpression in CD44v6-positive liver CSCs contributes to sorafenib resistance by binding Lunatic Fringe (LFNG) to activate the Notch1 pathway (Fig. 9b).^894^ Highly expressed TROY in liver cancer correlates with stemness characteristics and sorafenib resistance, while wortmannin inactivates the TROY-induced PI3K/AKT pathway, restoring sensitivity to sorafenib (Fig. 9c).^505^ Plasma-activated medium (PAM) enhances the efficacy of trametinib and sorafenib in CSC-rich liver cancer cell populations by inducing various forms of cell death.^895^ FZD10 expression significantly increases in lenvatinib-resistant liver cancer cells, maintaining liver CSC characteristics by activating the WNT/β-Catenin pathway and β-Catenin/c-Jun/MEK/ERK axis, thereby contributing to lenvatinib resistance (Fig. 9d).^527^ CD73, a marker of liver CSCs, upregulates the c-Myc/SOX9 axis, inhibiting GSK3β and the ubiquitination and degradation of SOX9, thereby conferring stemness characteristics to liver cancer (Fig. 9e).^896^

Cytokines and exosomes are critical factors in conferring resistance to targeted drugs in liver cancer cells. Kahraman et al. demonstrated that the application of targeted drugs, such as sorafenib and regorafenib, enriches liver CSCs, indirectly suggesting resistance to targeted therapy. Further mechanistic studies showed that IL-8 derived from the liver cancer niche maintains the stemness phenotype and inhibits sensitivity to sorafenib.^897^ CSCs can also confer resistance to targeted drugs to differentiated malignant cells. Exosomes released by hepatic CSCs in Ras-Related Protein Rab-27A (RAB27A)-dependent manner confer regorafenib resistance to differentiated hepatoma cells by inducing the upregulation of Nanog expression (Fig. 9f).^898^

### Other tumors

The relationship between resistance to targeted therapy and CSCs is confirmed in various tumors, including melanoma, colorectal cancer, renal cell carcinoma, osteosarcoma, and oral squamous cell carcinoma. Vemurafenib-resistant melanoma cells exhibit higher expression of CSCs-related markers such as CD271 and fibronectin.^899^ The Mechanistic Target Of Rapamycin Complex 2 (mTORC2) confers stemness characteristics to melanoma-initiating cells in Rapamycin-Insensitive Companion Of MTOR (RICTOR)-dependent manner, promoting melanoma cell resistance to vemurafenib (Fig. 8G).^900^ Overexpression of the stemness-related gene *SOX2* in melanoma correlates closely with vemurafenib resistance. *SOX2* binds to the promoter of *CD24* to upregulate its expression, activating Src and STAT3 and conferring adaptive resistance rather than acquired resistance to melanoma cells against targeted therapy (Fig. 8h).^901^ Additionally, the NRG-1β/ErbB-3 axis and the AKT pathway are critical for colon CSC resistance to vemurafenib.^902^

The antipsychotic drug penfluridol inhibits Dopamine Receptor D2 (DRD2) to eliminate the CSCs associated phenotype of renal cell carcinoma mediated by the hedgehog pathway, inducing apoptosis and autophagy, and enhancing the efficacy of the targeted drug sunitinib (Fig. 8i).^903^ However, the use of sunitinib also enriches CSC subsets in renal cell carcinoma. Sunitinib enhances Estrogen Receptor β (ERβ) expression by upregulating lncRNA-ECVSR, activating HIF-2α, and promoting the emergence of a CSC phenotype.^904^ MiR-499a suppresses resistance to the EGFR inhibitor erlotinib in CD166^+^ osteosarcoma stem cells. TGFβ-induced enhancement of Snail1 and Zeb1 expression suppresses the miR-499a/SHKBP1 axis, enhancing stemness characteristics and erlotinib resistance (Fig. 8j).^905^ Conversely, highly expressed Lysyl Oxidase-Like 2 (LOXL2) in oral squamous cell carcinoma correlates positively with the activation of the EMT program and the maintenance of stemness. LOXL2 promotes the expression of stemness-related genes and EGFR in an IFIT1- and IFIT3-dependent manner, ultimately rendering oral squamous cell carcinoma more sensitive to the EGFR inhibitor gefitinib (Fig. 8k).^906^

There is a scarcity of ongoing or completed clinical trials specifically targeting CSCs and their resistance to targeted therapies. Clinical trial NCT01215487 aims to investigate whether the content of CML stem cells can serve as a predictor of efficacy in CML patients undergoing imatinib therapy. Another trial, NCT03481868, is centered on epigenetics and resistance to tyrosine kinase inhibitors in CML stem cells. However, no results from these trials have been reported.

## Therapeutic strategies targeting CSCs

### Targeting classic markers of CSCs

Markers of CSCs, whether they are cell surface markers like CD13, CD44, and CD133, or intracellular markers such as Nanog, ALDH1, and SOX2, are effective molecules for identifying the rare population of CSCs and represent important targets for eliminating their various malignant biological behaviors. For instance, CD13 expression in liver CSCs positively correlates with the activation of the TGF-β-mediated EMT program, which enhances stemness characteristics while inhibiting ROS accumulation. Inhibiting CD13 induces apoptosis of liver CSCs.^907^ Liposomes modified with CD44 monoclonal antibodies exhibit enhanced anti-tumor efficacy by effectively targeting CSCs.^908^ Similarly, the plant extract emodin serves as a specific inhibitor of the liver CSCs marker CD44, exerting anti-tumor effects.^909^ Targeting CD133^+^ CSCs in gastric cancer with anti-CD133 CAR-T cells significantly inhibits CSCs-mediated tumor progression and treatment resistance.^910^ Targeting intracellular stemness-related marker Nanog effectively reduces the stemness of breast CSCs.^911^ Inhibiting the expression of intracellular stemness-related marker ALDH1 using the cell cycle regulatory kinase wee1 inhibitor MK1775 eliminates the stemness characteristics of MM.^912^ Moreover, FDA-approved drugs like ATRA and Suberoylanilide Hydroxamic acid (SAHA) can specifically target CSCs based on the expression of cell surface marker CD133 and intracellular marker Nanog. Their combination relieves the inhibition of Tet Methylcytosine Dioxygenase 2 (TET2) and PTEN by inactivating the lncRNA MIR22HG/miR‐22 axis, ultimately attenuating the stemness characteristics of liver cancer and inducing apoptosis of liver CSCs.^913^ Similarly, ATRA effectively inhibits the expression of cell surface marker CD44 and stemness-related genes *ALDH*, *SOX2*, and *KLF4* to target gastric CSCs and hinder gastric cancer progression.^914^

Cell surface markers, such as CD123, and intracellular markers, such as Nanog, represent commonly utilized targets in these clinical trials, predominantly through CAR-T cells, specific antibodies, and targeted drugs (NCT04272125, NCT02232646). However, the majority of these trials are in phase I or phase II, with few reporting outcomes. Limited survival data from completed trials make it challenging to draw definitive conclusions regarding the clinical efficacy of targeting CSC markers in relapsed or refractory tumors.

### Targeting the classic pathway of CSCs

**Fig. 10:**
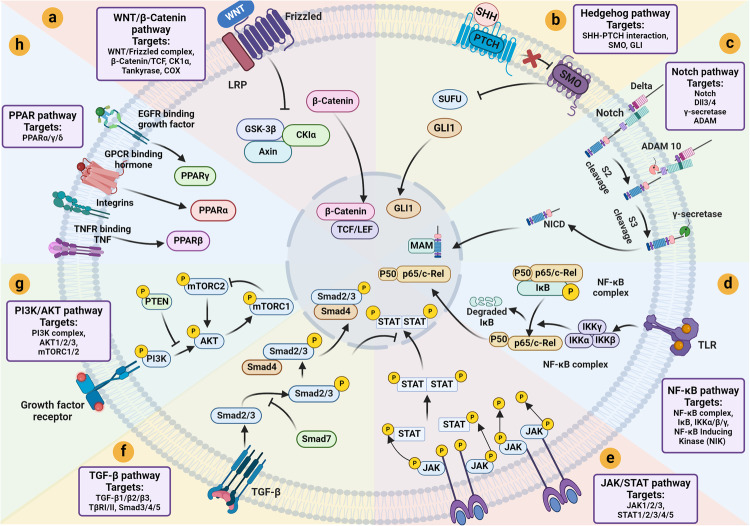
Targeting CSCs through classical signaling pathways. **a** WNT/β-Catenin pathway. Commonly developed targets include WNT/Frizzled complex, β-Catenin/TCF, CK1α, tankyrase, and COX. **b** Hedgehog pathway. Commonly developed targets include SHH-PTCH interaction, SMO, and GLI. **c** Notch pathway. Commonly developed targets include Notch, Dll3/4, γ-secretase, ADAM. **d** NF-κB pathway. Commonly developed targets include NF-κB complex, IκB, IKKα/β/γ, NF-κB inducing kinase (NIK). **e** JAK/STAT pathway. Commonly developed targets include JAK1/2/3, STAT1/2/3/4/5. **f** TGF-β pathway. Commonly developed targets include TGF-β1/β2/β3, TβRI/II, Smad3/4/5. **g** PI3K/AKT pathway. Commonly developed targets include PI3K complex, AKT1/2/3, mTORC1/2. **h** PPAR pathway. Common targets that have been developed include PPARα/γ/δ

The malignant biological behavior of CSCs is underpinned by multiple interacting signaling pathways, hinting at the potential significance of targeting classic pathways in CSCs (Fig. 10). Preclinical investigations have validated the feasibility of targeting signaling pathways within CSCs. For instance, ICG-001, a WNT pathway inhibitor, effectively eliminates the stemness and metastasis phenotypes of colorectal cancer cells by suppressing the downstream gene of the WNT pathway, *Myeloid Ecotropic Viral Insertion Site 1 (MEIS1)*.^915^ Similarly, a complex comprising [PdCl(terpy)](sac)2H2O and niclosamide, designed by Karakas et al., enhances the therapeutic efficacy against breast cancer by inhibiting the WNT pathway and inducing apoptosis of CSCs.^916^ The sonic hedgehog pathway, when activated in pancreatic CSCs, can be attenuated by sulforaphane (SFN), derived from cruciferous vegetables, which reduces GLI activity, suppresses stemness, and induces apoptosis.^917^ Additionally, silencing the Notch2 pathway significantly inhibits the stemness and metastatic phenotypes of bladder cancer cells, revealing a promising target to impede bladder cancer progression.^918^ The inhibitory effects of nonsteroidal anti-inflammatory drugs on colorectal CSCs could be attributed to the inactivation of the Notch pathway and the activation of the PPARγ pathway.^919^ The JAK2-specific inhibitor CYT387 markedly suppresses the paclitaxel-induced enhancement of stemness characteristics in ovarian cancer by attenuating the activity of the JAK2/STAT3 pathway.^920^ Natural products such as curcumin from turmeric and epigallocatechin-3-gallate (EGCG) from green tea have demonstrated inhibition of breast CSCs activity by deactivating the JAK/STAT and NF-κB pathways.^921^ Moreover, celastrus orbiculatus extract had demonstrated the capability to deactivate the TGF-β/Smad pathway by inhibiting Smad3/4, which ultimately results in the suppression of gastric CSCs.^922^ Hongwiangchan et al. synthesized hydroquinone 5-O-cinnamoyl ester of renieramycin M (CIN-RM), which exhibits inhibitory effects on lung CSCs by deactivating the AKT/PI3K pathway and downstream c-Myc.^923^ Furthermore, GSK-458 effectively disrupts the stemness characteristics of CSCs and induces caspase-3-mediated cell death by inactivating the PI3K/mTOR pathway.^924^

While preclinical data indicate the feasibility of targeting signaling pathways within CSCs to eliminate them, there are currently limited corresponding clinical trials. Most ongoing clinical trials solely utilize signaling pathway inhibitors in patients. However, discerning whether the anti-tumor effect necessitates targeting CSCs remains challenging (NCT00106145, NCT01608867, NCT00844064). More sophisticated clinical trial designs are imperative to ascertain the effectiveness of targeting signaling pathways within CSCs.

### Targeting the niche of CSCs

The supportive niche surrounding CSCs represents another crucial protective factor in maintaining their stemness characteristics.^4^ In diffuse large B-cell lymphoma, a significant positive correlation was observed between the stemness score and the scores of immune cells and stromal cells, highlighting the importance of targeting the niche as a key strategy for eliminating CSCs.^925^ Components of the CSCs niche, including the hypoxic microenvironment, acidic microenvironment, TAMs, CAFs, and cytokines, have been shown to closely influence the stemness maintenance and survival of CSCs.

Hypoxia-induced HIF-2α serves as a key factor in maintaining stemness in breast cancer. Mechanistically, inhibition of HIF-2α effectively attenuates the stemness phenotypes through inactivating the PI3K/AKT/CD44 pathway.^926^ Acidosis, a hallmark of the tumor microenvironment, acts as a promoting factor for the stemness phenotype in melanoma, prostate cancer, colorectal cancer, and gastric cancer.^927^ The extracellular acidic microenvironment may become another promising target for the treatment of CSCs. Doherty et al. proposed significant inactivation of the IFN pathway in breast CSCs, suggesting that IFN-β could serve as a new targeted therapy for breast CSCs. Exogenous IFN-β induces breast CSCs to transition toward a non-stemness phenotype and promotes lymphocyte infiltration.^928^ Further mechanistic studies have indicated that overexpression of non-phosphorylated IFN-Stimulated Gene Factor 3 (ISGF3) in breast CSCs is responsible for their stemness phenotype and invasive behavior. Exogenous IFN-β therapy significantly phosphorylates ISGF3 to suppress the stemness characteristics of breast CSCs.^929^ Chemokines are implicated in tumor progression as components of the tumor microenvironment. C-X-C Motif Chemokine Receptor 2 (CXCR2), upregulated by Galectins-3 (Gal-3), has been identified as a renal cell cancer stemness maintenance factor.^930^ IL-8 transactivates the EGFR/HER2 pathway through CXCR1 and CXCR2 activation in SRC-dependent manner, ultimately enhancing the stemness characteristics of breast CSCs.^931^ This further suggests that chemokines can serve as potential targets for controlling CSCs.

Highly expressed CD51 in TAMs maintains the M2 polarized phenotype and promotes TGF-β1 secretion. Niche-derived TGF-β1 further activates the TGF-β/Smad pathway to sustain the stemness characteristics of pancreatic cancer. CD51-based TAM-targeted therapy may become another option to control pancreatic CSCs.^255^ TAMs-derived CCL2 effectively activates the AKT pathway in breast cancer cells, facilitating the nuclear transfer of β-Catenin and ultimately sustaining the stemness and EMT-related phenotypes of breast cancer.^274^ Similarly, TAMs-derived CCL22 was identified as a factor promoting stemness and invasiveness in esophageal squamous cell carcinoma. CCL22 in the tumor microenvironment activates the FAK/AKT axis to bind and phosphorylate GLI1, thereby activating the hedgehog pathway.^932^ Additionally, M1-TAMs secrete IL-6 to upregulate the STAT3/Thrombospondin-1 (THBS1) axis, maintaining the stemness of oral squamous cell carcinoma.^933^ The interaction of TAMs and CAFs with non-CSCs populations can promote the transformation of CD44^+^CD24^+^ non-CSCs into CD44^+^CD24^−^ breast CSCs. Mechanistically, Rab13 supports the stimulation of IL-8 derived from the breast CSC niche to promote membrane translocation of CXCR1/2, ultimately upregulating the stemness of breast cancer. Upon inhibition of Rab13 with bardoxolone-methyl, a notable suppression of breast CSCs was observed.^934^ IL-6 and IL-33 secreted by CAFs significantly enhance the activity of 5-LO in MDSCs, stimulating downstream Leukotriene B4 (LTB4)/Leukotriene B4 Receptor Type 2 (BLT2) axis to promote stemness and chemoresistance of intrahepatic cholangiocarcinoma.^287^ Furthermore, IL-6 and IL-8 derived from myofibroblasts in the tumor microenvironment activate the Notch/Hairy And Enhancer Of Split 1 (HES1) and STAT3 pathways, enhancing the CSCs population in early colorectal cancer.^935^ This further suggests that CAFs in the niche are another promising target for controlling CSCs. Unlike most evidence supporting CAFs as protectors of CSCs, McAndrews et al. reported that the presence of αSMA^+^ CAFs was associated with suppressed activity of LGR5^+^ colorectal CSCs, increased regulatory T cells and decreased CD8^+^ T cells.^936^

While preclinical studies indicate the feasibility of targeting the niche to affect CSCs, there remains a notable absence of relevant clinical trials. Current ongoing clinical trials involve interventions such as the use of autologous activated T cells and CSC vaccines comprising dendritic cells, T cells, B cells, and CSC-derived antigens to reprogram the CSC niche (NCT05341947, NCT02074046, NCT00846456). However, the majority of these trials are in phases I and II, with no conclusive experimental outcomes reported yet.

### Targeting CSC through other approaches

In addition to targeting the markers, signaling pathways, and niches of CSCs, other potential approaches to eliminate CSCs include modulating stemness-related genes, abnormal metabolism, non-coding RNA, etc. The Protein Arginine Methyltransferase Family (PRMTs) has emerged as a key player in tumor progression.^937,938^ Feng et al. highlighted PRMTs as crucial enzymes regulating ovarian cancer stemness, suggesting that PRMT inhibitors could serve as potential targeted therapeutics for ovarian CSCs.^939^ Dysregulation of iron metabolism, lipid metabolism, and mitochondrial function contributes to stemness maintenance. Katsura’s team demonstrated a close association between imbalanced iron metabolism and tumor stemness. Deferasirox application effectively downregulates stemness in esophageal cancer and oral cancers.^940^ High-fat diets activate lipid metabolism via PPARα and PPARδ, enhancing intestinal stem cell function and tumorigenesis.^941^ Activation of the FOXM1/PRDX3 axis in mitochondria is essential for endometrial CSCs’ survival, suggesting mitochondria as a feasible CSC target.^942^ Similarly, the mitochondria function-associated FOXM1/PRDX3 pathway is indispensable for colorectal CSCs survival, with its induced upregulation of CD133 expression significantly contributing to colorectal stemness.^943^

Non-coding RNAs play a crucial role in the intricate regulatory network governing tumor progression and the stemness maintenance of CSCs.^944,945^ Utilizing a delivery vector termed human telomerase reverse transcriptase promoter-driven VISA (TV), circular RNA RANBP2-Like And GRIP Domain-Containing Protein 6 (circRGPD6) is transported to breast CSCs to impede their tumor initiation and metastasis potential. Mechanistically, TV-circRGPD6 acts as a sponge for miR-26b, alleviating its suppression of yes-associated factor 2.^946^ The DGCR8/circKPNB1/SPI1 positive feedback loop, persistently activated in glioblastoma, sustains the upregulation of circKPNB1, which subsequently activates the SPI1/TNF-α/NF-κB axis, maintaining stemness of glioblastoma.^455^ Elevated circ_0007385 in NSCLC functions as a stemness-promoting factor by sponging miR-493-3p to alleviate its inhibition of ras-related protein Rab-22A.^947^ Similarly, lung CSCs-secreted lncRNA Mir100hg is delivered via exosomes to non-CSCs, targeting miR-15a-5p and miR-31-5p, thereby promoting lung cancer progression.^948^ MiR-148a, inversely correlated with the expression of stemness-related genes *SOX2*, *OCT4*, and *Nanog*, attenuates the stemness of esophageal squamous cell carcinoma by inhibiting Activin A Receptor, Type I (ACVR1).^949^ Non-coding RNA emerges as a promising therapeutic avenue for targeting CSCs.

While preclinical studies have shown promise in targeting CSCs through alternative pathways, their clinical efficacy remains to be established. We provide a summary of pertinent clinical trials in Table 7. Interventions in these trials encompass CSC vaccines, repurposing of existing drugs (such as metformin), and targeting of genes potentially linked to stemness (NCT02084823, NCT01440127, NCT03298763). However, most investigations are in early phases (phase I and phase II), with limited comparison between treatment strategies targeting CSCs and standard therapies. The available results from a few clinical trials do not conclusively demonstrate significant patient benefit from CSC-targeted treatments (NCT01579812, NCT02001974, NCT02001974).

**Table 7:** Clinical trials targeting CSCs through other approaches

NCT Number	Tumor type	Phases	Conditions	Enrollment	Interventions	Study Results	Study Status
NCT01440127	Colorectal cancer	I	Colorectal cancer; Intent to undergo disease resection or biopsy	9	Drug: Metformin	NA	Terminated
NCT01579812	Gynecologic cancer	II	Diagnosis of ovarian, fallopian, or primary peritoneal cancer	90	Drug: Metformin	The percentage of 38 patients who completed treatment and were alive without recurrence at 18 months: 58.1%; OS: 43 months	Completed
NCT00852566	Chronic myeloid leukemia	II	Patients must have CML	46	Drug: Imatinib; Dasatinib	NA	Completed
NCT01397734	Chronic myelogenous leukemia	I	Diagnosis of chronic myelogenous leukemia	7	Drug: Arsenic trioxide	NA	Terminated
NCT02353728	Chronic myelogenous leukemia	II	Documented diagnosis of Ph^+^ Chronic phase CML	16	Drug: Nilotinib	Percentage of Leukemic Stem Cells Present in Bone Marrow Aspirate Samples: 37.3% (3 months), 2.32% (1 months), 0.7% (12 months), 7.04% (1 months), 24.2% (12 months), 9.57% (1 months), 14.6% (3 months), 5.37% (12 months), 10.9% (1 months), 4.33% (3 months), 10.9% (1 months), 13.7% (3 months), 11.7% (3 months), 19.3% (12 months), 3.67% (3 months)	Completed
NCT02001974	Breast cancer	I	Diagnosis of breast cancer with metastatic disease with HER-2 negative status and eligible for treatment with paclitaxel	33	Drug: Reparixin; Paclitaxel	The ratio of the 6-month progression-free survival rate (%) of the Paclitaxel combined with reparixin oral 400 mg group and the Paclitaxel combined with reparixin oral 50% increase to 1200 mg group was 1/4 vs. 4/23	Completed
NCT02642094	Breast cancer	II	Diagnosed with DCIS/LCIS, atypical lobular hyperplasia (ALH) or ADH lesions detected by pathology; Women scheduled for mastectomy or lumpectomy	58	Drug: Rapamycin	Ratio of percent nuclei with positive staining for Ki67 before and after treatment: 8.235 vs. 3.666; The ratio of sphere formation efficiency of mammary stem cells in the control group and the treatment group: 3.681 vs. 0.717	Terminated
NCT01861054	Breast cancer	II	No prior treatment by surgery, radiotherapy, hormone therapy; Be willing to undergo two mandatory tumor biopsies	20	Drug: Reparixin	CSC markers ALDH^+^ and CD24^−^/CD44^+^measured by flow cytometry decreased by ≥ 20% in 4/17 and 9/17 evaluable patients, respectively	Terminated
NCT01190345	Breast cancer	II	Primary breast cancer treated in the neoadjuvant setting	75	Drug: bevacizumab	NA	Completed
NCT05701215	Chronic myelogenous leukemia	II	Patients with diagnosis of chronic phase CML with cytogenetic the philadelphia (Ph) chromosome; At least 3 years of TKI therapy	10	Drug: Venetoclax	NA	Recruiting
NCT02859415	Solid tumors	I/II	Lung cancer, esophageal carcinomas, thymic neoplasms, germ cell tumors, malignant pleural mesotheliomas or chest wall sarcomas, gastric, colorectal, pancreas or renal cancers, and sarcomas metastatic to thorax	3	Drug: Mithramycin	No survival data or CSC-related data reported	Terminated
NCT03298763	Lung cancer	I/II	Inoperable stage IIIb/IV lung adenocarcinoma; EGFR mutation and EML4-ALK translocation negative	46	Genetic: MSCTRAIL; Drug: Placebo	NA	Recruiting
NCT01119599	Glioma	I	Newly diagnosed malignant gliomas with the exception of pure anaplastic oligodendroglioma	22	Drug: RO4929097; temozolomide; 3-dimensional conformal/intensity-modulated radiation therapy	NA	Completed
NCT02654964	Glioblastoma	I	GBM [WHO grade IV]; Collection of sufficient tumor material for processing CSCs	10	Combination drug therapy	NA	Recruiting
NCT05380349	Glioblastoma	Early I	GBM (WHO grade 4); A surgically accessible to tumor mass (GBM, WHO grade 4); Not have received any prior systemic anti-cancer therapy	10	Combination drug therapy	NA	Not yet recruiting
NCT05772767	Glioma	NA	Supratentorial glioblastoma; First recurrence of a primary supratentorial glioblastoma	80	Biological sample collection; dissecting ciliogenesis players	NA	Recruiting
NCT02063893	Breast cancer	Observational	Breast cancer; Estrogen receptor and/or progesterone positive tumor	40	NA	NA	Completed
NCT04991532	Chronic myelogenous leukemia	Observational	CML-CP patients treated with TKIs	324	Drug: Dasatinib	NA	Unknown

## Drug delivery system for targeting CSCs

**Fig. 11:**
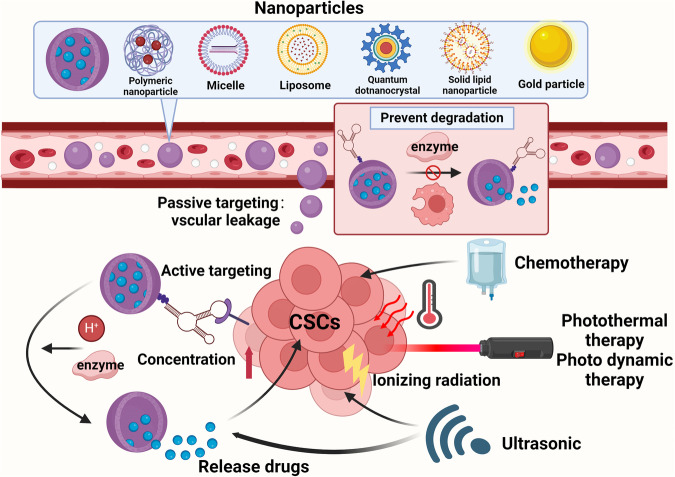
Drug delivery systems in targeting CSCs therapy. The utilization of drug delivery systems, predominantly nanomaterials, plays a pivotal role in targeting CSCs therapy. Traditional passive targeting relies on the leakage of immature blood vessels. However, advancements in technology have enabled the attainment of active targeting of nanoparticles through surface modifications. Nanomaterials, serving as carriers, offer the capacity to encapsulate therapeutic agents such as small interfering RNA (siRNA) and drugs, thus safeguarding against drug degradation. Moreover, active targeting facilitated by nanomaterials enhances drug concentration and enables precise identification of CSCs. Furthermore, nanoparticles can be stimulated both internally and externally to trigger drug release, with these triggering factors potentially doubling as therapeutic strategies

Therapies targeting CSCs face several obstacles. Traditional CSC-targeting drugs have shown significant progress, yet they suffer from shortcomings such as poor solubility, stability, and dose-limiting toxicity.^950^ Additionally, CSCs present a unique challenge in cancer treatment, displaying heightened resistance compared to ordinary tumor cells due to their robust capability of drug concentration regulation and metabolism.^951,952^ Addressing these challenges, a notable trend in targeted CSCs therapy involves drug delivery systems to optimize therapeutic effects and overcome treatment resistance.^953–955^ Targeting CSCs treatment predominantly utilizes nanoparticles, liposomes, and polymer micelle, while pH-sensitive capsules, and aptamers are also prevalent (Fig. 11).^956–958^ Moreover, other treatments include echogenic PEGylated PEI-loaded microbubble, virus preparations, multinuclear complexes, etc.^959–962^ Nanobiotechnology not only aids in early detection and tumor diagnosis but also offers several advantages in CSC treatment, including precise targeting, high-dose administration, multiple drug delivery, and controlled drug release.^963^

Precise targeting is crucial in nanotechnology-based therapies. Previously, passive targeting of tumor cells relied on exploiting vascular leakage within the tumor microenvironment to facilitate the accumulation and release of nanomaterials at tumor sites. However, advancements in technology have enabled active targeting strategies, wherein nanoparticles are equipped with targeting ligands for specific recognition of tumor cells. This active targeting mechanism enhances nanoparticle accumulation in close proximity to the tumor, thereby augmenting cellular uptake of therapeutic agents. Such precision targeting serves as the foundation for achieving localized, high-dose drug delivery.^964,965^ For instance, HA-mediated Fe_3_O_4_ nanocubes exhibit selective recognition of liver CSCs via the HA-CD44 receptor ligand pathway, effectively inhibiting their migration and proliferation.^966^ Wang et al. have developed a peptide-based drug delivery system characterized by deep tissue penetration and enhanced cellular uptake. This system, when combined with platinum, enhances radiation-induced DNA damage, thereby overcoming CSC-mediated radiation resistance.^967^ Moreover, nanocomposites such as H-MnO_2_@(ICG + ISL)@HA were a monodispersed hollow structure of MnO_2_ with a continuously modified mesoporous shell structure of HA. These nanocomposites can effectively deliver isoliquiritigenin at high concentration to CD44^+^ CSCs. H-MnO_2_@(ICG + ISL)@HA nanocomposites integration with chemotherapy or phototherapy synergistically enhances tumor eradication with minimal side effects.^968^ Conjugation with antibodies, peptides or aptamers improves CSCs recognition. For instance, Toshiyama et al. developed a poly (ethylene glycol)-poly (lysine) block copolymer-ubenimex conjugate, which enhances the production of ROS to selectively eradicate CSCs by inhibiting aminopeptidase N.^969^ Micellar nanomedicine of cisplatin, coupled with cyclic Arg-Gly-Asp peptide, exhibits enhanced inhibition of CSCs.^970^ To address challenges like off-target effects and rapid degradation, Xu et al. engineered peptide-modified nanoparticles for targeted delivery to laryngeal CSCs.^971^ Similarly, activated carbon nanoparticles loaded with metformin effectively elevate drug concentrations within liver CSCs, enhancing therapeutic efficacy.^972^ Furthermore, nucleic acid aptamers, often referred to as “chemical antibodies”, possess specific tertiary structures that bind molecular targets with high affinity. Due to their lower immunogenicity and small volume, aptamers have emerged as promising tools for CSCs targeting, especially when combined with siRNA and miRNA.^973^ Beyond precise targeting, nanoparticles can also induce the expression of tumor molecules, offering additional avenues for therapeutic intervention.^974^

High-dose administration. Nanomaterials provide a relatively stable environment for drugs, siRNA, etc., which enable prolonged drug circulation within the body. For example, CD44v6-targeted polymeric micelles loaded with niclosamide exhibit tumor-specific accumulation, allowing for increased intravenous dosages without a corresponding increase in adverse events.^975^ Furthermore, studies by Yuan et al. have demonstrated that although the plasma concentration of albumin nanoparticles carrying paclitaxel is 3–5 times lower than that of free paclitaxel, the tumor/plasma concentration ratio can reach up to 10 times higher. This underscores the specific tumor targeting capability of albumin nanoparticles and provides robust evidence supporting their suitability for high-dose administration.^976^ In vivo delivery of therapeutic molecules such as siRNA and miRNA face numerous challenges, including enzymatic degradation, interactions with blood components, and non-specific cellular uptake. In addition to enhancing therapeutic efficacy through nano-loaded drugs, loading therapeutic miRNAs like miR34a and miR200c can further augment treatment outcomes.^977,978^

Controlled drug release mechanisms rely on both endogenous and exogenous stimuli. Endogenous stimuli responses encompass pH variations, redox reactions, enzyme activity, etc. pH-sensitive nanomaterials maintain stability under physiological conditions but rapidly degrade in the acidic tumor microenvironment, facilitating targeted drug release and enhancing therapeutic efficacy.^979^ For instance, nanoparticles encapsulating SchB exhibit pH-sensitive release properties and reverse multidrug resistance in breast CSCs by inhibiting P-Glycoprotein.^957^ pH-sensitive core-shell nanoparticles can simultaneously target GSCs and differentiated cells, significantly reducing the proportion of CSCs.^980^ Exogenous stimuli, including temperature, light, and ionizing radiation etc., serve as triggers for nanomaterials and can exert therapeutic effects on tumors. Combination therapy, integrating conventional anticancer treatments with anti-CSC drugs, represents a prudent approach to enhance treatment effect.^981,982^ Photothermal or photodynamic therapy offers higher selectivity, lower toxicity, and improved reproducibility.^951^ For instance, Zhu et al. engineered nanoparticles with sheddable PEG shells and acid-activatable pro-penetration peptides to deliver a diradical-featured croconium-based photothermal agent and a natural cytotoxic HSP inhibitor to CSCs, achieving synergistic thermo-chemotherapy.^983^ Fernandes et al. designed magnetic nanoparticles released via hyperthermia to exert potent inhibitory effects on colorectal CSCs when combined with chemotherapy.^984^

Enhancing drug delivery systems to accommodate multiple drugs is crucial for inhibiting CSCs. CSCs possess unique metabolic pathways and often exhibit overexpression of drug efflux pumps, leading to multidrug resistance. Multiple dosing strategies can increase drug concentrations and target CSCs through diverse mechanisms. For example, coating cisplatin and disulfiram with hydroxypropyl-β-cyclodextrin enhances solubility, inhibiting tumor stemness and improving chemotherapy resistance.^985^ Liposomes coated with bufalin and doxorubicin effectively suppress the self-renewal of breast CSCs.^986^ Dual-targeting nanoparticles, characterized by excellent biocompatibility and precise CSC recognition, can simultaneously deliver doxorubicin and siRNA cocktails, exerting potent anti-CSC effects.^987^ Zhang et al. devised mesoporous silica nanoparticles co-loaded with multiple siRNAs, which effectively treat leukemia when combined with chemotherapy drugs.^988^ Furthermore, the combined delivery of salinomycin and docetaxel via dual-targeting gelatinase nanoparticles demonstrates significant inhibition of cervical CSCs.^989^ Nanoparticles incorporating penetration peptide RW9, an Histone Deacetylase (HDAC) inhibitor warhead, and 5-fluorouracil, along with AS1411, enhance inhibitory efficiency against stem-like cells.^990^ These advancements in drug delivery systems hold promise for combating CSC-mediated resistance and improving cancer treatment outcomes.

Various drug delivery systems exhibit both advantages and limitations.^991^ In preclinical research, nanotechnology is frequently employed to target CSCs markers such as CD44 and CD133, as well as signaling pathways like WNT/β-Catenin, Notch, and hedgehog.^991,992^ However, in current clinical trials, the utilization of inhibitors of CSCs markers or related molecular pathways is more prevalent, with limited investigations focusing on enhancing CSCs-targeted therapy through drug delivery systems. Given the heterogeneity of CSCs and the complexity of the tumor microenvironment, achieving precise targeting of CSCs remains a critical challenge. Accurate targeting not only enhances efficacy but also mitigates side effects. Furthermore, leveraging computer technology to assist in setting specific triggers for controlling drug release and identifying precise and efficient targets, employing multi-target, multi-function, and multi-drug combination strategies, will enhance the efficiency of CSC targeting.^993–998^ Designing diverse nanomaterials based on the five fundamental characteristics of nanoparticle therapy—long circulation, tumor accumulation, deep penetration, cellular internalization, and controlled drug release—remains the prevailing research paradigm.^999^ Although most studies are currently confined to preclinical investigations, optimized therapeutic strategies targeting CSCs via drug delivery systems hold significant promise.^952,1000^

In tackling brain tumors, particularly gliomas and brain metastases, optimizing drug delivery systems is essential due to the inherent limitations of chemotherapy, including lack of specificity, harmful side effects, low efficacy, and limited transport.^1001^ The blood-brain barrier (BBB) high selectivity for permeating substances, the unique brain microenvironment, and the deep-seated location of GSCs, possess robust chemotherapy resistance for GSCs.^1002^ Overcoming these obstacles and effectively targeting CSCs is pivotal. Knauer et al. demonstrated in vitro inhibition of GSCs and modulation of tumor cell surface markers such as PD-L1, TIM-3, and CD47 using a polycationic phosphorus dendrimer-based approach for siRNAs.^1003^ Aptamer technology has also shown promise in GSCs. Behrooz et al. reported that B19 aptamer-conjugated PAMAM G4C12 dendrimer nanoparticles simultaneously deliver paclitaxel and temozolomide into U87 CSCs, effectively eliminating U87 CSCs without toxic side effects.^1004^ Multi-drug therapy by employing nanotechnology is gaining traction. Smiley et al. utilized functionalized nanoparticles to co-deliver TMZ and the MDM2 inhibitor idasanutlin to target GSCs.^1005^ Gold nanoparticles releasing retinoic acid and TMZ upon low-intensity ultrasound stimulation sensitize GSCs to chemotherapy.^1006^ Nanostructured lipid carriers co-deliver paclitaxel and doxorubicin to inhibit GSCs proliferation via PI3K/AKT/mTOR signal pathway.^1007^ Peptides also exhibit anti-GSCs properties. Multifunctional tandem peptide R8-c (RGD) destroys vasculogenic mimicry to suppress GSCs proliferation.^1008^ Additionally, functional curcumin liposomes, layered double hydroxide nanoparticles, and other formulations demonstrate therapeutic efficacy against GSCs.^1009^

Moreover, nanoparticles or liposomes capable of penetrating the BBB may play a pivotal role. Engineered high-density lipoprotein-mimetic nanoparticles effectively deliver SHH inhibitors to stem-like cells in medulloblastoma.^1010^ Lu et al. synthesized folic acid-modified albumin nanoparticles to enhance BBB permeability and cellular uptake. These nanoparticles loaded with paclitaxel and autophagy inhibitor chloroquine effectively inhibit GSCs.^1011^ Curcumin-loaded chitosan-poly (lactic-co-glycolic acid) nanoparticles, processing with sialic acid to enhance BBB permeability and target the brain CSCs via anti-ALDH, demonstrate therapeutic potential.^1012^ Furthermore, liposomes capable of crossing the BBB induce necrosis, apoptosis, and autophagy in glioma and GSCs.^1013^ These advancements offer promising avenues for combating brain tumors and targeting CSCs effectively.

Séhédic et al. reported that radiopharmaceutical nanoparticles penetrated the BBB and demonstrated therapeutic efficacy against glioblastoma in mice.^1014^ Despite numerous drug delivery systems proving effective in inhibiting tumors by traversing the BBB, studies targeting CSCs remain relatively scarce.^1015,1016^ Although few treatments targeting GSCs have reached clinical trials, continued research into potential pathways and treatment strategies is imperative.^1001^ Mechanical or chemical disruption of the BBB via MRI-guided focused ultrasound, convection-enhanced diffusion, microdialysis catheters, hypertonic agents, hydrophilic surfactants, and other methods have been explored to modulate BBB permeability. However, the BBB serves as a highly selective diffusion barrier, shielding the brain from toxins and other blood compounds.^1017^ Balancing the beneficial opening of the BBB for drug delivery with the preservation of its protective barrier function poses a critical question. Brain tumors such as GBM, brain parenchymal metastasis, and leptomeningeal metastasis exhibit high malignancy, with patients experiencing extremely short survival times. While technologies like intrathecal injection and Omaya reservoir enable localized treatment of the nervous system and increase drug concentration, their potential combination with CSCs-targeting approaches requires further investigation.^1018–1020^ Phase I/II clinical trials (NCT03566199) have demonstrated the safety of the panobinostat nanoparticle formulation MTX110 for newly-diagnosed diffuse intrinsic pontine glioma. However, nanotechnology specifically targeting brain CSCs remains an area lacking in research.

## Challenges in cancer stem cell research

Given the pivotal role of CSCs in tumor relapse and resistance mechanisms, extensive research efforts are being dedicated to the task of identifying and targeting CSCs. However, the identification of CSC-specific antigens or biomarkers remains a formidable challenge. Potential CSC biomarkers, identified through aberrant signaling and metabolic pathways, can be broadly classified into two categories: cell surface markers and intracellular markers. Cell surface markers, particularly transport proteins, and signaling receptors have garnered attention for their potential to facilitate the diagnostic and precise delivery of therapeutic agents to CSCs.^1021,1022^ Yet, the non-specificity and low abundance of these markers pose significant obstacles to their practical application. The surface markers identified to date lack specificity for any single CSC type, as they are also expressed on non-CSCs or healthy cells, albeit at lower levels.^1023^ Large libraries of intracellular molecules may reveal concentration differences between CSCs and other cell populations, overexpressed intracellular enzymes in CSCs remain key molecular targets for CSC-specific strategies. These enzymes, exemplified by ALDH, can be targeted with prodrugs activated in the presence of specific enzymes, thereby preferentially killing CSCs.^1024^ Additionally, transcription factors regulating CSC proliferation and differentiation, such as BMI-1 and c-Myc, offer avenues for the design of inhibitors to induce CSC apoptosis.^1025,1026^ Other crucial CSC-related transcription factors, essential for maintaining CSC tumorigenicity and stemness, like OCT3/4 and SOX2, have also garnered widespread interest.^1027,1028^ However, a critical issue is that these intracellular transcription factors are not unique to CSCs, as most signaling and metabolic pathways are shared among CSCs, non-stem cells, and healthy cells.

Secondly, although existing CSCs-target therapy shows promise in cancer treatment, numerous limitations persist. CD133, as a potential molecular target, poses challenges in terms of reliable detection and specific antibody recognition.^47^ Its expression is influenced by various factors, including oxygen levels, cell density, and cell cycle, all of which can affect its protein expression within the microenvironment. Currently, detection of CD133 primarily relies on immunohistochemistry and flow cytometry, both of which require specific antibodies. However, CD133 is sensitive to glycosylation modifications, potentially impacting antibody binding. Commonly used CD133 antibody clones, including CD133/1 (AC133 or W6B3C1) and CD133/2 (AC141 or 293C3), recognize different glycosylated epitopes in the CD133 EC3 region. Yet, glycosylation differences may lead to selective splicing and masking of epitope binding sites, thereby reducing detection accuracy.^40^ Additionally, it is noteworthy that both CD133^+/−^ cancer cells can initiate tumors, raising questions about the validity of current CSC biomarkers as true tumor-initiating cells.^1029^ Recent studies have also discovered that CSCs exhibit high plasticity, capable of phenotypic transitions under specific conditions. For instance, in xenograft mouse cancer organoids, gene knockout of LGR5^+^ CSCs can limit tumor growth but not eliminate it. Tumors can be sustained by proliferative LGR5^−^ cells and, upon cessation of the knockout, LGR5^+^ CSCs reemerge, leading to rapid tumor regeneration.^1030^ This suggests that tumor cells with a higher degree of differentiation, following CSC depletion, possess plasticity to revert to the CSC state to compensate for CSC loss. Furthermore, CSC niches created by different cells within the microenvironment can facilitate the evolution of distinct CSC dominant clones.^1031^ However, current research on the microenvironment and CSCs relies heavily on tumor implantation analyses in mouse models, which cannot fully replicate the microenvironment of primary tumors and the interactions between human CSCs and their microenvironment, thus introducing certain limitations.

Given the challenges associated with CSCs-target therapy, combination therapy emerges as a promising strategy to eradicate CSCs and thereby improve patient outcomes.^1032^ Combination therapy is recognized for its potency, as it targets multiple pathways to effectively address tumor heterogeneity and enhance efficacy. Moreover, the concurrent use of multiple drugs can tackle drug resistance, aiding in the elimination of CSCs.^1033^ Although traditional chemotherapy may not directly target CSCs, its foundational and critical role in treating various cancers, especially in early-stage patients, cannot be overlooked. Perhaps their combination with other CSCs-target therapy could overcome the issue of relapse.^1034^ When applying combination therapy in clinical practice, several issues need to be considered.^1035^ Firstly, drug interactions may affect efficacy, with one drug potentially interfering with the metabolic activity of another, thereby reducing overall effectiveness. Additionally, the pharmacokinetics of concurrent administration become exceedingly complex due to differences in drug metabolism and uptake. Secondly, the combined use of multiple drugs might provoke cumulative side effects, complicating the assessment of treatment dosages. If two drugs have similar side effects, this could negatively impact patient survival. Identifying the specific drug responsible for side effects also poses a challenge, sometimes necessitating the cessation of all drugs. Lastly, CSCs might acquire an MDR phenotype through mechanisms such as overexpression of drug efflux pumps, alterations in DNA repair mechanisms, and modulation of cell death pathways, rendering combination therapy ineffective.^611–613^ However, a judicious sequence of combination therapy could delay the onset of resistance, thus eliminating CSCs before they become drug-resistant. Therefore, developing a rational sequence of combination therapy is crucial. Moreover, devising novel and effective methods for targeting CSCs remains a focus of research.^1036^ Nanotherapy, as a potential strategy, offers possibilities for sustained treatment by enhancing drug specificity for CSCs, reducing off-target effects, increasing drug load, optimizing penetration of biological barriers, and controlling drug release.^1037^ Additionally, nanomaterials can carry multiple therapeutic agents, achieving synergistic effects and potentially reducing resistance. Improved pharmacokinetic properties and protection against enzymatic degradation further consolidate the status of nanomaterials as an effective and versatile platform for targeting CSCs.

Currently, the most prominent and likely direction for the next decade revolves around identifying novel CSC-specific biomarkers and leveraging them for cancer treatment. There’s an active exploration into introducing artificial biomarkers into CSCs. Metabolic glycoengineering of unnatural sugars offers a straightforward tool for incorporating artificial chemical receptors into the cell membrane for subsequent targeting purposes. One such example, the azidosugar AAMCO, can label cells in an ALDH1A1-activated manner, thus preferentially tagging CSCs overexpressing ALDH1A1 with azide groups. This method transforms intracellular ALDH1A1 into a clickable tag on the cell surface, paving a new pathway for developing CSC-targeting technologies.^1038^ In principle, other unnatural sugars that can be reactivated by other overexpressed CSC enzymes could also be used for CSC labeling and targeting. However, a challenge is the relatively low labeling efficiency of AAMCO, as the ALDH1A1 response is partly at the expense of overall metabolic labeling efficiency. A delicate balance between labeling efficiency and selectivity is necessary. This issue could be mitigated by deploying strategies to improve the delivery of enzyme-activatable unnatural sugars to tumors, such as using nanoparticles. Metabolic lipid labeling could also serve as an alternative method for chemical labeling of CSCs.^1039^ Given the aberrant lipid metabolism in CSCs, rational design of unnatural lipids based on structural lipids (like dioleoylphosphatidylcholine, DOPC) or signaling lipids (like ceramides, phosphatidylinositol lipids) could achieve preferential labeling of CSCs over non-stem cancer cells or healthy cells. Chemical tags, such as azide groups, could then target therapeutic drugs to CSCs.^1040^ Compared to metabolic glycoengineering pathways involving multiple reactions, lipid metabolism pathways involve fewer steps and may lead to higher labeling efficiency.^1041^ Similar to metabolic glycan labeling, strategies to improve the delivery of unnatural lipids to tumors and enhance CSC uptake of unnatural lipids could further increase labeling efficiency and subsequent CSC targeting efficacy.

Despite the numerous challenges faced in CSC research, ongoing studies and technological advancements offer hope for a deeper understanding of the nature of cancer. Such progress promises to unveil novel cancer treatment strategies by navigating the complexities of tumor biology to reveal new avenues of intervention.

## Conclusion

Although CSCs originate from differentiated cells, normal stem/progenitor cells, or hybrids from cell-cell fusion, environmental factors in CSC niches are essential in the formation and maintenance of CSCs. Characterized by self-renewal and pluripotency, CSCs play pivotal roles in cancer initiation, proliferation, metastasis, and therapeutic resistance. Identification of CSCs relies on specific biomarkers, including intracellular and cell-surface markers, which serve as tools to predict patients’ prognosis regarding specific treatments. Multiple signaling pathways are excessively activated in CSCs, with intricate crosstalk among them. Extensive evidence indicates that the activation of pathways such as WNT/β-Catenin, hedgehog, Notch, NF-κB, JAK/STAT, TGF-β, PI3K/AKT, and PPAR contributes to various malignant behaviors of CSCs, offering potential therapeutic targets.

Despite facing numerous challenges, researchers remain dedicated to exploring innovative approaches to eradicate CSCs, thereby enhancing the responsiveness to chemotherapy. In-depth research into the characteristics of CSCs, including novel markers, signaling pathways, and the microenvironment, is expected to be a hot theme in the coming decades. Although there is currently a lack of sufficient high-quality clinical trials to confirm the efficacy of these strategies, the deepening of research gives us reason to anticipate the discovery of more effective methods for eliminating CSCs to overcome chemotherapy resistance in the future. The rapid advancement of immunotherapy has ushered in a new era in anti-tumor treatment. In-depth investigations into the immunological characteristics of CSCs have laid a theoretical foundation for immunotherapy targeting CSCs and validated its technical feasibility. Extensive preclinical research has thoroughly demonstrated the potential benefits of CSC-targeted immunotherapy. However, to translate this approach into clinical practice, we still face a host of challenges. These include identifying specific antigens of CSCs, unraveling the mechanisms by which CSCs evade immune surveillance, and understanding the impact of the immunosuppressive tumor microenvironment on therapy. Addressing these issues may pave the way toward successful anti-tumor treatment.

Radiation can induce CSC formation, and CSCs are generally radioresistant. Although it is mechanically reasonable to target CSCs to improve radiosensitivity, few clinical studies succeeded. While preclinical studies consistently demonstrate that the presence of CSCs correlates positively with resistance to targeted therapies, their eradication has the potential to reverse this resistance. However, clinical trials currently lack supportive evidence for this proposition. Therapeutic approaches aimed at eliminating CSCs primarily involve targeting CSC markers, signaling pathways, and their microenvironmental niche. Although numerous clinical trials have been undertaken, substantial, high-quality clinical evidence supporting the efficacy of these strategies remains elusive.

CSCs, a rare subset within tumors, exert significant influence over various malignant processes, including tumor initiation, proliferation, and metastasis. Notably, CSCs exhibit resistance to diverse therapeutic interventions, encompassing chemotherapy, immunotherapy, radiotherapy, and targeted therapy. Comprehensive understanding of CSCs’ involvement in treatment resistance, coupled with strategies to specifically target CSCs, is crucial for advancing patient outcomes. Preliminary findings from preclinical studies indicate the potential efficacy of CSC-targeted interventions in overcoming treatment resistance. Moreover, the forthcoming results from ongoing clinical trials hold promise for further advancing the understanding and therapeutic management in this field.
